# The comprehensive summary of surgical versus non-surgical treatment for obesity: a systematic review and meta-analysis of randomized controlled trials

**DOI:** 10.18632/oncotarget.9581

**Published:** 2016-05-24

**Authors:** Ji Cheng, Jinbo Gao, Xiaoming Shuai, Guobin Wang, Kaixiong Tao

**Affiliations:** ^1^ Department of Gastrointestinal Surgery, Union Hospital, Tongji Medical College, Huazhong University of Science and Technology, Wuhan, Hubei, China

**Keywords:** obesity, bariatric surgery, meta-analysis, systematic review, Pathology Section

## Abstract

**Background:**

Bariatric surgery has emerged as a competitive strategy for obese patients. However, its comparative efficacy against non-surgical treatments remains ill-defined, especially among nonseverely obese crowds. Therefore, we implemented a systematic review and meta-analysis in order for an academic addition to current literatures.

**Methods:**

Literatures were retrieved from databases of PubMed, Web of Science, EMBASE and Cochrane Library. Randomized trials comparing surgical with non-surgical therapies for obesity were included. A Revised Jadad's Scale and Risk of Bias Summary were employed for methodological assessment. Subgroups analysis, sensitivity analysis and publication bias assessment were respectively performed in order to find out the source of heterogeneity, detect the outcome stability and potential publication bias.

**Results:**

25 randomized trials were eligibly included, totally comprising of 1194 participants. Both groups displayed well comparability concerning baseline parameters (*P* > 0.05). The pooled results of primary endpoints (weight loss and diabetic remission) revealed a significant advantage among surgical patients rather than those receiving non-surgical treatments (*P* < 0.05). Furthermore, except for certain cardiovascular indicators, bariatric surgery was superior to conventional arms in terms of metabolic secondary parameters (*P* < 0.05). Additionally, the pooled outcomes were confirmed to be stable by sensitivity analysis. Although Egger's test (*P* < 0.01) and Begg's test (P<0.05) had reported the presence of publication bias among included studies, “Trim-and-Fill” method verified that the pooled outcomes remained stable.

**Conclusion:**

Bariatric surgery is a better therapeutic option for weight loss, irrespective of follow-up duration, surgical techniques and obesity levels.

## INTRODUCTION

Emerging as a costly burden of global healthcare system, obesity has currently attracted worldwide attentions due to its uncontrollably rising incidence, especially in industrialized countries [1]. Economically, the annual expense directly linked to overweight (body mass index 25-30) and obesity (body mass index > 30) is estimated to be almost 16 billion pounds globally [2]. During the past three decades, the epidemiological prevalence of obesity has quadrupled to 25% among UK population, including 2.4% of which are affected by morbid obesity (body mass index > 40) [3]. At present, it is appraised that approximately two thirds of overall population in US have been clinically diagnosed as overweight or obese [4]. Characterized by excessive adipose storage, obesity is commonly accompanied by a variety of comorbidities, mainly comprising of type 2 diabetes mellitus, hypertension and cardiovascular accidents. As an integrative product of multifactorial impacts, a definite etiological explanation of obesity remains obscure, leading to the absence of effective strategies targeting its development [5].

According to SAGES [6] and NICE [7] guidelines, multicomponent interventions are currently the treatment of choice, including lifestyle intervention, dietary restriction, pharmaceutical and surgical management. Despite of its first-line status, non-surgical treatments lead to poor compliance and unfavorable endpoint satisfaction among obese patients. As is reported, the glycemic control of obesity-related diabetes is merely accomplished amid 40% of patients undergoing conventional medications [8]. Meanwhile, lifestyle reformation fails to reduce the probability of obesity-associated lethality as well as the hazards of cardiovascular accidents among obese crowds [9].

Bariatric surgery, initially reported in 1995 [10], has been regarded as an effective supplement to current regimen, especially for those suffering from severe obesity (body mass index > 40) [6, 7]. By mechanically altering the physiological mode of gastrointestinal absorption, bariatric surgery triggers remarkable decline on excessive weight, hyperglycemia, cardiovascular risk and correlative mortality compared to conservative therapeutics [4, 11, 12]. Moreover, by meta-analyzing short-term observational studies, Muller et al [8] has investigated that in contrast to non-surgical remedies, bariatric surgery favorably produces therapeutic benefit among patients with nonsevere obesity (body mass index 30-35), which is a vital addition to current guidelines that surgical intervention is recommended for patients with body mass index > 35, especially the morbidly obese sufferers (body mass index > 40) [6]. Therefore, the updated NICE guidance (CG189) has broadened the indications that as a second-line option, all patients with body mass index > 30 should be assessed for bariatric surgery following the refractoriness to non-surgical interventions [2].

Nevertheless, long-term (3-year or more) efficacy of surgical *versus* non-surgical interventions is rarely described, especially lacking of a well summarized evidence. Additionally, the clinical value of bariatric surgery for nonseverely obese patients requires further analysis. Hence we performed this systematic review and meta-analysis in order to comprehensively make comparisons between both strategies, aiming to provide novel evidences for future guidelines.

## RESULTS

### General characteristics

**Figure 1:**
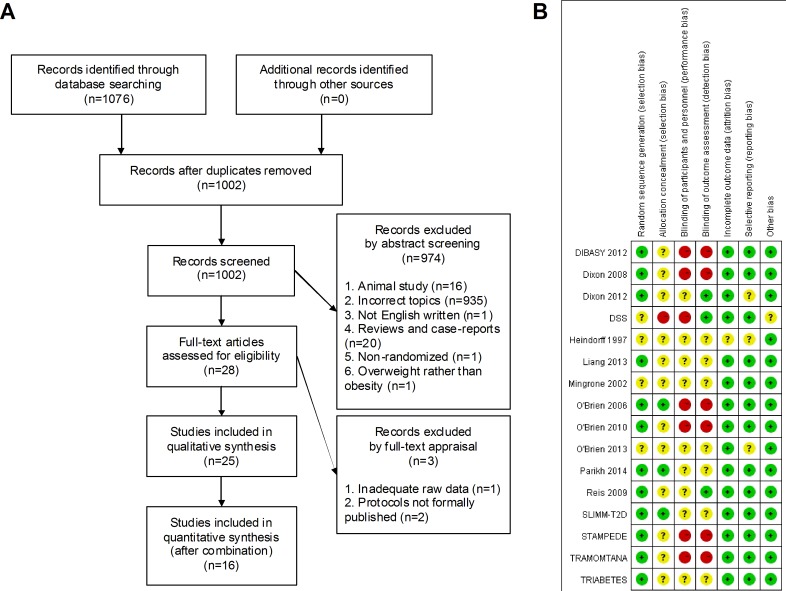
Selection flow chart and risk of bias summary **A.** Flow chart of the entire selection process; **B.** Risk of bias summary.

The preliminary 1076 entries were rigorously screened to generate 25 eligible studies for pooling analysis, with a total amount of 1194 participants and individually ranging from 16 to 150 (Figure 1A). Merely 2 studies were performed by developing nations while 14 trials originated from industrialized countries, each of Australia and US accounting for the maximum amount of 5. None of the included trials featured adolescent subjects except for O'Brien 2010. Female patients dominated the sexuality proportion towards male counterparts, with a ratio of 740 to 454. Among the included trials, in general, it was mutually comparable between both comparative interventions regarding the baseline confounding elements (Table 1).

**Table 1:** Baseline features of included studies

Trial title	Country	Trial registration	Group	Sample size	Age (years)	Sexuality (M/F)	BMI (kg/m^2^)	Weight (kg)	Waist circumference (cm)	Caucasian	Hypertension	T2DM (Prevalence/Duration)
DIBASY[13,14]	Italy	NCT00888836	Surgery	40	43.3±7.7	18/22	45.0±6.5	133.8±26.7	127.9±18.2	NA	NA	100.0%/6.0y
			Control	20	43.5±7.3	10/10	45.6±6.2	136.4±21.9	126.9±14.7	NA	NA	100.0%/6.1y
Dixon 2008[15]	Australia	ACTRN12605000159651	Surgery	30	46.6±7.4	15/15	37.0±2.7	105.6±13.8	114.1±10.2	NA	28(93.3%)	100.0%/<2y
			Control	30	47.1±8.7	13/17	37.2±2.5	105.9±14.2	116.0±10.0	NA	27(90.0%)	100.0%/<2y
Dixon 2012[16]	Australia	ACTRN12605000161628	Surgery	30	47.4±8.8	17/13	46.3±6.0	134.9±22.1	136.1±13.1	NA	15(50.0%)	33.3%/NA
			Control	30	50.0±8.2	18/12	43.8±4.9	126.0±19.3	126.6±13.1	NA	17(56.7%)	33.3%/NA
DSS[17,18]	USA	NCT00641251	Surgery	60	49.0±9.7	22/38	34.9±2.9	98.8±13.2	114.0±9.7	33(55.0%)	NA	100.0%/8.9y
			Control	60	49.0±7.7	26/34	34.3±3.1	97.9±16.3	113.0±11.6	30(50.0%)	NA	100.0%/9.1y
Heindorff 1997[19]	Denmark	NA	Surgery	8	22.0-41.0	2/6	43.0-54.0	NA	NA	NA	NA	NA/NA
			Control	8	21.0-43.0	7/1	40.0-56.0	NA	NA	NA	NA	NA/NA
Liang 2013[20]	China	NCT01435980	Surgery	31	50.8±5.4	22/9	30.5±0.9	82.0±3.5	NA	0(0.0%)	31(100.0%)	100.0%/7.4y
			Control	70	51.4±6.3	48/22	30.3±1.7	81.5±4.4	NA	0(0.0%)	70(100.0%)	100.0%/7.2y
Mingrone 2002[21]	Italy	NA	Surgery	46	30.0-45.0	7/39	48.2±5.0	133.9±16.2	NA	NA	0(0.0%)	0.0%/0.0y
			Control	33	30.0-45.0	4/29	48.2±7.7	130.9±24.5	NA	NA	0(0.0%)	0.0%/0.0y
O'Brien 2006[22]	Australia	ACTRN12605000113651	Surgery	40	41.8±6.4	10/30	33.7±1.8	96.1±11.2	103.3±10.0	NA	9(22.5%)	NA/NA
			Control	40	40.7±7.0	9/31	33.5±1.4	93.6±11.9	99.4±9.4	NA	7(17.5%)	NA/NA
O'Brien 2010[23]	Australia	ACTRN12605000160639	Surgery	25	16.5±1.4	9/16	42.3±6.1	120.7±25.3	120.8±14.2	NA	NA	NA/NA
			Control	25	16.6±1.2	7/18	40.4±3.1	115.4±14.0	118.1±10.6	NA	NA	NA/NA
O'Brien 2013[24]	Australia	ACTRN12611000279921	Surgery	31	53.6±6.2	5/26	33.6±1.9	94.7±11.2	103.2±10.1	NA	NA	NA/NA
			Control	20	52.7±7.7	7/13	33.5±1.5	95.6±13.4	101.5±11.1	NA	NA	NA/NA
Parikh 2014[25]	USA	NCT01423877	Surgery	29	46.8±8.1	6/23	32.8±1.7	81.9±7.7	106.3±10.1	25(86.2%)	NA	100.0%/NA
			Control	28	53.9±8.4	6/22	32.4±1.8	83.7±10.8	106.7±7.7	25(89.3%)	NA	100.0%/NA
Reis 2009[26]	Brazil	NA	Surgery	10	36.7±11.5	10/0	55.7±7.8	168.6±28.2	NA	NA	NA	NA/NA
			Control	10	42.2±11.0	10/0	54.0±6.1	160.4±20.1	NA	NA	NA	NA/NA
SLIMM-T2D[27,28]	USA	NCT01073020	Surgery	37	50.7±10.2	15/22	36.2±3.2	105.7±13.1	NA	25(67.6%)	NA	100.0%/10.5y
			Control	41	52.0±6.2	22/19	36.6±3.8	107.5±17.8	NA	23(56.1%)	NA	100.0%/9.2y
STAMPEDE[29-34]	USA	NCT00432809	Surgery	100	48.1±8.2	32/68	36.6±3.6	103.8±15.8	115.2±9.8	73(73.0%)	65(65.0%)	100.0%/8.4y
			Control	50	49.7±7.4	19/31	36.8±3.0	106.5±14.7	114.5±9.4	37(74.0%)	26(52.0%)	100.0%/8.9y
TRAMOMTANA[35]	Spain	EudraCT 2009-013737-24	Surgery	37	44.1±9.8	11/26	49.2±5.9	132.8±24.4	NA	35(94.6%)	NA	24.3%/NA
			Control	106	47.4±11.0	34/72	46.2±4.8	123.8±19.2	NA	103(97.2%)	NA	24.5%/NA
TRIABETES[36,37]	USA	NCT01047735	Surgery	46	46.8±7.0	9/37	35.5±3.0	99.7±13.3	112.7±10.2	35(76.1%)	25(54.3%)	100.0%/6.8y
			Control	23	48.3±4.7	4/19	35.7±3.3	102.6±13.8	111.7±9.5	19(82.6%)	16(69.6%)	100.0%/5.7y

Trial title: Relevant articles deriving from an identical clinical trial were mathematically combined and individually identified with an official acronym. Those trials lacking a specific abbreviation were demonstrated by surnames of the first author and publication year. M/F: male/female; BMI: body mass index; T2DM: type 2 diabetes mellitus; NA: not available; Y: years;

### Summary of methodological assessment

According to Revised Jadad's Scale, 13 trials were appraised as high-quality in methodology, while DSS, Heindorff 1997 and Mingrone 2002 were identified as low-quality investigations (Table 2).

**Table 2:** Revised Jadad's Scale assessment

Trial	Randomization	Allocation concealment	Blindness	Withdrawal	Total
DIBASY	2	1	0	1	4
Dixon 2008	2	1	0	1	4
Dixon 2012	2	1	2	1	6
DSS	1	0	0	1	2
Heindorff 1997	1	1	1	0	3
Liang 2013	2	1	1	1	5
Mingrone 2002	1	1	1	0	3
O'Brien 2006	2	2	0	1	5
O'Brien 2010	2	1	0	1	4
O'Brien 2013	1	1	1	1	4
Parikh 2014	2	2	1	1	6
Reis 2009	2	1	1	1	5
SLIMM-T2D	2	2	1	1	6
STAMPEDE	2	1	0	1	4
TRAMOMTANA	2	1	0	1	4
TRIABETES	2	1	1	1	5

Since a relatively small fraction of subitems were assessed with low risk of bias, DSS, Heindorff 1997, Mingrone 2002 and O'Brien 2013 were considered with high risk of internal bias. The overall amount of low risk subitems was 61, followed by 37 of unclear risk and 14 of high risk, implying a substantially low risk of bias within our pooled analysis (Figure 1B).

### Primary endpoints

#### Weight loss

#### Overall patients

**Figure 2:**
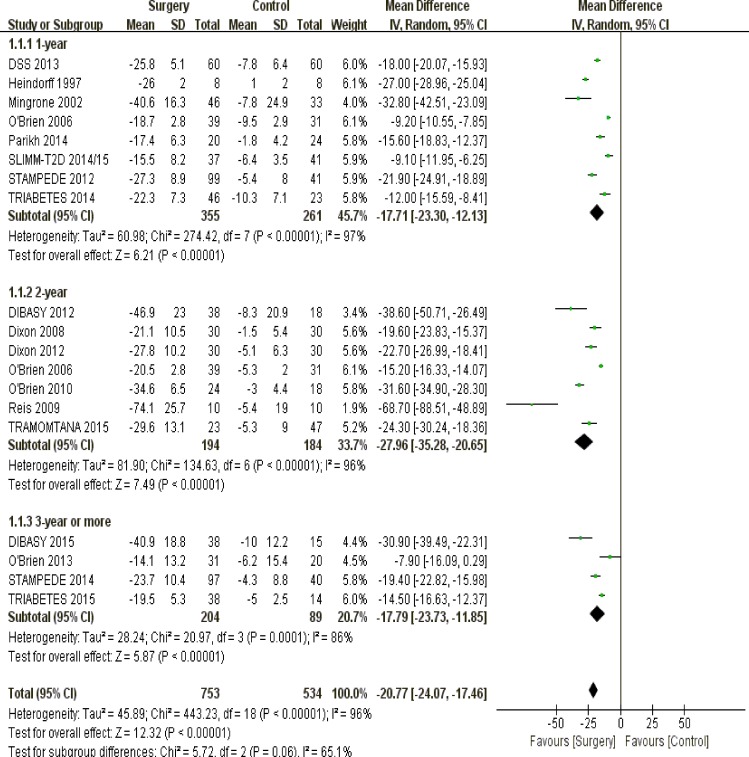
The forest plot of weight loss (kg) in terms of follow-up duration

undergoing surgical interventions had more weight loss than those receiving non-surgical managements (*P* < 0.00001) (Figure 2).

#### Follow-up duration

In terms of weight loss, surgical approach was superior to conventional strategies among patients with 1-year (*P* < 0.00001), 2-year (*P* < 0.00001) or long-term (3 years or more) follow-up duration (*P* < 0.00001) (Figure 2).

#### Surgical techniques

**Figure 3:**
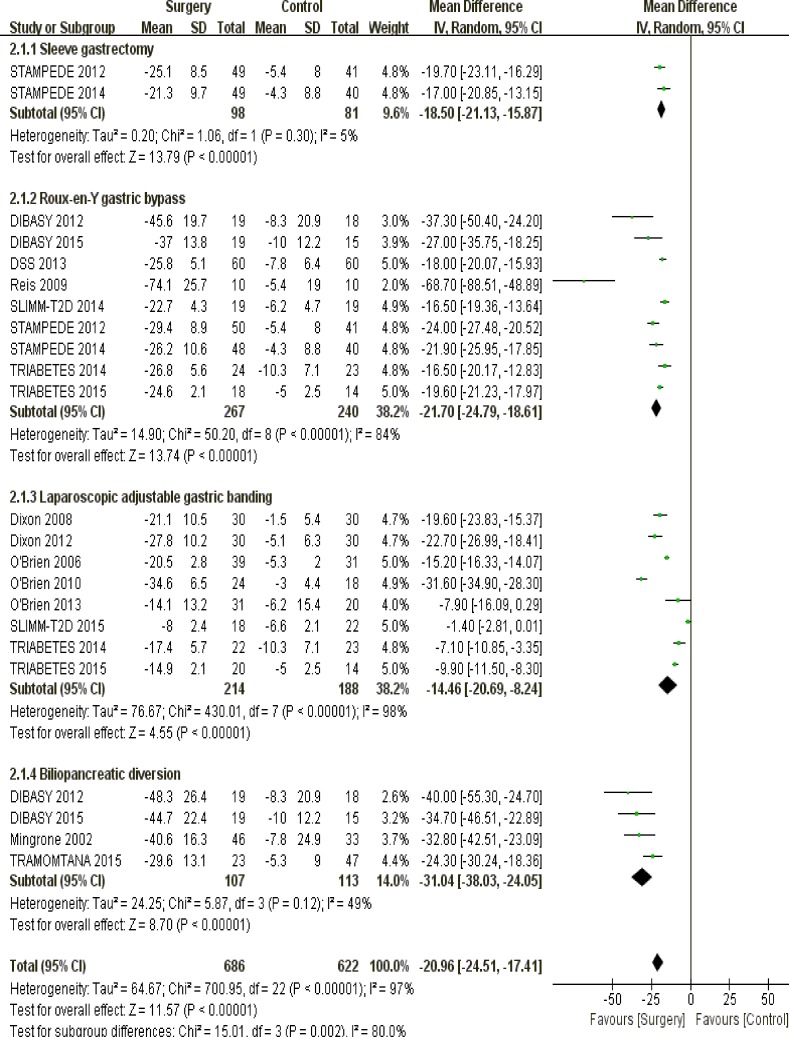
The forest plot of weight loss (kg) in terms of surgical techniques

Non-surgical therapeutics were unable to outstrip bariatric surgery regarding weight loss among obese patients, irrespective of sleeve gastrectomy (*P* < 0.00001), Roux-en-Y gastric bypass (*P* < 0.00001), laparoscopic adjustable gastric banding (*P* < 0.00001) and biliopancreatic diversion (*P* < 0.00001) (Figure 3).

#### Levels of obesity

**Figure 4:**
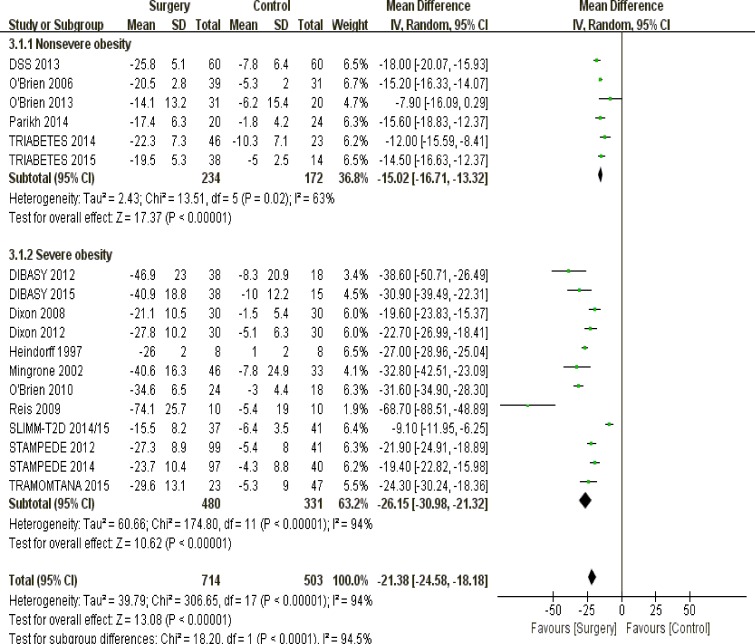
The forest plot of weight loss (kg) in terms of obesity levels

Among patients with nonsevere obesity (body mass index 30-35; *P* < 0.00001) and severe obesity (body mass index > 35; *P* < 0.00001), metabolic surgery was a more effective tool for losing weight against non-surgical treatments (Figure 4).

#### Remission of type 2 diabetes mellitus

### Overall

Our pooled outcomes suggested that surgical patients had a higher rate of diabetic remission than those undergoing non-surgical interventions (*P* < 0.00001) (Supplementary Figure S1).

#### Follow-up duration

Patients undergoing bariatric surgery achieved significantly higher rate of diabetic remission compared to recipients of conservative therapeutics, based on pooled results of 1-year (*P* < 0.0001), 2-year (*P* = 0.004) and long-term follow-up (*P* < 0.0001) (Supplementary Figure S1).

#### Surgical techniques

In contrast to conserved managements, there was a much better remission rate of diabetes amid surgical recipients, irrespective of sleeve gastrectomy (*P* < 0.00001), Roux-en-Y gastric bypass (*P* < 0.00001), laparoscopic adjustable gastric banding (*P* = 0.0008) and biliopancreatic diversion (*P* = 0.001) (Supplementary Figure S2).

#### Levels of obesity

Concerning the diabetic remission rate, both nonseverely (*P* = 0.0004) and severely obese (*P* < 0.0001) sufferers significantly benefited from surgical remedies, compared with non-surgical patients (Supplementary Figure S3).

### Secondary endpoints

#### Excessive weight loss

#### Overall

Among obese subjects, our quantitative analysis reported that operative treatments induced higher percentage of excessive weight loss against traditional therapeutics (*P* < 0.00001) (Supplementary Figure S4).

#### Follow-up duration

Regardless of 1-year (*P* < 0.00001), 2-year (*P* < 0.00001) or long-range follow-up (*P* = 0.006), bariatric surgery was far more efficient to eliminate excessive weight than non-surgical interventions (Supplementary Figure S4).

#### Surgical techniques

It was a statistical advantage to surgically reduce the excessive mass of obese enrollees instead of conventional treatments, whichever of sleeve gastrectomy (*P* < 0.00001), Roux-en-Y gastric bypass (*P* < 0.00001), laparoscopic adjustable gastric banding (*P* = 0.0008) and biliopancreatic diversion (*P* < 0.00001) was performed (Supplementary Figure S5).

#### Levels of obesity

Recipients of bariatric surgery gained greater loss of excessive weight in comparison to those undergoing non-invasive treatments, according to the subgroup analysis of both nonsevere (*P* < 0.00001) and severe obesity (*P* < 0.00001) (Supplementary Figure S6).

#### Fasting glucose

#### Overall

A better alleviation on fasting glucose was observed among patients undergoing bariatric surgery, in contrast to those receiving conventional remedies (*P* < 0.00001) (Supplementary Figure S7).

#### Follow-up duration

Regardless of 1-year (*P* = 0.0005), 2-year (*P* = 0.0003) or long-term follow-up (*P* = 0.02), surgical management was significantly effective in decreasing fasting glucose compared to non-surgical interventions (Supplementary Figure S7).

#### Surgical techniques

None of sleeve gastrectomy (*P* = 0.0005), Roux-en-Y gastric bypass (*P* < 0.00001) and laparoscopic adjustable gastric banding (*P* < 0.0001) fell behind in fasting glucose reduction against conserved therapeutics, except for biliopancreatic diversion, which was statistically equivalent to contrastive group (*P* = 0.05) (Supplementary Figure S8).

#### Levels of obesity

Compared to non-surgical regimen, both nonseverely (*P* = 0.001) and severely obese (*P* < 0.00001) patients obtained greater downregulation on fasting glucose following the surgical interventions (Supplementary Figure S9).

#### Glycated hemoglobin

#### Overall

Metabolic surgery led to more reduction on percentage of glycated hemoglobin among obese participants than conventional managements (*P* < 0.00001) (Supplementary Figure S10).

#### Follow-up duration

A higher percentage reduction on glycated hemoglobin was surgically achieved among patients with 1-year (*P* < 0.00001), 2-year (*P* = 0.02) and long-term follow-up (*P* < 0.00001), rather than those with conservative interventions (Supplementary Figure S10).

#### Surgical techniques

Irrespective of sleeve gastrectomy (*P* < 0.0001), Roux-en-Y gastric bypass (*P* < 0.00001), laparoscopic adjustable gastric banding (*P* = 0.002) and biliopancreatic diversion (*P* = 0.03), bariatric surgery was significantly superior to non-surgical approaches in terms of reducing elevated glycated hemoglobin (Supplementary Figure S11).

#### Levels of obesity

There was a greater decrease on glycated hemoglobin percentage following bariatric operations than non-surgical strategies, among nonseverely (*P* < 0.00001) and severely obese patients (*P* < 0.00001) (Supplementary Figure S12).

#### Waist circumference

#### Overall

Compared to baseline values, a greater loss on waist circumference was obtained amid surgical patients, instead of non-surgical counterparts (*P* < 0.00001) (Supplementary Figure S13).

#### Follow-up duration

Among the three subgroups of 1-year (*P* < 0.00001), 2-year (*P* < 0.00001) and long-term follow-up (*P* < 0.00001), patients that were surgically treated obtained larger decline on waist circumference than those were conventionally cured (Supplementary Figure S13).

#### Surgical techniques

Bariatric surgery was more effective to cut down waist circumference than non-surgical interventions, including sleeve gastrectomy (*P* < 0.0001), Roux-en-Y gastric bypass (*P* < 0.00001), laparoscopic adjustable gastric banding (*P* < 0.00001) and biliopancreatic diversion (*P* < 0.00001) (Supplementary Figure S14).

#### Levels of obesity

Based on the pooled outcomes featuring nonseverely (*P* < 0.00001) and severely obese sufferers (*P* < 0.00001), more reduction on waist circumference was observed following surgical management in contrast to traditional treatments (Supplementary Figure S15).

#### Systolic pressure

#### Overall

With respect to decrease on systolic pressure among enrolled participants, metabolic surgery has a significant advantage against non-surgical remedies (*P* < 0.00001) (Supplementary Figure S16).

#### Follow-up duration

Regarding the efficacy of systolic pressure reduction, patients with 2-year postoperative follow-up equaled to those receiving conventional interventions (*P* = 0.30), while bariatric surgery was a superior option among sufferers with 1-year (*P* = 0.001) and long-range follow-up (*P* = 0.02) (Supplementary Figure S16).

#### Surgical techniques

Bariatric surgery featuring sleeve gastrectomy (*P* = 0.36), laparoscopic adjustable gastric banding (*P* = 0.36) and biliopancreatic diversion (*P* = 0.90) was statistically comparable with non-surgical strategies in terms of reduction on systolic pressure, except for the dominant efficacy of Roux-en-Y gastric bypass (*P* = 0.007) (Supplementary Figure S17).

#### Levels of obesity

In comparison to conservative interventions, bariatric surgery played a preponderant and comparable role among nonseverely (*P* < 0.00001) and severely obese patients (*P* = 0.41) respectively, in terms of reduction on systolic pressure (Supplementary Figure S18).

#### Diastolic pressure

#### Overall

There was no significant difference between surgical and non-surgical strategies towards diastolic pressure reduction among included patients (*P* = 0.50) (Supplementary Figure S19).

#### Follow-up duration

Bariatric surgery had a comparable impact on attenuating diastolic pressure against conventional therapies, within patients being followed up for 1-year (*P* = 0.26) and 2-year (*P* = 0.55). However, surgical interventions were inferior to non-invasive remedies amid patients with long-term follow-up (*P* = 0.03) (Supplementary Figure S19).

#### Surgical techniques

Although Roux-en-Y gastric bypass (*P* = 0.03) were in a significantly superior status, the remaining techniques were statistically comparable to conservative regimens concerning the decrease on diastolic pressure, inclusive of sleeve gastrectomy (*P* = 0.67), laparoscopic adjustable gastric banding (*P* = 0.57) and biliopancreatic diversion (*P* = 0.52) (Supplementary Figure S20).

#### Levels of obesity

Patients undergoing both interventions exhibited comparable efficacy on diastolic pressure reduction regardless of nonsevere obesity (*P* = 0.23) and severe obesity (*P* = 0.73) (Supplementary Figure S21).

#### Triglycerides

#### Overall

More decrease on triglycerides level was obtained among surgical participants, other than those receiving conventional therapies (*P* < 0.00001) (Supplementary Figure S22).

### Follow-up duration

According to our pooled outcomes, surgical patients with 1-year (*P* < 0.0001) and 2-year (*P* < 0.0001) follow-up had a greater decline on triglycerides level than those being conventionally treated, except for the comparable efficacy among patients with long-term follow-up (*P* = 0.06) (Supplementary Figure S22).

#### Surgical techniques

In terms of reducing triglycerides level, Roux-en-Y gastric bypass (*P* = 0.0005) and laparoscopic adjustable gastric banding (*P* = 0.004) exerted a more evident influence among enrolled patients, while sleeve gastrectomy (*P* = 0.08) and biliopancreatic diversion (*P* = 0.14) was therapeutically comparable with conventional remedies (Supplementary Figure S23).

#### Levels of obesity

Patients that were surgically managed benefited from a greater loss on triglycerides level than those were non-surgically intervened, regardless of nonsevere obesity (*P* < 0.00001) and severe obesity (*P* < 0.0001) (Supplementary Figure S24).

#### Total cholesterol

#### Overall

Comparing bariatric surgery and non-surgical interventions, the magnitude of total cholesterol reduction was independent of treatment strategies among obese patients (*P* = 0.13) (Supplementary Figure S25).

#### Follow-up duration

Among obese sufferers being followed up for 1-year (*P* = 0.18) and 2-year (*P* = 0.07), there was no therapeutic difference between both interventions. However, patients with long-term follow-up achieved more reduction on total cholesterol following non-surgical managements, in contrast to bariatric surgery (*P* = 0.03) (Supplementary Figure S25).

#### Surgical techniques

Besides the preponderant role of biliopancreatic diversion (*P* = 0.004), patients receiving bariatric surgery gained comparable efficacy of total cholesterol reduction against non-surgical enrollees, including sleeve gastrectomy (*P* = 0.67), Roux-en-Y gastric bypass (*P* = 0.78) and laparoscopic adjustable gastric banding (*P* = 0.62) (Supplementary Figure S26).

#### Levels of obesity

Patients with severe obesity featured a surgical advantage in terms of total cholesterol reduction (*P* = 0.01), while nonseverely obese counterparts reported no significant difference between surgical and non-surgical interventions (*P* = 0.76) (Supplementary Figure S27).

#### High density lipoprotein

#### Overall

A favorable outcome of surgical patients was observed due to their greater increase on high density lipoprotein against those were conservatively healed (*P* < 0.00001) (Supplementary Figure S28).

#### Follow-up duration

Despite of 1-year (*P* < 0.00001), 2-year (*P* = 0.0005) and long-term follow-up (*P* = 0.001), metabolic surgery led to more increase on high density lipoprotein among included patients, compared to non-surgical recipients (Supplementary Figure S28).

#### Surgical techniques

The recipients of sleeve gastrectomy (*P* = 0.003) and Roux-en-Y gastric bypass (*P* < 0.00001) displayed a higher increase on high density lipoprotein. However, the increase on high density lipoprotein was identically obtained between non-surgical treatments and bariatric surgery of laparoscopic adjustable gastric banding (*P* = 0.19) and biliopancreatic diversion (*P* = 0.27) (Supplementary Figure S29).

#### Levels of obesity

Whether participants with nonsevere (*P* < 0.00001) or severe obesity (*P* < 0.00001), bariatric surgery was a more capable pattern of elevating high density lipoprotein than traditional modes (Supplementary Figure S30).

#### Low density lipoprotein

#### Overall

Among obese patients, both surgical and non-surgical interventions led to comparable efficacy in reduction of low density lipoprotein (*P* = 0.21) (Supplementary Figure S31).

#### Follow-up duration

Comparable to surgical patients with 1-year (*P* = 0.24) and 2-year follow-up (*P* = 0.14), recipients of conventional strategies exceled those with long-term postoperative follow-up duration, with regard to the reduction on low density lipoprotein (*P* < 0.00001) (Supplementary Figure S31).

#### Surgical techniques

Based on subgroup statistics of sleeve gastrectomy (*P* = 0.34), Roux-en-Y gastric bypass (*P* = 0.71) and laparoscopic adjustable gastric banding (*P* = 0.46), it was mathematically comparable between surgical and non-surgical patients regarding the decrease on low density lipoprotein. Nevertheless, patients undergoing biliopancreatic diversion had an advantage over those were conventionally cured (*P* < 0.00001) (Supplementary Figure S32).

#### Levels of obesity

There was no statistical significance between bariatric surgery and non-surgical intervention concerning the reduction on low density lipoprotein, according to subgroup analysis of nonseverely (*P* = 0.76) and severely obese patients (*P* = 0.09) (Supplementary Figure S33).

### Sensitivity analysis

Firstly, by interchanging random-effects and fixed-effects models, the overall as well as subgroup outcomes of primary endpoints (weight loss and diabetic remission) were confirmed to be statistically stable (Table 3).

**Table 3:** Outcomes of weight loss by sensitivity analysis

*P* value	Overall	Follow-up duration			Surgical techniques				Levels of obesity	
		1-year	2-year	Long-term	SG	RYGB	LAGB	BPD	Nonsevere	Severe
Random effects	<0.00001	<0.00001	<0.00001	<0.00001	<0.00001	<0.00001	<0.00001	<0.00001	<0.00001	<0.00001
Fixed effects	<0.00001	<0.00001	<0.00001	<0.00001	<0.00001	<0.00001	<0.00001	<0.00001	<0.00001	<0.00001
With low-quality	<0.00001	<0.00001	<0.00001	<0.00001	<0.00001	<0.00001	<0.00001	<0.00001	<0.00001	<0.00001
Without low-quality	<0.00001	<0.00001	<0.00001	<0.00001	<0.00001	<0.00001	<0.00001	<0.00001	<0.00001	<0.00001
Previous criteria	<0.00001	<0.00001	<0.00001	<0.00001	<0.00001	<0.00001	<0.00001	<0.00001	<0.00001	<0.00001
Altered criteria	<0.00001	<0.00001	<0.00001	<0.00001	<0.00001	<0.00001	<0.0001	<0.00001	<0.00001	<0.00001

SG: sleeve gastrectomy; RYGB: Roux-en-Y gastric bypass; LAGB: laparoscopic adjustable gastric banding; BPD: biliopancreatic diversion;

Secondly, by eliminating four low-quality trials of DSS, Heindorff 1997, Mingrone 2002 along with O'Brien 2013, the stability of primary endpoints were numerically verified, irrespective of overall or subgroup analysis (Table 3).

Thirdly, since O'Brien 2010 merely contained adolescent participants, the inclusion criteria had been altered by excluding it from pooling analysis. Consequently, results of primary endpoints remained stable under circumstances of novel criteria (Table 3).

Fourthly, by individually removing the eligible studies from primary endpoints, the steadiness of our meta-analysis was graphically proved with aid of STATA 12.0 (Supplementary Figure S34).

### Publication bias

Concerning the primary endpoints of weight loss (Egger: 0.002; Begg: 0.003) and diabetic remission (Egger: 0.001; Begg: 0.043), Egger's test and Begg's test consistently confirmed the presence of publication bias among the included studies (Supplementary Figure S35). However, by “Trim and Fill” method, the pooled outcomes were mathematically equivalent although 2 trials were added for weight loss (before: *P* < 0.00001; after: *P* < 0.0001) and 6 added for diabetic remission (before: *P* < 0.00001; after: *P* < 0.0001) (Supplementary Figure S36).

## DISCUSSION

Although Golomb et al had retrospectively doubted the dominant role of bariatric surgery at 5-year follow-up [38], its contrastive efficacy against non-surgical interventions remains advantageous according to our pooled analysis, especially concerning the long-term durability of weight loss and hyperglycemic remission. It is experimentally explanatory that physiological adaptation instead of mechanical reconstruction seems to mainly contribute to the prolonged impacts on energy homeostasis. Adaptive gastrointestinal remodeling, neuroendocrine hormone changes, bile acid signaling and gut microbiota adjustment play combined roles in ameliorating metabolic abnormality and sustaining postoperative energy balance [39]. Nevertheless, along with the increasing follow-up period, the cardiovascular improvement has been therapeutically offset among patients undergoing bariatric surgery, which is confirmed by the comparable outcomes of certain endpoints in our pooled analysis (systolic pressure, diastolic pressure, total cholesterol and low-density lipoprotein). Hence, distant benefits of cardiovascular disorders remain controversial following bariatric surgery, despite Gloy et al [4] had convinced surgeons with short-term advantages on cardiovascular indicators. The close interplay between obesity, diabetes and cardiovascular diseases has been extensively investigated [40]. Following improvements on metabolic status, the lowered risk of cardiovascular accidents is relatively comprehensible among patients with short-term period of follow-up. Thus the long-term insignificance on cardiovascular endpoints seems to blame on those mechanisms independent of ameliorated obesity and hyperglycemia. One possible explanation is that except for biliopancreatic diversion, the prevalent surgical styles including sleeve gastrectomy, Roux-en-Y and laparoscopic adjustable gastric banding fail to adequately impose on cholesterol homeostasis than medication of statins, which consequently culminates in angiosclerosis and hypertension. Besides, cardiovascular dysfunctions are easier to be emotionally and genetically affected, which is inappropriate to simply identify it as metabolic comorbidities [41, 42].

It is empirically regarded that compared to severe obesity, patients suffering from nonsevere obesity manifest with better general conditions which makes it more economical to be conventionally cured. However, deriving from our pooled evidence, nonseverely obese patients should rather receive bariatric surgery than conservative managements, which is similar to those featuring morbid obesity and is a vital addition to current literatures.

Adolescent obesity and overweight crowds have been targeted as further research highlights of bariatric surgery. Uncontrollable weight increase has severer impacts on pubescents than adults, simultaneously damaging their sexual development and intelligence growth [43]. O'Brien et al [23] had reported a randomized trial constituted by juveniles that laparoscopic adjustable gastric banding led to more reduction on excess weight than lifestyle intervention, as well as improved life-quality. However, more persuasive investigations are still needed to clarify its clinical worthiness on teenager obesity. Additionally, a substantial proportion of diabetic patients are just overweight instead of clinically obese. But the actual role of bariatric surgery among such populations remains undetermined. By a randomized research, Wentworth et al [44] firstly described a favorable effect of glycemic control and weight loss among overweight patients undergoing laparoscopic adjustable gastric banding in contrast to those with non-surgical treatments. This probably hints that bariatric surgery may fit for all crowds with weight redundancy despite long-term and pooled evidences are scarce.

Notwithstanding the statistical outcomes are of great strength in both methodology and statistics, there are still some limitations within. First of all, the internal heterogeneity is still in a considerable level despite the subgroup analyses have been additionally performed. Difference from chemical examination methods, surgical manipulation techniques and diversified non-surgical interventions (life-style and medications) may contribute to inerasably internal heterogeneity. More accurate grouping on different confounding factors may benefit the source seeking of heterogeneity. Secondly, raw data of life-quality and survival prognostication are inadequate for meta-analyzing, making it less valuable in terms of comparisons on life expectancy. Moreover, mutual comparison of financial burdens is still lacking, blockading a more comprehensive appraisal of both strategies.

Taken together, despite of the shortcomings of surgical therapeutics (a forced alteration of diet habits; large load of exercise required; 8% of revision rate) [45], bariatric surgery is a more efficient technique for ameliorating obesity and its relevant comorbidities, in contrast to non-surgical interventions. However, more rigorously designed trials with long-term follow-up duration are still required for future supplements and updates.

## MATERIALS AND METHODS

In line with the PRISMA Checklist [46] and Cochrane Collaboration protocols [47], the pooled analysis was designed and implemented in a standard manner. The entire procedures were independently performed by two investigators. Any discrepancy was resolved by mutual discussion.

### Search strategy

In order to guarantee the integrity of literature retrieval, databases of PubMed, Web of Science, EMBASE and Cochrane Library were electronically searched with a search term “bariatric randomized OR bariatric randomised OR obesity surgery”. Both abstracts and full-texts were elaborately examined for fear of omission or ineligible inclusion. Additionally, citation lists of formerly published meta-analysis were screened as well.

### Study selection

Studies were eligibly included because of the following criteria: 1. English-written and officially published trials until December 2015; 2. Randomized controlled trials evaluating the comparative efficacy of surgical *versus* non-surgical interventions among obese patients (body mass index > 30); 3. Adequate raw data of interested endpoints including metabolic and cardiovascular indicators;

Studies were eventually eliminated due to the following reasons: 1. Duplicated publications; 2. Inadequate sample-size (< 10); 3. Lack of follow-up materials;

### Data extraction

With regard to baseline, primary (Weight loss; Remission of type 2 diabetes mellitus) and secondary parameters (Excessive weight loss; Fasting glucose; Glycated hemoglobin; Waist circumference; Systolic pressure; Diastolic pressure; Triglycerides; Total cholesterol; High density lipoprotein; Low density lipoprotein), a well-prepared electronic form was designed to facilitate date extraction from tables, figures, text contents and supplementary information within the eligible trials. Overlapped data deriving from a single registered trial was mathematically combined to prevent repetitive counting. All continuous variables were rounded to one decimal place.

### Methodological assessment

Firstly, a Revised Jadad's Scale [48] was employed in order for a rigorous appraisal of the study design. A total of four categories consisted of the scale, including randomization, allocation concealment, blindness and withdrawal, with a maximum score of seven. Studies graded with four points or more were identified as high-quality trials (Table 4).

**Table 4:** Revised Jadad's Scale

**Random sequence production**
a. Adequate (computer generated random numbers or similar methods) (2 points)
b. Unclear (a randomized trial but without description of randomization methods) (1 point)
c. Inadequate (an alternative allocation without randomization) (0 point)
**Allocation concealment**
a. Adequate (a central institution-controlled allocation) (2 points)
b. Unclear (random numerical table or other similar methods) (1 point)
c. Inadequate (alternative allocation without adequate concealment) (0 point)
**Blindness**
a. Adequate (comparable placebo or similar methods) (2 points)
b. Unclear (a blind trial without details statement) (1 point)
c. Inadequate (inappropriate blind methods or non-blind trials) (0 point)
**Withdrawal**
a. Description (a detailed statement about the numbers and reasons of withdrawals) (1 point)
b. No description (no statement about the numbers and reasons of withdrawals) (0 point)

Secondly, as an addition to Revised Jadad's Scale, Review Manager 5.3 assisted us to summarize the risk of bias amid the qualified literatures. The symbol of green, yellow and red represented low risk of bias, unclear risk of bias and high risk of bias respectively, in terms of seven constituted categories (random sequence generation; allocation concealment; blinding of participants and personnel; blinding of outcome assessment; incomplete outcome data; selective reporting; other bias). The higher proportion that green occupies, the lower risk of bias there is [47].

### Statistical methods

Review Manager 5.3 was employed as a statistical platform for our quantitative analysis. The effect-sizes of dichotomous and continuous variable were calculated by models of odds ratio and weighted mean difference respectively, along with 95% confidence interval. If the source data on endpoints were not offered explicitly, median was statistically regarded as mean while standard deviation was derived from range, interquartile range or 95% confidence interval as appropriate [47, 49]. A demand-based merging of subgroups was rigorously conducted to enable sole pairwise comparisons. If necessary, numeric change from baseline values was computed in accord with the statistical instructions of Cochrane Handbook [47]. The overall statistical heterogeneity was quantified by the degree of inconsistency (I^2^) [50]. Revealing a substantially lower heterogeneity, the fixed-effects model was recommended in the setting of I^2^ < 25%. Otherwise a random-effects model was preferred under the remaining circumstances [51], in order for adjustment of potential variations across the retrieved studies. For the sake of detecting internal stability, sensitivity analysis was accomplished *via* eliminating low-quality trials, exclusion of controversial studies and comparing the outcome variation between fixed-effects and random-effects models. Moreover, the incorporated outcomes were additionally classified into various subgroups for purpose of more specific and instructive discoveries. With aid of STATA 12.0, publication bias was numerically examined by Begg's test and Egger's test [52]. A “Trim and Fill” method was conducted in the setting of significant publication bias, in order for adding inadequate studies as well as examining the stability of outcomes. Significant difference was denoted as *P* < 0.05.

## SUPPLEMENTARY MATERIAL


