# Supplementary material for: The comprehensive summary of surgical versus non-surgical treatment for obesity: a systematic review and meta-analysis of randomized controlled trials
Source: Oncotarget. 2016 May 24;7(26):39216–30. doi: 10.18632/oncotarget.9581 (PMC5129927; doi:10.18632/oncotarget.9581)
Supplement: Supplementary file 1 [file oncotarget-07-39216-s001.pdf]

The comprehensive summary of surgical versus non-surgical treatment for obesity: a systematic review and meta-analysis of randomized controlled trials

Supplementary Material

Study or Subgroup	Surgery Events	Surgery Total	Control Events	Control Total	Weight	Odds Ratio M-H, Random, 95% CI
**1.2.1 1-year**						
DSS 2013	43	60	18	60	14.6%	5.90 [2.69, 12.97]
Liang 2013	28	31	0	70	7.1%	1148.14 [57.45, 22945.01]
Parikh 2014	13	20	0	24	7.2%	88.20 [4.67, 1666.35]
SLIMM-T2D 2014/15	17	37	8	41	13.9%	3.51 [1.28, 9.60]
STAMPEDE 2012	56	99	0	41	7.6%	107.80 [6.45, 1802.02]
TRIABETES 2014	18	46	0	23	7.4%	30.51 [1.74, 533.59]
**Subtotal (95% CI)**		**293**		**259**	**57.8%**	**28.25 [5.80, 137.68]**
Total events	175		26			
Heterogeneity: Tau² = 2.63; Chi² = 24.16, df = 5 (P = 0.0002); I² = 79%						
Test for overall effect: Z = 4.13 (P < 0.0001)						
**1.2.2 2-year**						
DIBASY 2012	34	38	0	18	7.1%	283.67 [14.47, 5561.54]
Dixon 2008	22	30	4	30	12.8%	17.88 [4.74, 67.43]
**Subtotal (95% CI)**		**68**		**48**	**19.9%**	**51.87 [3.46, 776.95]**
Total events	56		4			
Heterogeneity: Tau² = 2.64; Chi² = 2.91, df = 1 (P = 0.09); I² = 66%						
Test for overall effect: Z = 2.86 (P = 0.004)						
**1.2.3 3-year or more**						
DIBASY 2015	19	38	0	15	7.4%	31.00 [1.73, 555.16]
STAMPEDE 2014	44	97	0	40	7.6%	67.37 [4.03, 1127.02]
TRIABETES 2015	14	38	0	14	7.3%	17.16 [0.95, 309.76]
**Subtotal (95% CI)**		**173**		**69**	**22.3%**	**33.39 [6.39, 174.54]**
Total events	77		0			
Heterogeneity: Tau² = 0.00; Chi² = 0.46, df = 2 (P = 0.79); I² = 0%						
Test for overall effect: Z = 4.16 (P < 0.0001)						
**Total (95% CI)**		**534**		**376**	**100.0%**	**30.40 [10.43, 88.58]**
Total events	308		30			
Heterogeneity: Tau² = 1.87; Chi² = 32.65, df = 10 (P = 0.0003); I² = 69%						
Test for overall effect: Z = 6.26 (P < 0.00001)						
Test for subgroup differences: Chi² = 0.14, df = 2 (P = 0.93), I² = 0%						

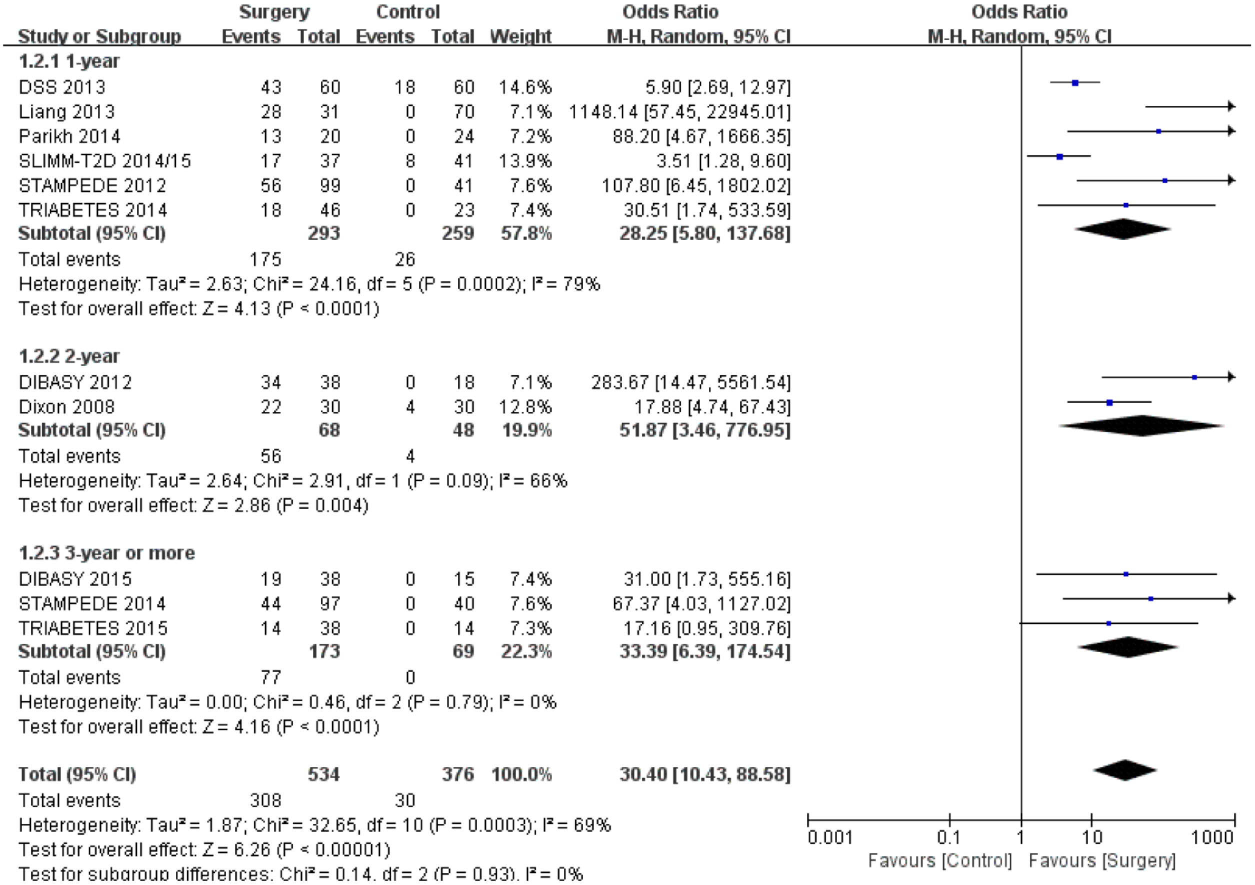

Figure S1.The forest plot of diabetic remission in terms of follow-up duration

Study or Subgroup	Surgery Events	Surgery Total	Control Events	Control Total	Weight	Odds Ratio M-H, Random, 95% CI
**2.2.1 Sleeve gastrectomy**						
Parikh 2014	10	11	0	24	3.8%	343.00 [12.89, 9126.80]
STAMPEDE 2012	22	49	0	41	4.5%	67.91 [3.95, 1166.48]
STAMPEDE 2014	16	49	0	40	4.5%	39.90 [2.31, 690.04]
**Subtotal (95% CI)**		**109**		**105**	**12.9%**	**87.21 [15.68, 485.06]**
Total events	48		0			
Heterogeneity: Tau² = 0.00; Chi² = 1.06, df = 2 (P = 0.59); I² = 0%						
Test for overall effect: Z = 5.10 (P < 0.00001)						
**2.2.2 Roux-en-Y gastric bypass**						
DIBASY 2012	15	19	0	18	4.3%	127.44 [6.35, 2556.40]
DIBASY 2015	7	19	0	15	4.3%	18.60 [0.97, 358.26]
DSS 2013	43	60	18	60	9.4%	5.90 [2.69, 12.97]
Liang 2013	28	31	0	70	4.3%	1148.14 [57.45, 22945.01]
Parikh 2014	2	6	0	24	3.9%	27.22 [1.11, 666.78]
SLIMM-T2D 2014	11	19	3	19	7.5%	7.33 [1.58, 33.97]
STAMPEDE 2012	34	50	0	41	4.5%	173.55 [10.04, 2998.71]
STAMPEDE 2014	28	48	0	40	4.5%	112.61 [6.54, 1939.08]
TRIABETES 2014	12	24	0	23	4.4%	47.00 [2.56, 861.72]
TRIABETES 2015	8	18	0	14	4.3%	23.48 [1.22, 453.35]
**Subtotal (95% CI)**		**294**		**324**	**51.6%**	**36.89 [11.13, 122.25]**
Total events	188		21			
Heterogeneity: Tau² = 2.05; Chi² = 24.52, df = 9 (P = 0.004); I² = 63%						
Test for overall effect: Z = 5.90 (P < 0.00001)						
**2.2.3 Laparoscopic adjustable gastric banding**						
Dixon 2008	22	30	4	30	8.1%	17.88 [4.74, 67.43]
Parikh 2014	1	3	0	24	3.6%	29.40 [0.93, 930.79]
SLIMM-T2D 2015	6	18	5	22	7.9%	1.70 [0.42, 6.88]
TRIABETES 2014	6	22	0	23	4.4%	18.52 [0.97, 351.82]
TRIABETES 2015	6	20	0	14	4.3%	13.00 [0.67, 252.66]
**Subtotal (95% CI)**		**93**		**113**	**28.2%**	**8.79 [2.48, 31.14]**
Total events	41		9			
Heterogeneity: Tau² = 0.84; Chi² = 7.07, df = 4 (P = 0.13); I² = 43%						
Test for overall effect: Z = 3.37 (P = 0.0008)						
**2.2.4 Biliopancreatic diversion**						
DIBASY 2012	19	19	0	18	3.0%	1443.00 [27.20, 76548.96]
DIBASY 2015	12	19	0	15	4.3%	51.67 [2.68, 995.15]
**Subtotal (95% CI)**		**38**		**33**	**7.3%**	**208.86 [8.03, 5435.85]**
Total events	31		0			
Heterogeneity: Tau² = 2.49; Chi² = 1.78, df = 1 (P = 0.18); I² = 44%						
Test for overall effect: Z = 3.21 (P = 0.001)						
**Total (95% CI)**		**534**		**575**	**100.0%**	**31.13 [13.87, 69.84]**
Total events	308		30			
Heterogeneity: Tau² = 1.64; Chi² = 45.06, df = 19 (P = 0.0007); I² = 58%						
Test for overall effect: Z = 8.34 (P < 0.00001)						
Test for subgroup differences: Chi² = 6.57, df = 3 (P = 0.09), I² = 54.3%						

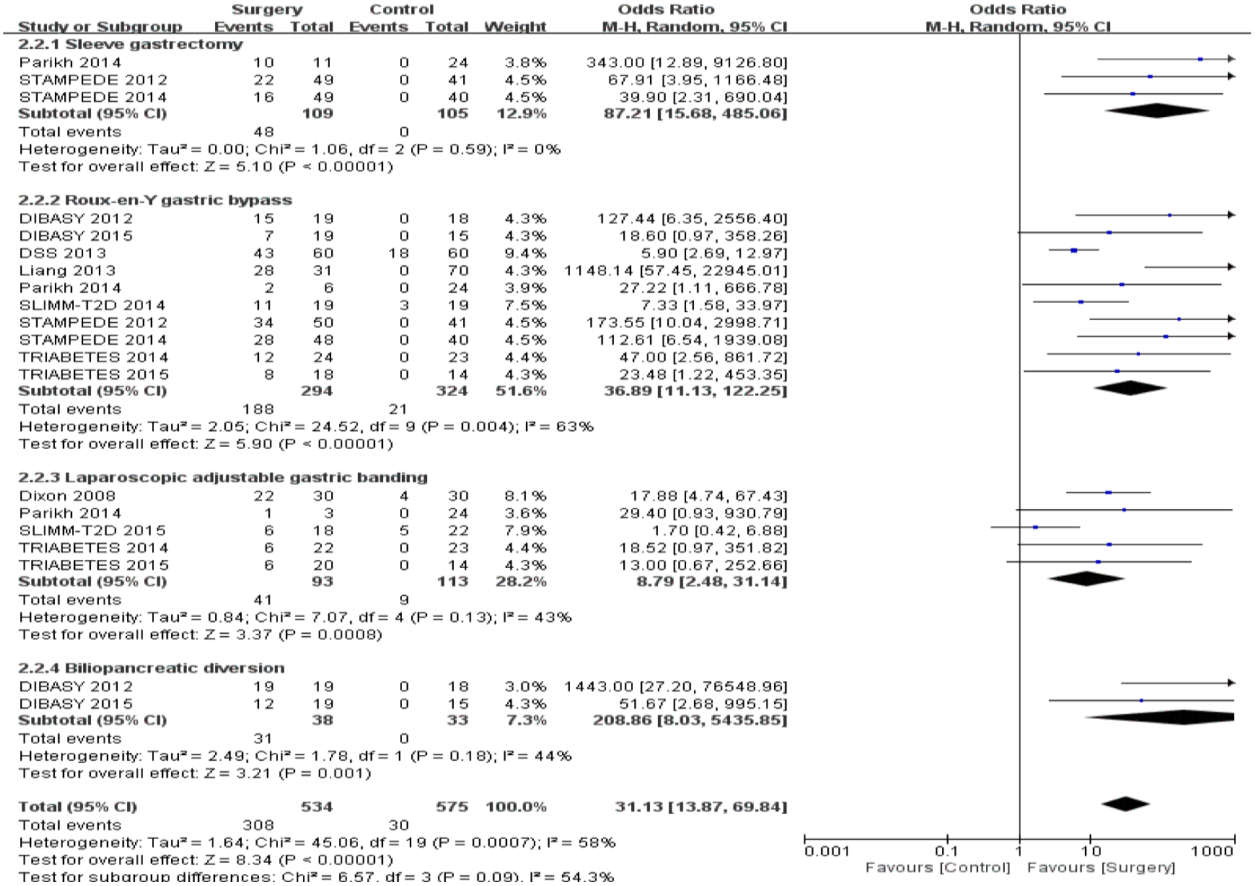

Figure S2.The forest plot of diabetic remission in terms of surgical techniques

Study or Subgroup	Surgery Events	Surgery Total	Control Events	Control Total	Weight	Odds Ratio M-H, Random, 95% CI
**3.2.1 Nonsevere obesity**						
DSS 2013	43	60	18	60	14.6%	5.90 [2.69, 12.97]
Liang 2013	28	31	0	70	7.1%	1148.14 [57.45, 22945.01]
Parikh 2014	13	20	0	24	7.2%	88.20 [4.67, 1666.35]
TRIABETES 2014	18	46	0	23	7.4%	30.51 [1.74, 533.59]
TRIABETES 2015	14	38	0	14	7.3%	17.16 [0.95, 309.76]
**Subtotal (95% CI)**		**195**		**191**	**43.7%**	**40.38 [5.19, 313.99]**
Total events	116		18			
Heterogeneity: Tau² = 3.82; Chi² = 15.82, df = 4 (P = 0.003); I² = 75%						
Test for overall effect: Z = 3.53 (P = 0.0004)						
**3.2.2 Severe obesity**						
DIBASY 2012	34	38	0	18	7.1%	283.67 [14.47, 5561.54]
DIBASY 2015	19	38	0	15	7.4%	31.00 [1.73, 555.16]
Dixon 2008	22	30	4	30	12.8%	17.88 [4.74, 67.43]
SLIMM-T2D 2014/15	17	37	8	41	13.9%	3.51 [1.28, 9.60]
STAMPEDE 2012	56	99	0	41	7.6%	107.80 [6.45, 1802.02]
STAMPEDE 2014	44	97	0	40	7.6%	67.37 [4.03, 1127.02]
**Subtotal (95% CI)**		**339**		**185**	**56.3%**	**28.84 [6.39, 130.06]**
Total events	192		12			
Heterogeneity: Tau² = 2.22; Chi² = 16.71, df = 5 (P = 0.005); I² = 70%						
Test for overall effect: Z = 4.37 (P < 0.0001)						
**Total (95% CI)**		**534**		**376**	**100.0%**	**30.40 [10.43, 88.58]**
Total events	308		30			
Heterogeneity: Tau² = 1.87; Chi² = 32.65, df = 10 (P = 0.0003); I² = 69%						
Test for overall effect: Z = 6.26 (P < 0.00001)						
Test for subgroup differences: Chi² = 0.07, df = 1 (P = 0.80), I² = 0%						

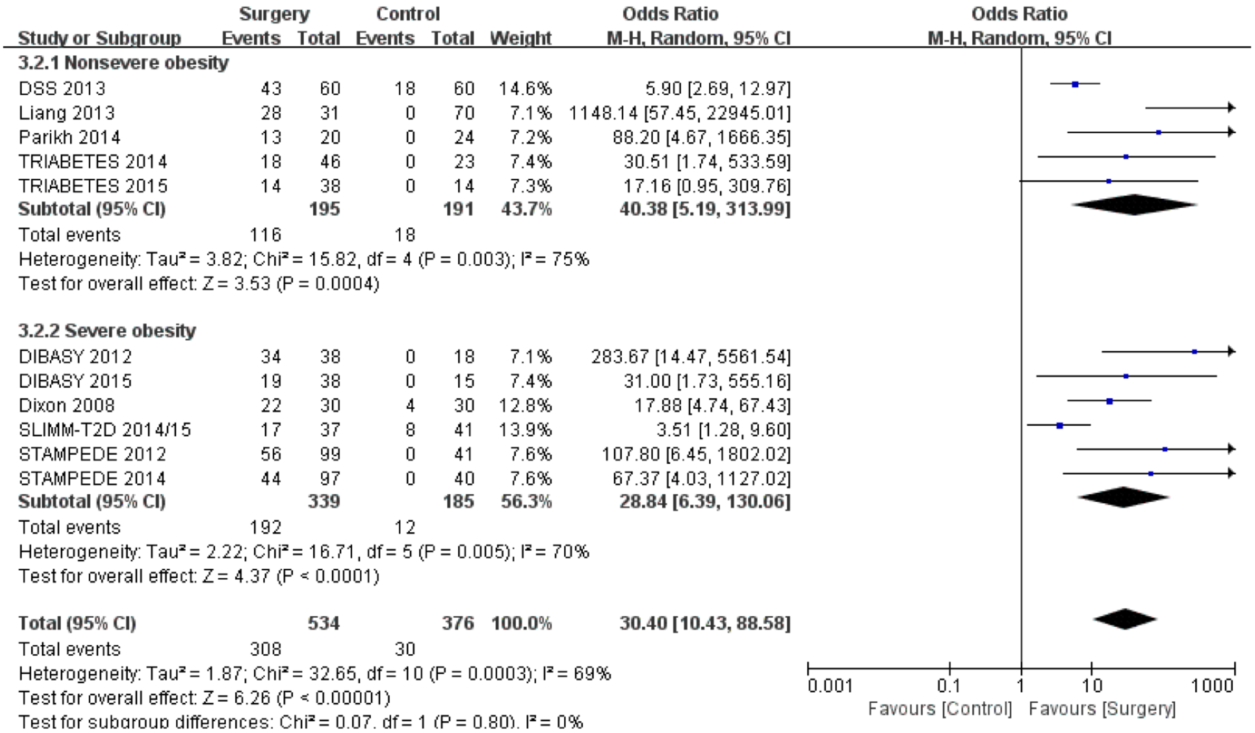

Figure S3.The forest plot of diabetic remission in terms of obesity levels

Study or Subgroup	Surgery Mean	Surgery SD	Surgery Total	Control Mean	Control SD	Control Total	Weight	Mean Difference IV, Random, 95% CI
**1.3.1 1-year**								
O'Brien 2006	78.6	14	39	41.1	14.6	31	13.9%	37.50 [30.74, 44.26]
Parikh 2014	60	21.1	20	7.4	12.6	24	12.1%	52.60 [42.07, 63.13]
STAMPEDE 2012	84.5	22.8	99	13	16.4	41	13.9%	71.50 [64.76, 78.24]
**Subtotal (95% CI)**			**158**			**96**	**39.9%**	**53.89 [31.68, 76.09]**
Heterogeneity: Tau² = 367.71; Chi² = 48.86, df = 2 (P < 0.00001); I² = 96%								
Test for overall effect: Z = 4.76 (P < 0.00001)								
**1.3.2 2-year**								
DIBASY 2012	68.7	15.2	38	9.3	12.9	18	13.5%	59.40 [51.73, 67.07]
Dixon 2008	62.5	9.4	30	4.3	4.9	30	15.0%	58.20 [54.41, 61.99]
O'Brien 2006	87.2	14	39	21.8	14.6	31	13.9%	65.40 [58.64, 72.16]
O'Brien 2010	78.8	18.1	24	13.2	13.6	18	12.6%	65.60 [56.01, 75.19]
**Subtotal (95% CI)**			**131**			**97**	**55.0%**	**61.14 [57.16, 65.12]**
Heterogeneity: Tau² = 5.80; Chi² = 4.59, df = 3 (P = 0.20); I² = 35%								
Test for overall effect: Z = 30.10 (P < 0.00001)								
**1.3.3 3-year or more**								
O'Brien 2013	63	41	31	22.8	55.9	20	5.1%	40.20 [11.77, 68.63]
**Subtotal (95% CI)**			**31**			**20**	**5.1%**	**40.20 [11.77, 68.63]**
Heterogeneity: Not applicable								
Test for overall effect: Z = 2.77 (P = 0.006)								
**Total (95% CI)**			**320**			**213**	**100.0%**	**57.67 [49.83, 65.51]**
Heterogeneity: Tau² = 103.29; Chi² = 60.33, df = 7 (P < 0.00001); I² = 88%								
Test for overall effect: Z = 14.41 (P < 0.00001)								
Test for subgroup differences: Chi² = 2.40, df = 2 (P = 0.30), I² = 16.6%								

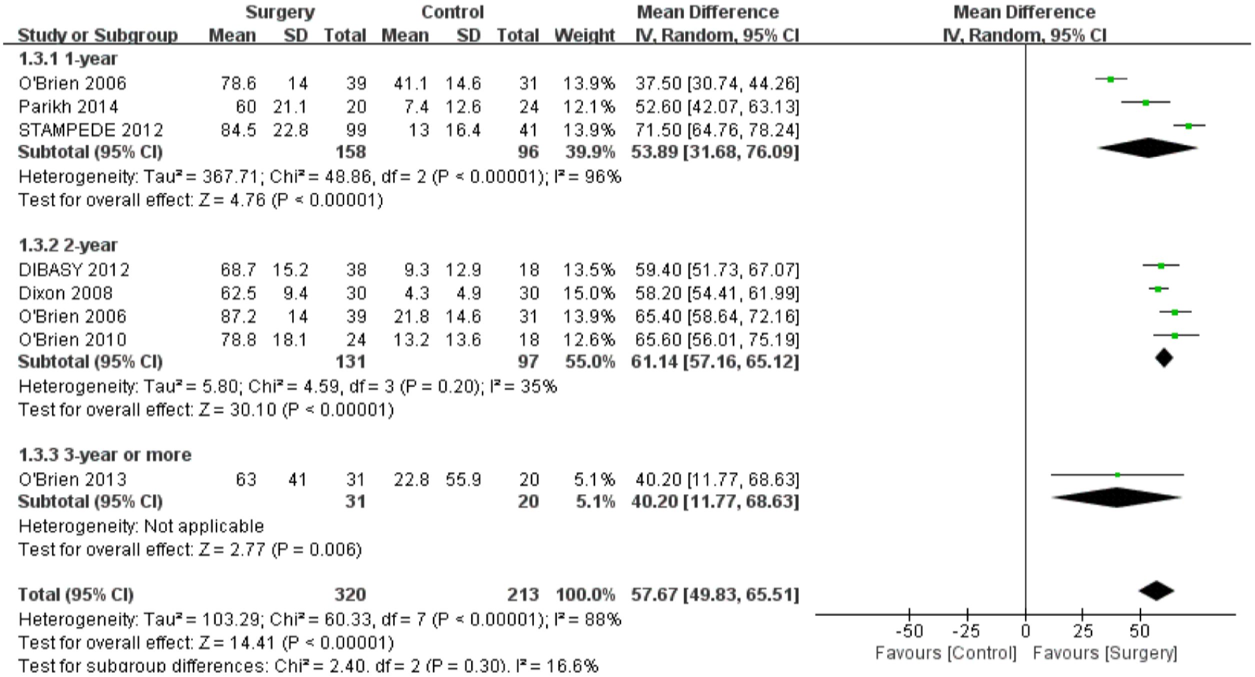

Figure S4.The forest plot of excessive weight loss (%) in terms of follow-up duration

Study or Subgroup	Surgery Mean	Surgery SD	Surgery Total	Control Mean	Control SD	Control Total	Weight	Mean Difference IV, Random, 95% CI
**2.3.1 Sleeve gastrectomy**								
STAMPEDE 2012	81	23.7	49	13	8	41	14.8%	68.00 [60.93, 75.07]
**Subtotal (95% CI)**			**49**			**41**	**14.8%**	**68.00 [60.93, 75.07]**
Heterogeneity: Not applicable								
Test for overall effect: Z = 18.84 (P < 0.00001)								
**2.3.2 Roux-en-Y gastric bypass**								
DIBASY 2012	68.1	12.7	19	9.3	12.9	18	13.4%	58.80 [50.55, 67.05]
STAMPEDE 2012	88	21.5	50	13	16.4	41	14.0%	75.00 [67.21, 82.79]
**Subtotal (95% CI)**			**69**			**59**	**27.4%**	**66.96 [51.08, 82.83]**
Heterogeneity: $Tau^2 = 114.45$; $Chi^2 = 7.82$, df = 1 (P = 0.005); $I^2 = 87\%$								
Test for overall effect: Z = 8.27 (P < 0.00001)								
**2.3.3 Laparoscopic adjustable gastric banding**								
Dixon 2008	62.5	9.4	30	4.3	4.9	30	18.8%	58.20 [54.41, 61.99]
O'Brien 2006	87.2	14	39	21.8	14.6	31	15.2%	65.40 [58.64, 72.16]
O'Brien 2010	78.8	18.1	24	13.2	13.6	18	11.9%	65.60 [56.01, 75.19]
O'Brien 2013	63	41	31	22.8	55.9	20	2.7%	40.20 [11.77, 68.63]
**Subtotal (95% CI)**			**124**			**99**	**48.6%**	**61.34 [55.41, 67.28]**
Heterogeneity: $Tau^2 = 17.63$; $Chi^2 = 6.46$, df = 3 (P = 0.09); $I^2 = 54\%$								
Test for overall effect: Z = 20.26 (P < 0.00001)								
**2.3.4 Biliopancreatic diversion**								
DIBASY 2012	69.4	17.6	19	9.3	20.9	18	9.1%	60.10 [47.62, 72.58]
**Subtotal (95% CI)**			**19**			**18**	**9.1%**	**60.10 [47.62, 72.58]**
Heterogeneity: Not applicable								
Test for overall effect: Z = 9.44 (P < 0.00001)								
**Total (95% CI)**			**261**			**217**	**100.0%**	**63.74 [58.73, 68.76]**
Heterogeneity: $Tau^2 = 31.10$; $Chi^2 = 21.52$, df = 7 (P = 0.003); $I^2 = 67\%$								
Test for overall effect: Z = 24.91 (P < 0.00001)								
Test for subgroup differences: $Chi^2 = 2.50$, df = 3 (P = 0.48), $I^2 = 0\%$								

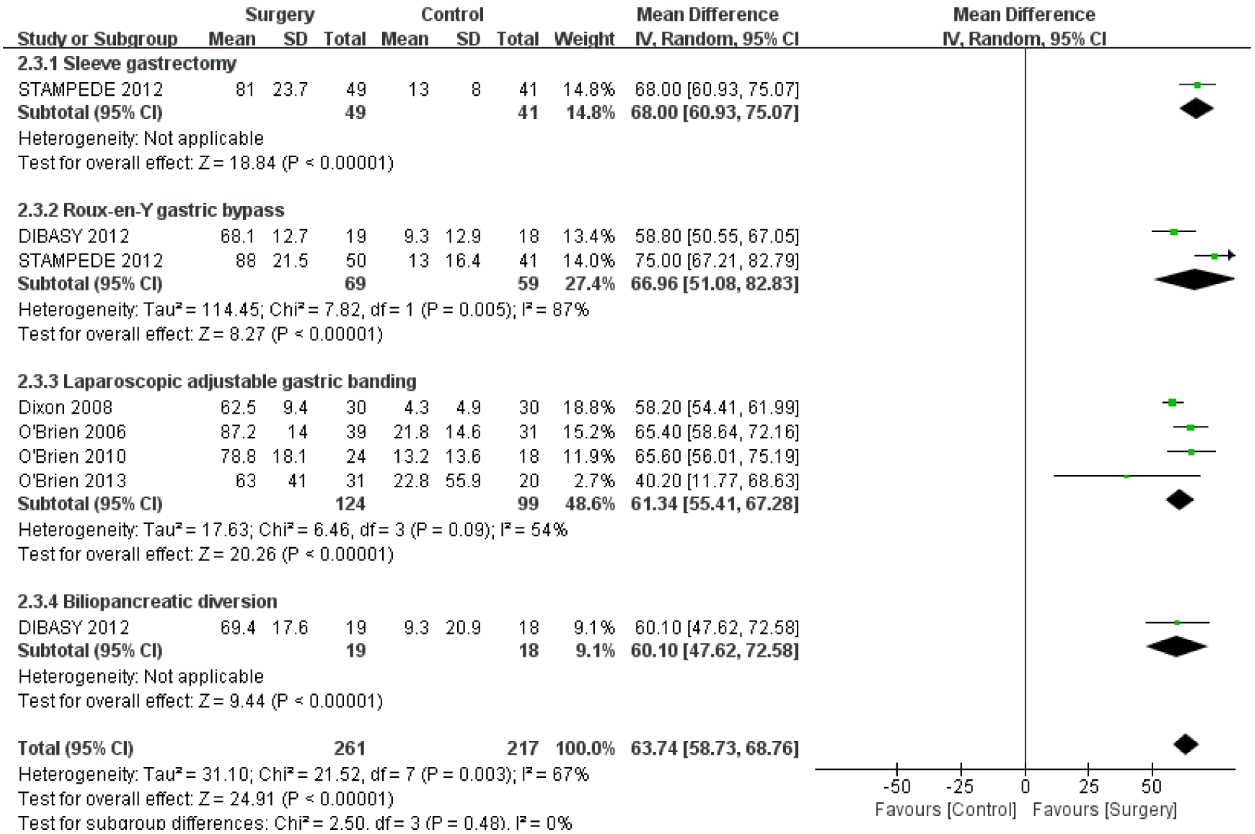

Figure S5.The forest plot of excessive weight loss (%) in terms of surgical techniques

Study or Subgroup	Surgery Mean	Surgery SD	Surgery Total	Control Mean	Control SD	Control Total	Weight	Mean Difference IV, Random, 95% CI
**3.3.1 Nonsevere obesity**								
O'Brien 2006	87.2	14	39	21.8	14.6	31	17.2%	65.40 [58.64, 72.16]
O'Brien 2013	63	41	31	22.8	55.9	20	3.0%	40.20 [11.77, 68.63]
Parikh 2014	60	21.1	20	7.4	12.6	24	12.2%	52.60 [42.07, 63.13]
**Subtotal (95% CI)**			**90**			**75**	**32.3%**	**56.85 [44.47, 69.23]**
Heterogeneity: Tau² = 73.23; Chi² = 6.12, df = 2 (P = 0.05); I² = 67%								
Test for overall effect: Z = 9.00 (P < 0.00001)								
**3.3.2 Severe obesity**								
DIBASY 2012	68.7	15.2	38	9.3	12.9	18	15.8%	59.40 [51.73, 67.07]
Dixon 2008	62.5	9.4	30	4.3	4.9	30	21.3%	58.20 [54.41, 61.99]
O'Brien 2010	78.8	18.1	24	13.2	13.6	18	13.3%	65.60 [56.01, 75.19]
STAMPEDE 2012	84.5	22.8	99	13	16.4	41	17.2%	71.50 [64.76, 78.24]
**Subtotal (95% CI)**			**191**			**107**	**67.7%**	**63.35 [56.66, 70.05]**
Heterogeneity: Tau² = 34.10; Chi² = 12.36, df = 3 (P = 0.006); I² = 76%								
Test for overall effect: Z = 18.54 (P < 0.00001)								
**Total (95% CI)**			**281**			**182**	**100.0%**	**61.68 [56.45, 66.91]**
Heterogeneity: Tau² = 29.64; Chi² = 18.53, df = 6 (P = 0.005); I² = 68%								
Test for overall effect: Z = 23.11 (P < 0.00001)								
Test for subgroup differences: Chi² = 0.82, df = 1 (P = 0.37), I² = 0%								

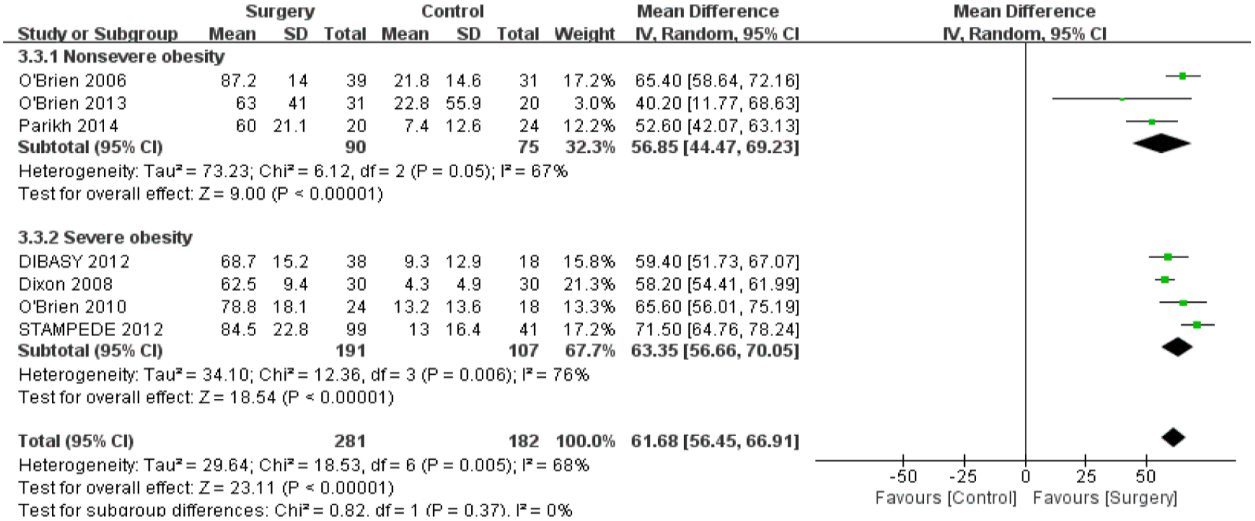

Figure S6.The forest plot of excessive weight loss (%) in terms of obesity levels

Study or Subgroup	Surgery Mean	Surgery SD	Surgery Total	Control Mean	Control SD	Control Total	Weight	Mean Difference IV, Random, 95% CI
**1.4.1 1-year**								
DSS 2013	-6.1	1.4	60	-3	1.3	60	8.2%	-3.10 [-3.58, -2.62]
Mingrone 2002	-0.9	0.5	46	-0.6	0.4	33	8.7%	-0.30 [-0.50, -0.10]
Parikh 2014	-2.8	2.5	20	0.7	4.3	24	3.9%	-3.50 [-5.54, -1.46]
SLIMM-T2D 2014/15	-2.7	0.9	37	-1.1	1.3	41	8.2%	-1.60 [-2.09, -1.11]
STAMPEDE 2012	-4.2	3.7	99	-1.6	2.8	41	6.3%	-2.60 [-3.73, -1.47]
TRIABETES 2014	-3.7	2.6	46	-0.4	2.8	23	5.6%	-3.30 [-4.67, -1.93]
**Subtotal (95% CI)**			**308**			**222**	**40.8%**	**-2.30 [-3.60, -1.01]**
Heterogeneity: Tau² = 2.33; Chi² = 149.14, df = 5 (P < 0.00001); I² = 97%								
Test for overall effect: Z = 3.48 (P = 0.0005)								
**1.4.2 2-year**								
DIBASY 2012	-4.8	3.3	38	-2.1	3	18	4.6%	-2.70 [-4.44, -0.96]
Dixon 2008	-2.8	2.1	30	-1	2.3	30	6.4%	-1.80 [-2.91, -0.69]
Dixon 2012	0.1	1.1	30	0.6	0.9	30	8.1%	-0.50 [-1.01, 0.01]
O'Brien 2006	-0.4	0.8	39	0	0.4	31	8.5%	-0.40 [-0.69, -0.11]
O'Brien 2010	-0.4	1.1	24	0.2	0.5	18	8.1%	-0.60 [-1.10, -0.10]
TRAMOMTANA 2015	-0.6	1	23	-0.1	1.6	47	7.9%	-0.50 [-1.11, 0.11]
**Subtotal (95% CI)**			**184**			**174**	**43.6%**	**-0.69 [-1.07, -0.32]**
Heterogeneity: Tau² = 0.11; Chi² = 11.85, df = 5 (P = 0.04); I² = 58%								
Test for overall effect: Z = 3.61 (P = 0.0003)								
**1.4.3 3-year or more**								
DIBASY 2015	-4.6	3.2	38	-4.1	4.1	15	3.3%	-0.50 [-2.81, 1.81]
STAMPEDE 2014	-3.6	5.4	97	-0.3	5.1	40	4.1%	-3.30 [-5.21, -1.39]
TRIABETES 2015	-2.8	1	38	-1.6	0.7	14	8.2%	-1.20 [-1.69, -0.71]
**Subtotal (95% CI)**			**173**			**69**	**15.7%**	**-1.61 [-2.97, -0.25]**
Heterogeneity: Tau² = 0.86; Chi² = 4.83, df = 2 (P = 0.09); I² = 59%								
Test for overall effect: Z = 2.32 (P = 0.02)								
**Total (95% CI)**			**665**			**465**	**100.0%**	**-1.55 [-2.08, -1.01]**
Heterogeneity: Tau² = 0.85; Chi² = 178.01, df = 14 (P < 0.00001); I² = 92%								
Test for overall effect: Z = 5.65 (P < 0.00001)								
Test for subgroup differences: Chi² = 6.65, df = 2 (P = 0.04), I² = 69.9%								

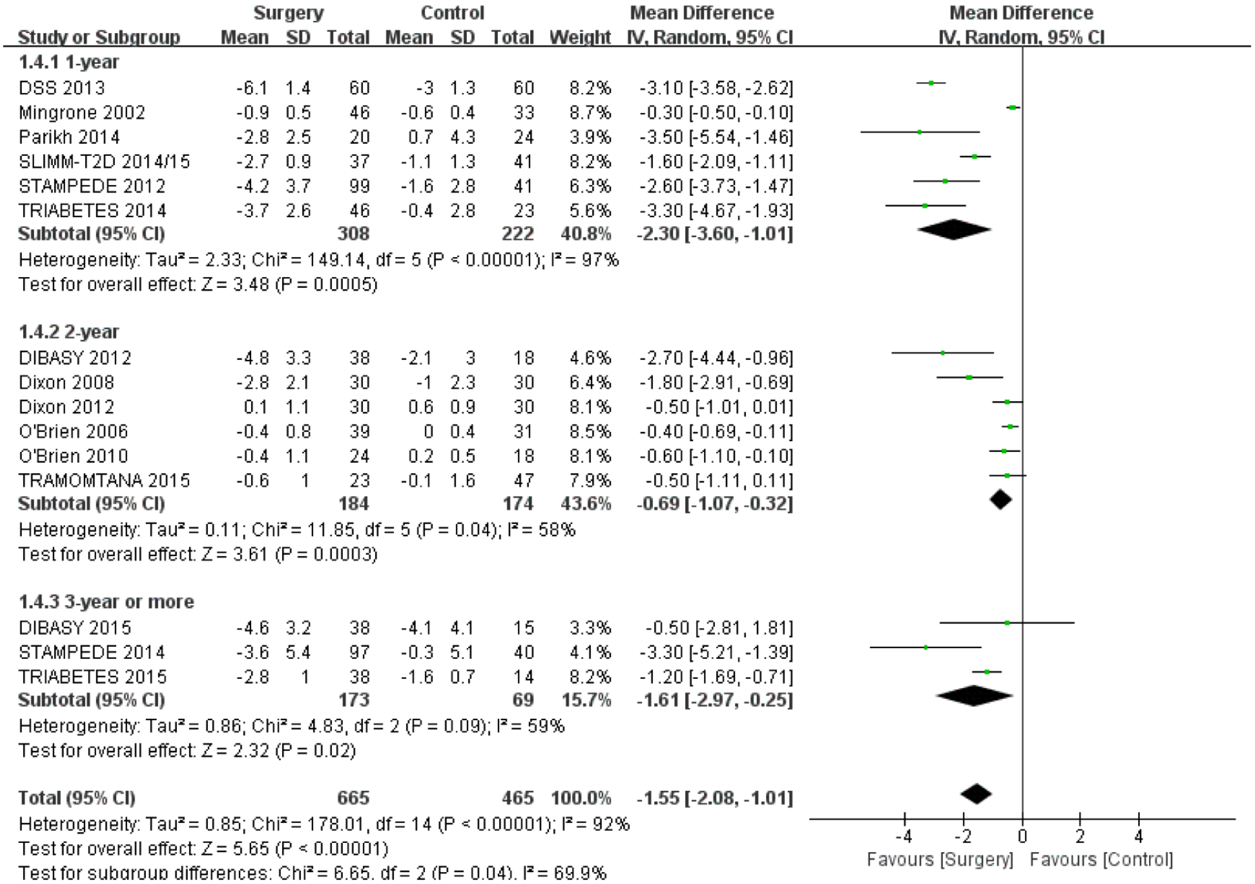

Figure S7.The forest plot of fasting glucose (mmol/L) in terms of follow-up duration

Study or Subgroup	Surgery Mean	Surgery SD	Surgery Total	Control Mean	Control SD	Control Total	Weight	Mean Difference IV, Random, 95% CI
**2.4.1 Sleeve gastrectomy**								
Parikh 2014	-3.9	2.6	11	0.7	4.3	24	2.5%	-4.60 [-6.91, -2.29]
STAMPEDE 2012	-3.5	3.3	49	-1.6	2.8	41	4.2%	-1.90 [-3.16, -0.64]
STAMPEDE 2014	-2.6	5.1	49	-0.3	5.1	40	2.7%	-2.30 [-4.43, -0.17]
**Subtotal (95% CI)**			**109**			**105**	**9.4%**	**-2.72 [-4.26, -1.19]**
Heterogeneity: Tau² = 0.94; Chi² = 4.09, df = 2 (P = 0.13); I² = 51%								
Test for overall effect: Z = 3.48 (P = 0.0005)								
**2.4.2 Roux-en-Y gastric bypass**								
DIBASY 2012	-3.9	3.2	19	-2.1	3	18	2.9%	-1.80 [-3.80, 0.20]
DIBASY 2015	-4.3	3.1	19	-4.1	4.1	15	2.2%	-0.20 [-2.70, 2.30]
DSS 2013	-6.1	1.4	60	-3	1.3	60	5.7%	-3.10 [-3.58, -2.62]
Parikh 2014	-1.4	2	6	0.7	4.3	24	2.4%	-2.10 [-4.45, 0.25]
SLIMM-T2D 2014	-2.7	1.2	19	0	1.1	19	5.3%	-2.70 [-3.43, -1.97]
STAMPEDE 2012	-4.8	4	50	-1.6	2.8	41	3.9%	-3.20 [-4.60, -1.80]
STAMPEDE 2014	-4.8	5.6	48	-0.3	5.1	40	2.6%	-4.50 [-6.74, -2.26]
TRIABETES 2014	-4.4	2.5	24	-0.4	2.8	23	3.7%	-4.00 [-5.52, -2.48]
TRIABETES 2015	-3.7	0.6	18	-1.6	0.7	14	5.7%	-2.10 [-2.56, -1.64]
**Subtotal (95% CI)**			**263**			**254**	**34.4%**	**-2.72 [-3.29, -2.15]**
Heterogeneity: Tau² = 0.34; Chi² = 19.69, df = 8 (P = 0.01); I² = 59%								
Test for overall effect: Z = 9.37 (P < 0.00001)								
**2.4.3 Laparoscopic adjustable gastric banding**								
Dixon 2008	-2.8	2.1	30	-1	2.3	30	4.5%	-1.80 [-2.91, -0.69]
Dixon 2012	0.1	1.1	30	0.6	0.9	30	5.6%	-0.50 [-1.01, 0.01]
O'Brien 2006	-0.4	0.8	39	0	0.4	31	5.9%	-0.40 [-0.69, -0.11]
O'Brien 2010	-0.4	1.1	24	0.2	0.5	18	5.6%	-0.60 [-1.10, -0.10]
Parikh 2014	-1.6	0.9	3	0.7	4.3	24	2.9%	-2.30 [-4.30, -0.30]
SLIMM-T2D 2015	-2.6	0.6	18	-2	0.6	22	5.8%	-0.60 [-0.97, -0.23]
TRIABETES 2014	-3	2.6	22	-0.4	2.8	23	3.6%	-2.60 [-4.18, -1.02]
TRIABETES 2015	-2	0.6	20	-1.6	0.7	14	5.7%	-0.40 [-0.85, 0.05]
**Subtotal (95% CI)**			**186**			**192**	**39.7%**	**-0.68 [-0.98, -0.37]**
Heterogeneity: Tau² = 0.09; Chi² = 15.87, df = 7 (P = 0.03); I² = 56%								
Test for overall effect: Z = 4.38 (P < 0.0001)								
**2.4.4 Biliopancreatic diversion**								
DIBASY 2012	-5.8	3.2	19	-2.1	3	18	2.9%	-3.70 [-5.70, -1.70]
DIBASY 2015	-4.8	3.3	19	-4.1	4.1	15	2.2%	-0.70 [-3.25, 1.85]
Mingrone 2002	-0.9	0.5	46	-0.6	0.4	33	6.0%	-0.30 [-0.50, -0.10]
TRAMOMTANA 2015	-0.6	1	23	-0.1	1.6	47	5.5%	-0.50 [-1.11, 0.11]
**Subtotal (95% CI)**			**107**			**113**	**16.5%**	**-0.82 [-1.64, -0.00]**
Heterogeneity: Tau² = 0.39; Chi² = 11.35, df = 3 (P = 0.010); I² = 74%								
Test for overall effect: Z = 1.96 (P = 0.05)								
**Total (95% CI)**			**665**			**664**	**100.0%**	**-1.74 [-2.22, -1.27]**
Heterogeneity: Tau² = 0.96; Chi² = 256.26, df = 23 (P < 0.00001); I² = 91%								
Test for overall effect: Z = 7.23 (P < 0.00001)								
Test for subgroup differences: Chi² = 43.25, df = 3 (P < 0.00001), I² = 93.1%								

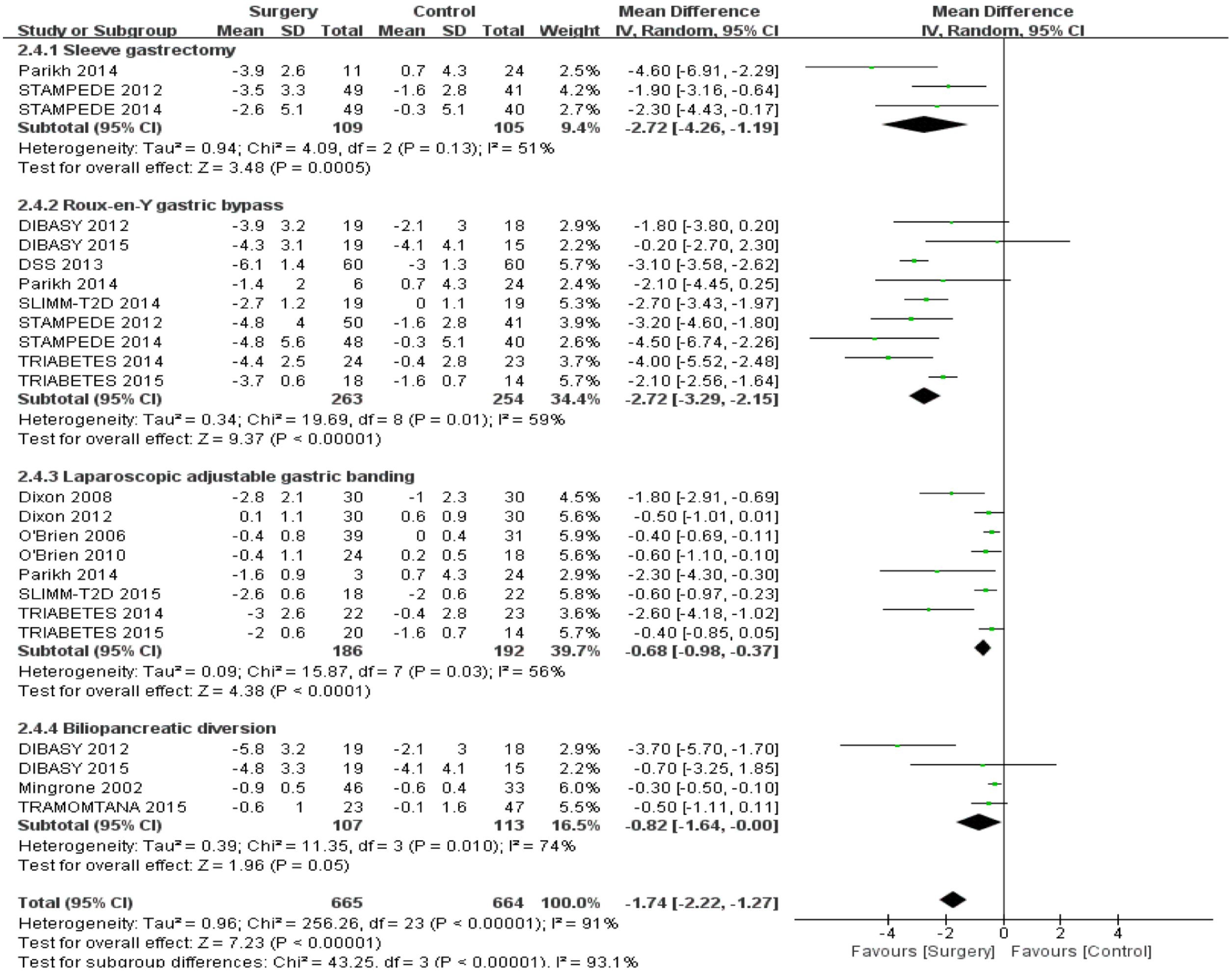

Figure S8.The forest plot of fasting glucose (mmol/L) in terms of surgical techniques

Study or Subgroup	Surgery Mean	Surgery SD	Surgery Total	Control Mean	Control SD	Control Total	Weight	Mean Difference IV, Random, 95% CI
**3.4.1 Nonsevere obesity**								
DSS 2013	-6.1	1.4	60	-3	1.3	60	8.2%	-3.10 [-3.58, -2.62]
O'Brien 2006	-0.4	0.8	39	0	0.4	31	8.5%	-0.40 [-0.69, -0.11]
Parikh 2014	-2.8	2.5	20	0.7	4.3	24	3.9%	-3.50 [-5.54, -1.46]
TRIABETES 2014	-3.7	2.6	46	-0.4	2.8	23	5.6%	-3.30 [-4.67, -1.93]
TRIABETES 2015	-2.8	1	38	-1.6	0.7	14	8.2%	-1.20 [-1.69, -0.71]
**Subtotal (95% CI)**			**203**			**152**	**34.3%**	**-2.17 [-3.49, -0.84]**
Heterogeneity: Tau² = 2.01; Chi² = 102.93, df = 4 (P < 0.00001); I² = 96%								
Test for overall effect: Z = 3.20 (P = 0.001)								
**3.4.2 Severe obesity**								
DIBASY 2012	-4.8	3.3	38	-2.1	3	18	4.6%	-2.70 [-4.44, -0.96]
DIBASY 2015	-4.6	3.2	38	-4.1	4.1	15	3.3%	-0.50 [-2.81, 1.81]
Dixon 2008	-2.8	2.1	30	-1	2.3	30	6.4%	-1.80 [-2.91, -0.69]
Dixon 2012	0.1	1.1	30	0.6	0.9	30	8.1%	-0.50 [-1.01, 0.01]
Mingrone 2002	-0.9	0.5	46	-0.6	0.4	33	8.7%	-0.30 [-0.50, -0.10]
O'Brien 2010	-0.4	1.1	24	0.2	0.5	18	8.1%	-0.60 [-1.10, -0.10]
SLIMM-T2D 2014/15	-2.7	0.9	37	-1.1	1.3	41	8.2%	-1.60 [-2.09, -1.11]
STAMPEDE 2012	-4.2	3.7	99	-1.6	2.8	41	6.3%	-2.60 [-3.73, -1.47]
STAMPEDE 2014	-3.6	5.4	97	-0.3	5.1	40	4.1%	-3.30 [-5.21, -1.39]
TRAMOMTANA 2015	-0.6	1	23	-0.1	1.6	47	7.9%	-0.50 [-1.11, 0.11]
**Subtotal (95% CI)**			**462**			**313**	**65.7%**	**-1.20 [-1.72, -0.68]**
Heterogeneity: Tau² = 0.46; Chi² = 54.72, df = 9 (P < 0.00001); I² = 84%								
Test for overall effect: Z = 4.52 (P < 0.00001)								
**Total (95% CI)**			**665**			**465**	**100.0%**	**-1.55 [-2.08, -1.01]**
Heterogeneity: Tau² = 0.85; Chi² = 178.01, df = 14 (P < 0.00001); I² = 92%								
Test for overall effect: Z = 5.65 (P < 0.00001)								
Test for subgroup differences: Chi² = 1.78, df = 1 (P = 0.18), I² = 43.7%								

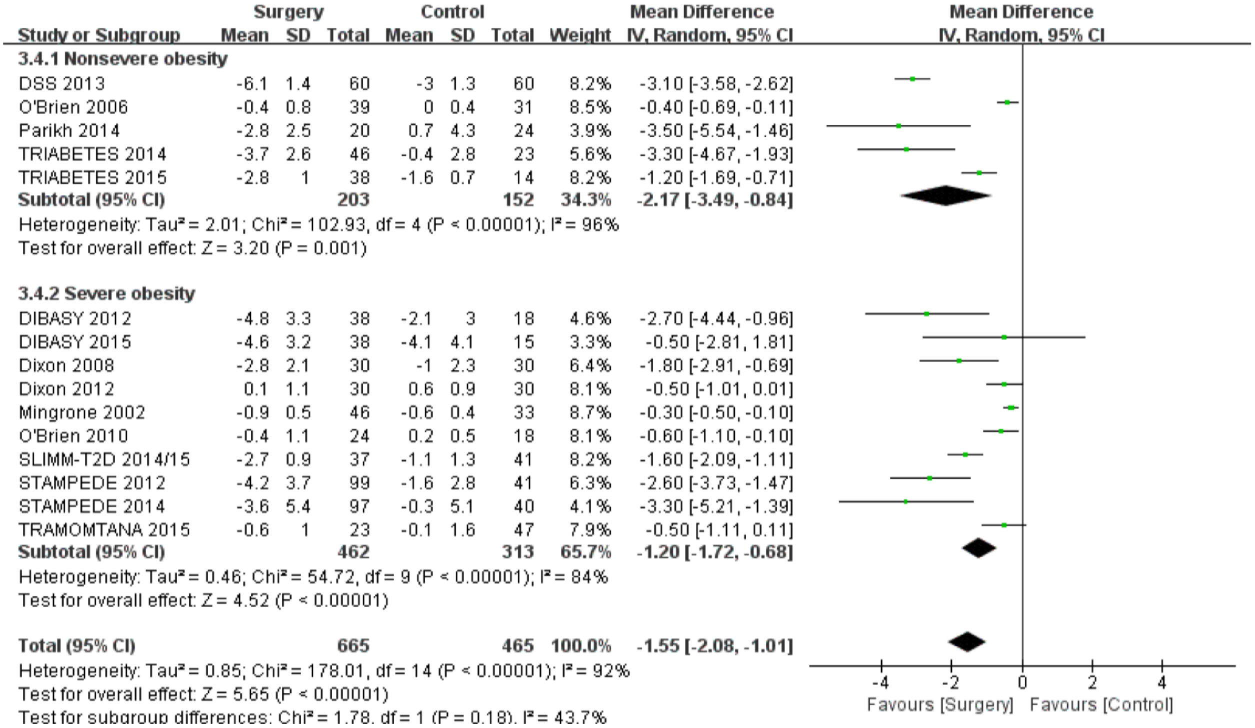

Figure S9.The forest plot of fasting glucose (mmol/L) in terms of obesity levels

Study or Subgroup	Surgery Mean	Surgery SD	Surgery Total	Control Mean	Control SD	Control Total	Weight	Mean Difference IV, Random, 95% CI
**1.5.1 1-year**								
DSS 2013	-3.3	0.3	60	-1.8	0.5	60	12.2%	-1.50 [-1.65, -1.35]
Liang 2013	-4.5	1.1	31	-3.1	1.4	70	8.7%	-1.40 [-1.91, -0.89]
Parikh 2014	-1.2	1.1	20	0.1	1.5	24	6.1%	-1.30 [-2.07, -0.53]
SLIMM-T2D 2014/15	-1.6	0.7	37	-0.5	0.8	41	10.6%	-1.10 [-1.43, -0.77]
STAMPEDE 2012	-2.9	1.7	99	-1.4	1.5	41	8.0%	-1.50 [-2.07, -0.93]
TRIABETES 2014	-1.7	1.3	46	-0.1	1.3	23	7.2%	-1.60 [-2.25, -0.95]
**Subtotal (95% CI)**			**293**			**259**	**52.8%**	**-1.43 [-1.56, -1.31]**
Heterogeneity: Tau² = 0.00; Chi² = 5.07, df = 5 (P = 0.41); I² = 1%								
Test for overall effect: Z = 22.10 (P < 0.00001)								
**1.5.2 2-year**								
DIBASY 2012	-3.1	1.7	38	-0.8	1.1	18	6.4%	-2.30 [-3.04, -1.56]
Dixon 2008	-1.8	1.2	30	-0.4	1.3	30	7.4%	-1.40 [-2.03, -0.77]
Dixon 2012	-0.3	0.9	30	0	0.5	30	10.2%	-0.30 [-0.67, 0.07]
TRAMOMTANA 2015	-8.4	9.8	10	-1.5	16.3	36	0.1%	-6.90 [-14.98, 1.18]
**Subtotal (95% CI)**			**108**			**114**	**24.0%**	**-1.42 [-2.62, -0.22]**
Heterogeneity: Tau² = 1.06; Chi² = 28.29, df = 3 (P < 0.00001); I² = 89%								
Test for overall effect: Z = 2.32 (P = 0.02)								
**1.5.3 3-year or more**								
DIBASY 2015	-2.3	1.7	38	-1.6	1	15	6.4%	-0.70 [-1.44, 0.04]
STAMPEDE 2014	-2.5	2	97	-0.6	2.5	40	5.3%	-1.90 [-2.77, -1.03]
TRIABETES 2015	-1.1	0.4	38	0.2	0.4	14	11.5%	-1.30 [-1.55, -1.05]
**Subtotal (95% CI)**			**173**			**69**	**23.2%**	**-1.27 [-1.77, -0.77]**
Heterogeneity: Tau² = 0.11; Chi² = 4.33, df = 2 (P = 0.11); I² = 54%								
Test for overall effect: Z = 4.96 (P < 0.00001)								
**Total (95% CI)**			**574**			**442**	**100.0%**	**-1.32 [-1.58, -1.05]**
Heterogeneity: Tau² = 0.14; Chi² = 50.95, df = 12 (P < 0.00001); I² = 76%								
Test for overall effect: Z = 9.78 (P < 0.00001)								
Test for subgroup differences: Chi² = 0.38, df = 2 (P = 0.83), I² = 0%								

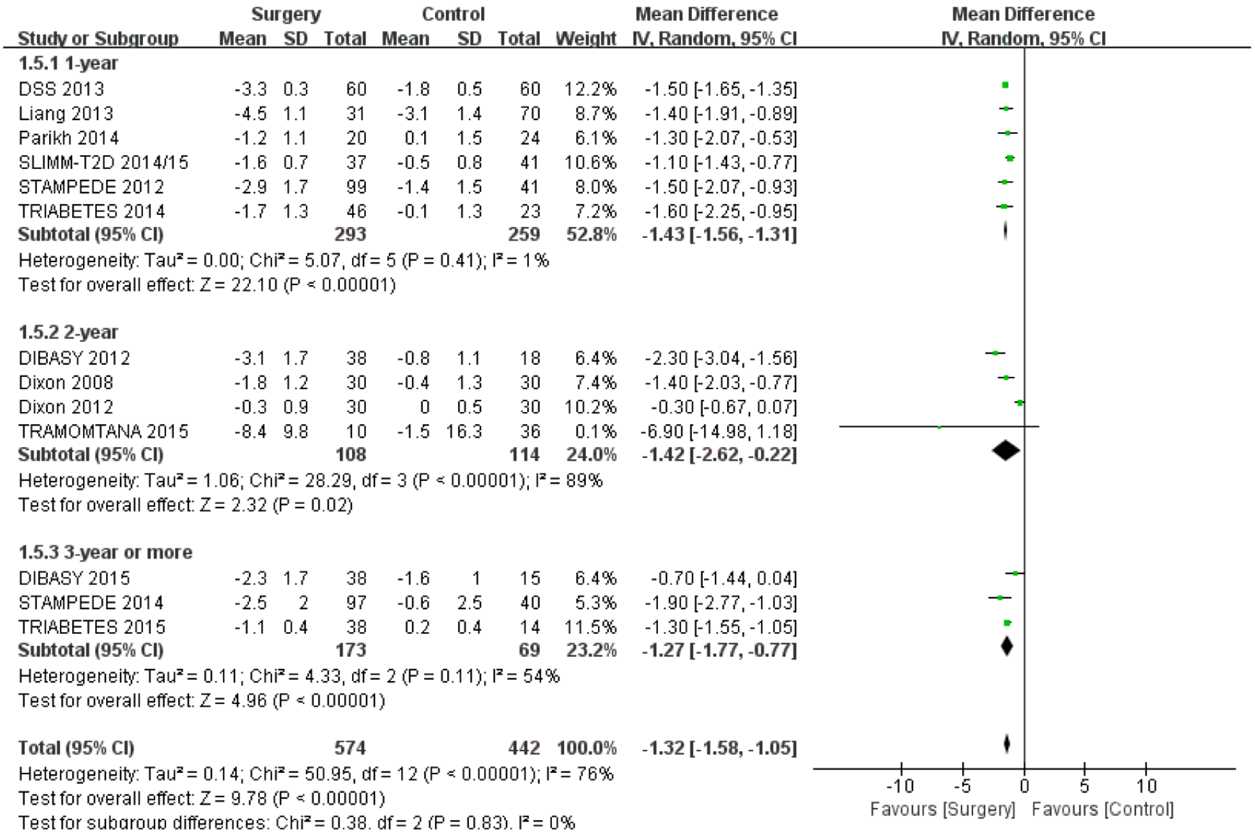

Figure S10.The forest plot of glycated hemoglobin (%) in terms of follow-up duration

Study or Subgroup	Surgery Mean	SD	Total	Control Mean	SD	Total	Weight	Mean Difference IV, Random, 95% CI
**2.5.1 Sleeve gastrectomy**								
STAMPEDE 2012	-2.9	1.8	49	-1.4	1.5	41	5.4%	-1.50 [-2.18, -0.82]
STAMPEDE 2014	-2.5	2.1	49	-0.6	2.5	40	4.4%	-1.90 [-2.87, -0.93]
**Subtotal (95% CI)**			**98**			**81**	**9.8%**	**-1.63 [-2.19, -1.07]**
Heterogeneity: Tau² = 0.00; Chi² = 0.44, df = 1 (P = 0.51); I² = 0%								
Test for overall effect: Z = 5.73 (P < 0.00001)								
**2.5.2 Roux-en-Y gastric bypass**								
DIBASY 2012	-2.2	1.4	19	-0.8	1.1	18	4.9%	-1.40 [-2.21, -0.59]
DIBASY 2015	-2	1.5	19	-1.6	1	15	4.8%	-0.40 [-1.24, 0.44]
DSS 2013	-3.3	0.3	60	-1.8	0.5	60	6.7%	-1.50 [-1.65, -1.35]
Liang 2013	-4.5	1.1	31	-3.1	1.4	70	5.9%	-1.40 [-1.91, -0.89]
SLIMM-T2D 2014	-2	0.7	19	0.1	0.7	19	6.1%	-2.10 [-2.55, -1.65]
STAMPEDE 2012	-2.9	1.6	50	-1.4	1.5	41	5.5%	-1.50 [-2.14, -0.86]
STAMPEDE 2014	-2.5	1.9	48	-0.6	2.5	40	4.5%	-1.90 [-2.84, -0.96]
TRIABETES 2014	-2.3	1.2	24	-0.1	1.3	23	5.3%	-2.20 [-2.92, -1.48]
TRIABETES 2015	-1.4	0.3	18	0.2	0.4	14	6.6%	-1.60 [-1.85, -1.35]
**Subtotal (95% CI)**			**288**			**300**	**50.4%**	**-1.59 [-1.82, -1.36]**
Heterogeneity: Tau² = 0.05; Chi² = 17.78, df = 8 (P = 0.02); I² = 55%								
Test for overall effect: Z = 13.63 (P < 0.00001)								
**2.5.3 Laparoscopic adjustable gastric banding**								
Dixon 2008	-1.8	1.2	30	-0.4	1.3	30	5.5%	-1.40 [-2.03, -0.77]
Dixon 2012	-0.3	0.9	30	0	0.5	30	6.3%	-0.30 [-0.67, 0.07]
SLIMM-T2D 2015	-1.2	0.3	18	-1	0.3	22	6.7%	-0.20 [-0.39, -0.01]
TRIABETES 2014	-1.1	1.2	22	-0.1	1.3	23	5.2%	-1.00 [-1.73, -0.27]
TRIABETES 2015	-0.8	0.3	20	0.2	0.4	14	6.6%	-1.00 [-1.25, -0.75]
**Subtotal (95% CI)**			**120**			**119**	**30.4%**	**-0.73 [-1.20, -0.26]**
Heterogeneity: Tau² = 0.23; Chi² = 36.18, df = 4 (P < 0.00001); I² = 89%								
Test for overall effect: Z = 3.07 (P = 0.002)								
**2.5.4 Biliopancreatic diversion**								
DIBASY 2012	-4	1.5	19	-0.8	1.1	18	4.8%	-3.20 [-4.04, -2.36]
DIBASY 2015	-2.5	1.8	19	-1.6	1	15	4.5%	-0.90 [-1.85, 0.05]
TRAMOMTANA 2015	-8.4	9.8	10	-1.5	16.3	36	0.2%	-6.90 [-14.98, 1.18]
**Subtotal (95% CI)**			**48**			**69**	**9.5%**	**-2.37 [-4.56, -0.18]**
Heterogeneity: Tau² = 2.45; Chi² = 13.81, df = 2 (P = 0.001); I² = 86%								
Test for overall effect: Z = 2.12 (P = 0.03)								
**Total (95% CI)**			**554**			**569**	**100.0%**	**-1.39 [-1.73, -1.05]**
Heterogeneity: Tau² = 0.45; Chi² = 217.65, df = 18 (P < 0.00001); I² = 92%								
Test for overall effect: Z = 7.95 (P < 0.00001)								
Test for subgroup differences: Chi² = 11.68, df = 3 (P = 0.009), I² = 74.3%								

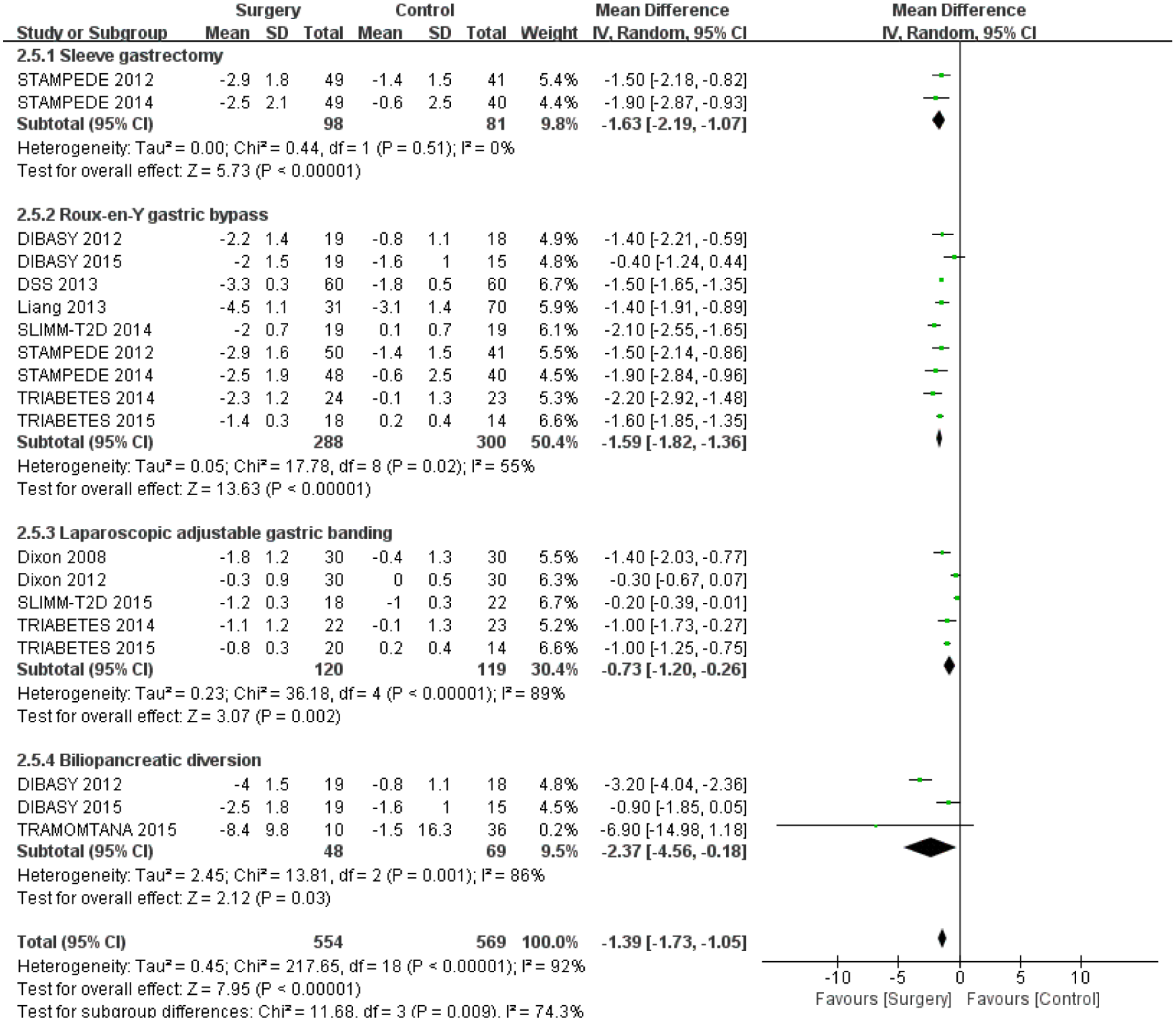

Figure S11.The forest plot of glycated hemoglobin (%) in terms of surgical techniques

Study or Subgroup	Surgery Mean	Surgery SD	Surgery Total	Control Mean	Control SD	Control Total	Weight	Mean Difference IV, Random, 95% CI
**3.5.1 Nonsevere obesity**								
DSS 2013	-3.3	0.3	60	-1.8	0.5	60	12.2%	-1.50 [-1.65, -1.35]
Liang 2013	-4.5	1.1	31	-3.1	1.4	70	8.7%	-1.40 [-1.91, -0.89]
Parikh 2014	-1.2	1.1	20	0.1	1.5	24	6.1%	-1.30 [-2.07, -0.53]
TRIABETES 2014	-1.7	1.3	46	-0.1	1.3	23	7.2%	-1.60 [-2.25, -0.95]
TRIABETES 2015	-1.1	0.4	38	0.2	0.4	14	11.5%	-1.30 [-1.55, -1.05]
**Subtotal (95% CI)**			**195**			**191**	**45.7%**	**-1.45 [-1.56, -1.33]**
Heterogeneity: Tau² = 0.00; Chi² = 2.26, df = 4 (P = 0.69); I² = 0%								
Test for overall effect: Z = 23.80 (P < 0.00001)								
**3.5.2 Severe obesity**								
DIBASY 2012	-3.1	1.7	38	-0.8	1.1	18	6.4%	-2.30 [-3.04, -1.56]
DIBASY 2015	-2.3	1.7	38	-1.6	1	15	6.4%	-0.70 [-1.44, 0.04]
Dixon 2008	-1.8	1.2	30	-0.4	1.3	30	7.4%	-1.40 [-2.03, -0.77]
Dixon 2012	-0.3	0.9	30	0	0.5	30	10.2%	-0.30 [-0.67, 0.07]
SLIMM-T2D 2014/15	-1.6	0.7	37	-0.5	0.8	41	10.6%	-1.10 [-1.43, -0.77]
STAMPEDE 2012	-2.9	1.7	99	-1.4	1.5	41	8.0%	-1.50 [-2.07, -0.93]
STAMPEDE 2014	-2.5	2	97	-0.6	2.5	40	5.3%	-1.90 [-2.77, -1.03]
TRAMOMTANA 2015	-8.4	9.8	10	-1.5	16.3	36	0.1%	-6.90 [-14.98, 1.18]
**Subtotal (95% CI)**			**379**			**251**	**54.3%**	**-1.29 [-1.80, -0.78]**
Heterogeneity: Tau² = 0.37; Chi² = 37.03, df = 7 (P < 0.00001); I² = 81%								
Test for overall effect: Z = 4.99 (P < 0.00001)								
**Total (95% CI)**			**574**			**442**	**100.0%**	**-1.32 [-1.58, -1.05]**
Heterogeneity: Tau² = 0.14; Chi² = 50.95, df = 12 (P < 0.00001); I² = 76%								
Test for overall effect: Z = 9.78 (P < 0.00001)								
Test for subgroup differences: Chi² = 0.35, df = 1 (P = 0.56), I² = 0%								

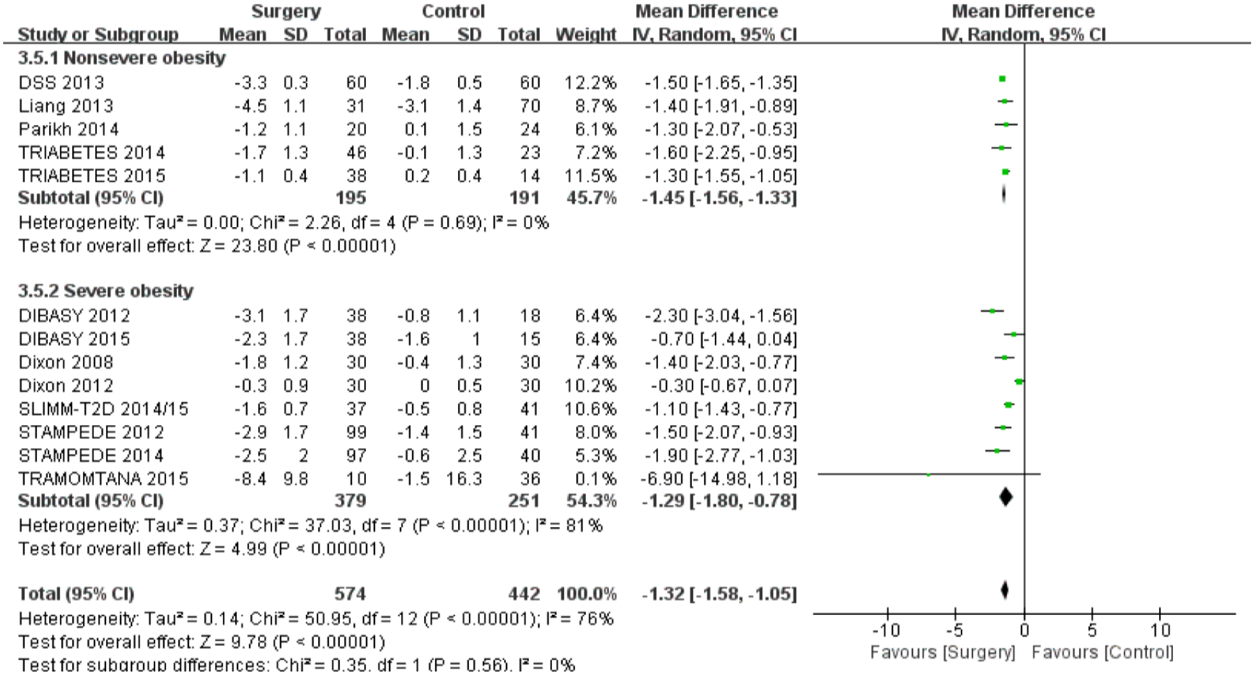

Figure S12.The forest plot of glycated hemoglobin (%) in terms of obesity levels

Study or Subgroup	Surgery Mean	Surgery SD	Surgery Total	Control Mean	Control SD	Control Total	Weight	Mean Difference IV, Random, 95% CI
**1.6.1 1-year**								
DSS 2013	-24	4.1	60	-8	4.4	60	15.1%	-16.00 [-17.52, -14.48]
Parikh 2014	-16.1	8.9	20	-1.8	5.1	24	6.5%	-14.30 [-18.70, -9.90]
SLIMM-T2D 2014/15	-18.3	10.6	37	-7.2	6	41	7.7%	-11.10 [-14.98, -7.22]
STAMPEDE 2012	-21.6	8.7	99	-4.1	8.5	41	9.6%	-17.50 [-20.62, -14.38]
TRIABETES 2014	-21.1	6.8	46	-8.1	6.1	23	9.5%	-13.00 [-16.17, -9.83]
**Subtotal (95% CI)**			**262**			**189**	**48.4%**	**-14.69 [-16.76, -12.63]**
Heterogeneity: Tau² = 3.03; Chi² = 9.44, df = 4 (P = 0.05); I² = 58%								
Test for overall effect: Z = 13.94 (P < 0.00001)								
**1.6.2 2-year**								
DIBASY 2012	-26.8	16.7	38	-10.6	13.6	18	2.5%	-16.20 [-24.43, -7.97]
Dixon 2008	-17.9	10.8	30	-4	9.1	30	5.4%	-13.90 [-18.95, -8.85]
Dixon 2012	-18.1	8.1	30	-2.9	4.1	30	9.3%	-15.20 [-18.45, -11.95]
O'Brien 2006	-15.7	6.9	39	-1.4	8.4	31	8.2%	-14.30 [-17.97, -10.63]
O'Brien 2010	-28.2	12.4	24	-3.5	14.6	18	2.4%	-24.70 [-33.07, -16.33]
**Subtotal (95% CI)**			**161**			**127**	**27.8%**	**-15.56 [-18.13, -13.00]**
Heterogeneity: Tau² = 2.28; Chi² = 5.47, df = 4 (P = 0.24); I² = 27%								
Test for overall effect: Z = 11.88 (P < 0.00001)								
**1.6.3 3-year or more**								
DIBASY 2015	-25.6	13.5	38	-13.8	12.3	15	2.9%	-11.80 [-19.36, -4.24]
STAMPEDE 2014	-17.1	10.1	97	-1.4	11.4	40	7.2%	-15.70 [-19.76, -11.64]
TRIABETES 2015	-16.9	4.9	38	-4.8	2.2	14	13.6%	-12.10 [-14.04, -10.16]
**Subtotal (95% CI)**			**173**			**69**	**23.7%**	**-12.98 [-15.26, -10.70]**
Heterogeneity: Tau² = 1.06; Chi² = 2.51, df = 2 (P = 0.28); I² = 20%								
Test for overall effect: Z = 11.17 (P < 0.00001)								
**Total (95% CI)**			**596**			**385**	**100.0%**	**-14.59 [-15.99, -13.19]**
Heterogeneity: Tau² = 2.80; Chi² = 23.87, df = 12 (P = 0.02); I² = 50%								
Test for overall effect: Z = 20.36 (P < 0.00001)								
Test for subgroup differences: Chi² = 2.35, df = 2 (P = 0.31), I² = 14.9%								

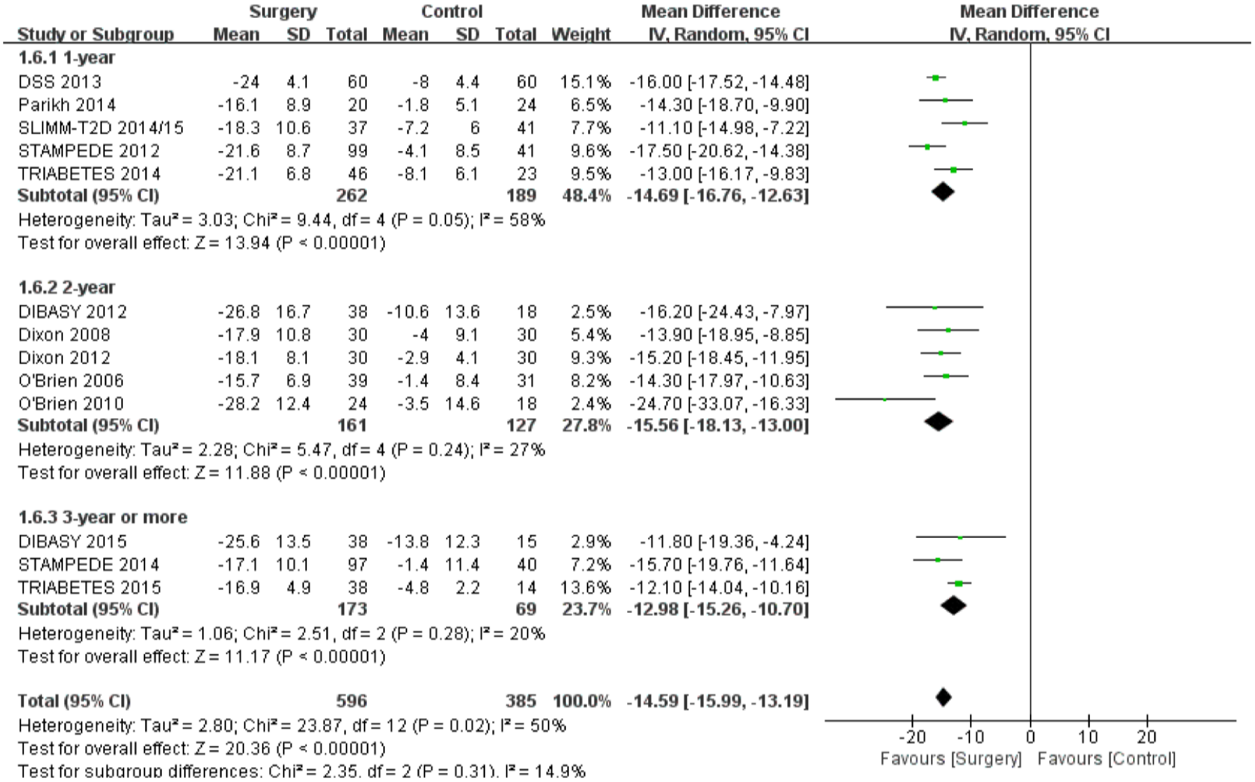

Figure S13.The forest plot of waist circumference (cm) in terms of follow-up duration

Study or Subgroup	Surgery Mean	Surgery SD	Surgery Total	Control Mean	Control SD	Control Total	Weight	Mean Difference IV, Random, 95% CI
**2.6.1 Sleeve gastrectomy**								
STAMPEDE 2012	-20.1	9	49	-4.1	8.5	41	5.8%	-16.00 [-19.62, -12.38]
STAMPEDE 2014	-14.8	10.1	49	-1.4	11.4	40	5.4%	-13.40 [-17.93, -8.87]
**Subtotal (95% CI)**			**98**			**81**	**11.3%**	**-14.98 [-17.81, -12.16]**
Heterogeneity: Tau² = 0.00; Chi² = 0.77, df = 1 (P = 0.38); I² = 0%								
Test for overall effect: Z = 10.39 (P < 0.00001)								
**2.6.2 Roux-en-Y gastric bypass**								
DIBASY 2012	-26.8	15.1	19	-10.6	13.6	18	3.3%	-16.20 [-25.45, -6.95]
DIBASY 2015	-22.4	12.5	19	-13.8	12.3	15	3.6%	-8.60 [-16.99, -0.21]
DSS 2013	-24	4.1	60	-8	4.4	60	6.6%	-16.00 [-17.52, -14.48]
SLIMM-T2D 2014	-26.9	5.1	19	-6.6	5.5	19	6.0%	-20.30 [-23.67, -16.93]
STAMPEDE 2012	-23	8.3	50	-4.1	8.5	41	5.9%	-18.90 [-22.37, -15.43]
STAMPEDE 2014	-19.4	9.6	48	-1.4	11.4	40	5.5%	-18.00 [-22.46, -13.54]
TRIABETES 2014	-25	5.7	24	-8.1	6.1	23	6.0%	-16.90 [-20.28, -13.52]
TRIABETES 2015	-21.5	1.9	18	-4.8	2.2	14	6.6%	-16.70 [-18.15, -15.25]
**Subtotal (95% CI)**			**257**			**230**	**43.5%**	**-17.07 [-18.38, -15.75]**
Heterogeneity: Tau² = 1.05; Chi² = 10.59, df = 7 (P = 0.16); I² = 34%								
Test for overall effect: Z = 25.45 (P < 0.00001)								
**2.6.3 Laparoscopic adjustable gastric banding**								
Dixon 2008	-17.9	10.8	30	-4	9.1	30	5.2%	-13.90 [-18.95, -8.85]
Dixon 2012	-18.1	8.1	30	-2.9	4.1	30	6.0%	-15.20 [-18.45, -11.95]
O'Brien 2006	-15.7	6.9	39	-1.4	8.4	31	5.8%	-14.30 [-17.97, -10.63]
O'Brien 2010	-28.2	12.4	24	-3.5	14.6	18	3.6%	-24.70 [-33.07, -16.33]
SLIMM-T2D 2015	-9.2	6.4	18	-7.8	6.5	22	5.7%	-1.40 [-5.41, 2.61]
TRIABETES 2014	-16.8	5	22	-8.1	6.1	23	6.0%	-8.70 [-11.95, -5.45]
TRIABETES 2015	-12.7	2	20	-4.8	2.2	14	6.6%	-7.90 [-9.35, -6.45]
**Subtotal (95% CI)**			**183**			**168**	**39.0%**	**-11.56 [-15.44, -7.68]**
Heterogeneity: Tau² = 22.70; Chi² = 54.53, df = 6 (P < 0.00001); I² = 89%								
Test for overall effect: Z = 5.84 (P < 0.00001)								
**2.6.4 Biliopancreatic diversion**								
DIBASY 2012	-26.8	18.5	19	-10.6	13.6	18	2.9%	-16.20 [-26.62, -5.78]
DIBASY 2015	-28.8	14.1	19	-13.8	12.3	15	3.4%	-15.00 [-23.88, -6.12]
**Subtotal (95% CI)**			**38**			**33**	**6.3%**	**-15.50 [-22.27, -8.74]**
Heterogeneity: Tau² = 0.00; Chi² = 0.03, df = 1 (P = 0.86); I² = 0%								
Test for overall effect: Z = 4.49 (P < 0.00001)								
**Total (95% CI)**			**576**			**512**	**100.0%**	**-14.46 [-16.80, -12.13]**
Heterogeneity: Tau² = 20.90; Chi² = 173.17, df = 18 (P < 0.00001); I² = 90%								
Test for overall effect: Z = 12.13 (P < 0.00001)								
Test for subgroup differences: Chi² = 7.91, df = 3 (P = 0.05), I² = 62.1%								

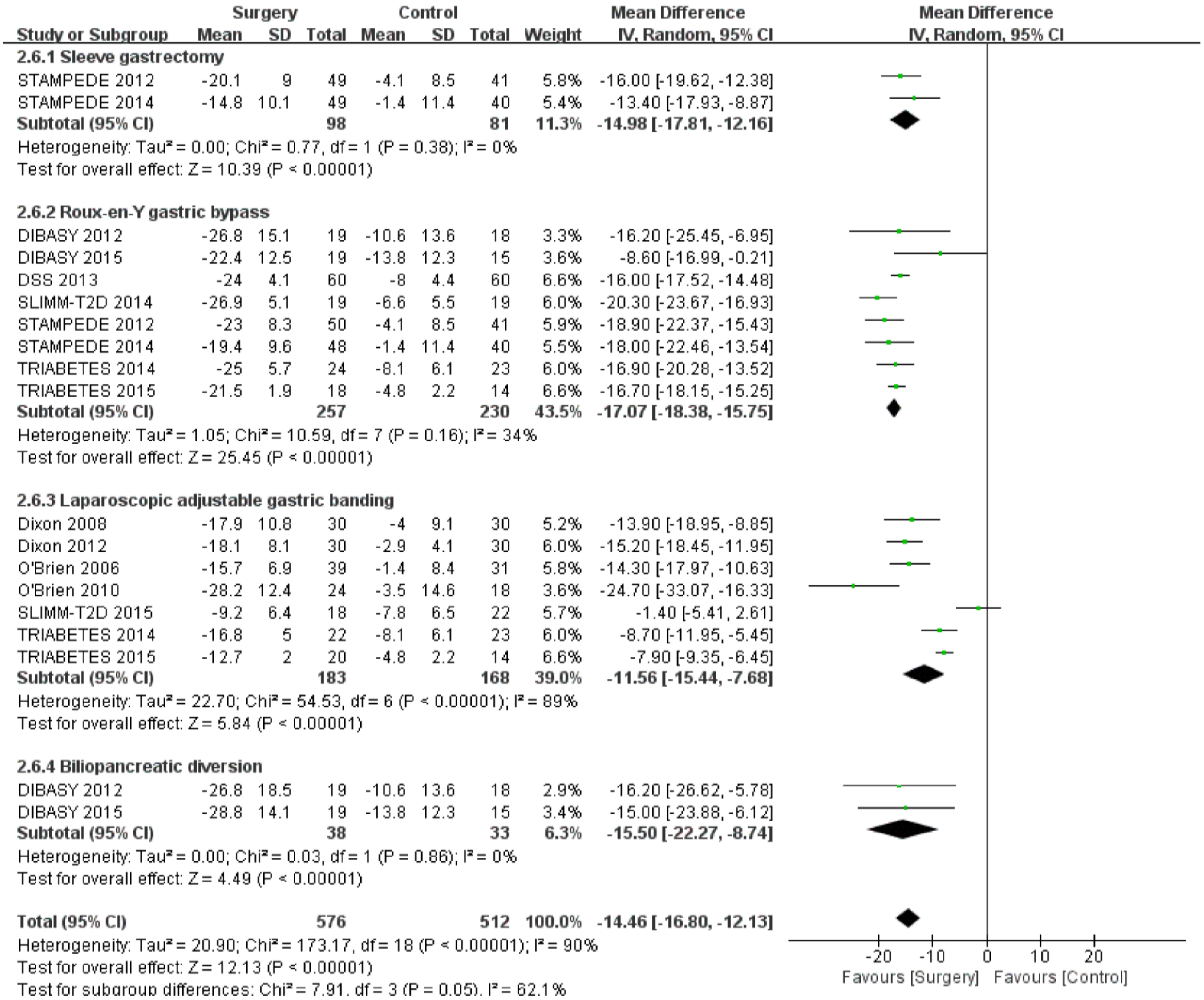

Figure S14.The forest plot of waist circumference (cm) in terms of surgical techniques

Study or Subgroup	Surgery Mean	Surgery SD	Surgery Total	Control Mean	Control SD	Control Total	Weight	Mean Difference IV, Random, 95% CI
**3.6.1 Nonsevere obesity**								
DSS 2013	-24	4.1	60	-8	4.4	60	15.1%	-16.00 [-17.52, -14.48]
O'Brien 2006	-15.7	6.9	39	-1.4	8.4	31	8.2%	-14.30 [-17.97, -10.63]
Parikh 2014	-16.1	8.9	20	-1.8	5.1	24	6.5%	-14.30 [-18.70, -9.90]
TRIABETES 2014	-21.1	6.8	46	-8.1	6.1	23	9.5%	-13.00 [-16.17, -9.83]
TRIABETES 2015	-16.9	4.9	38	-4.8	2.2	14	13.6%	-12.10 [-14.04, -10.16]
**Subtotal (95% CI)**			**203**			**152**	**52.9%**	**-13.99 [-15.89, -12.09]**
Heterogeneity: Tau² = 2.66; Chi² = 10.39, df = 4 (P = 0.03); I² = 61%								
Test for overall effect: Z = 14.42 (P < 0.00001)								
**3.6.2 Severe obesity**								
DIBASY 2012	-26.8	16.7	38	-10.6	13.6	18	2.5%	-16.20 [-24.43, -7.97]
DIBASY 2015	-25.6	13.5	38	-13.8	12.3	15	2.9%	-11.80 [-19.36, -4.24]
Dixon 2008	-17.9	10.8	30	-4	9.1	30	5.4%	-13.90 [-18.95, -8.85]
Dixon 2012	-18.1	8.1	30	-2.9	4.1	30	9.3%	-15.20 [-18.45, -11.95]
O'Brien 2010	-28.2	12.4	24	-3.5	14.6	18	2.4%	-24.70 [-33.07, -16.33]
SLIMM-T2D 2014/15	-18.3	10.6	37	-7.2	6	41	7.7%	-11.10 [-14.98, -7.22]
STAMPEDE 2012	-21.6	8.7	99	-4.1	8.5	41	9.6%	-17.50 [-20.62, -14.38]
STAMPEDE 2014	-17.1	10.1	97	-1.4	11.4	40	7.2%	-15.70 [-19.76, -11.64]
**Subtotal (95% CI)**			**393**			**233**	**47.1%**	**-15.29 [-17.52, -13.05]**
Heterogeneity: Tau² = 4.22; Chi² = 12.47, df = 7 (P = 0.09); I² = 44%								
Test for overall effect: Z = 13.39 (P < 0.00001)								
**Total (95% CI)**			**596**			**385**	**100.0%**	**-14.59 [-15.99, -13.19]**
Heterogeneity: Tau² = 2.80; Chi² = 23.87, df = 12 (P = 0.02); I² = 50%								
Test for overall effect: Z = 20.36 (P < 0.00001)								
Test for subgroup differences: Chi² = 0.75, df = 1 (P = 0.39), I² = 0%								

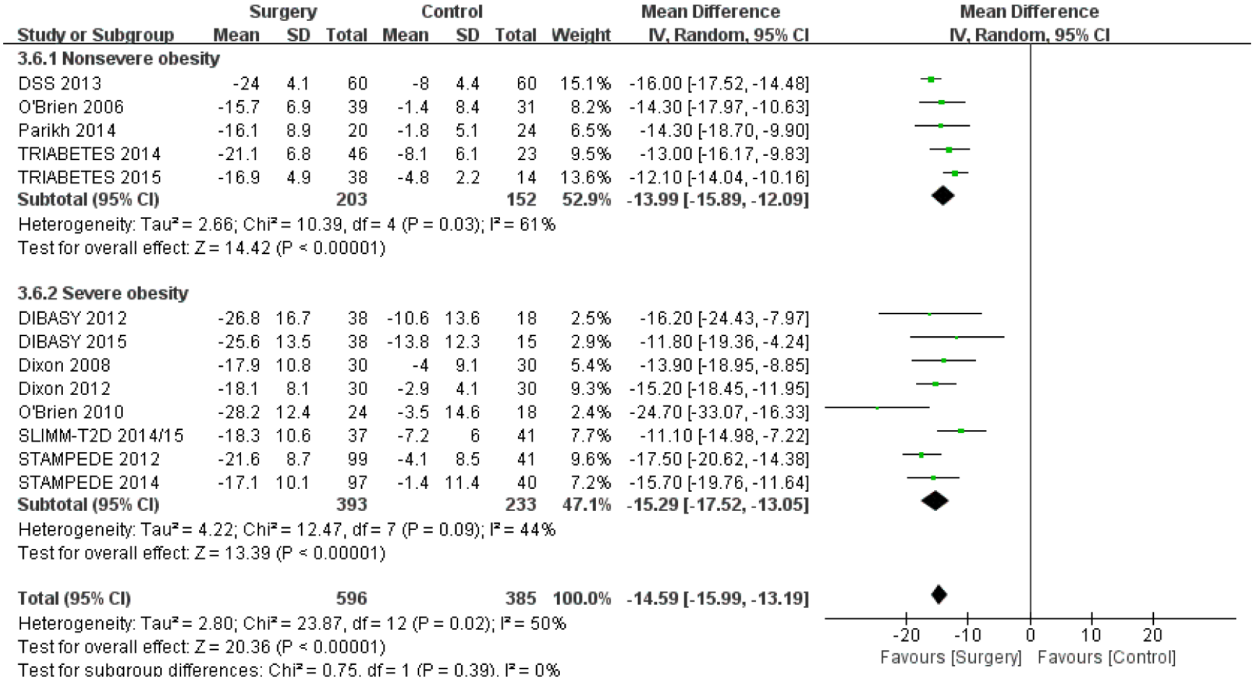

Figure S15.The forest plot of waist circumference (cm) in terms of obesity levels

Study or Subgroup	Surgery Mean	Surgery SD	Surgery Total	Control Mean	Control SD	Control Total	Weight	Mean Difference IV, Random, 95% CI
**1.7.1 1-year**								
DSS 2013	-12	5.2	60	-8	4.9	60	31.0%	-4.00 [-5.81, -2.19]
Liang 2013	-34.3	6.8	31	-26.6	9.3	70	14.5%	-7.70 [-10.94, -4.46]
Parikh 2014	-0.4	19.7	20	2.5	19.1	24	1.4%	-2.90 [-14.43, 8.63]
SLIMM-T2D 2014/15	-5.2	11.1	37	-3	8.3	41	8.8%	-2.20 [-6.59, 2.19]
STAMPEDE 2012	-3.7	17.8	99	-3.9	14.7	41	5.5%	0.20 [-5.50, 5.90]
TRIABETES 2014	-9.8	12.4	46	-8.8	12.2	23	4.8%	-1.00 [-7.14, 5.14]
**Subtotal (95% CI)**			**293**			**259**	**66.1%**	**-3.72 [-5.99, -1.44]**
Heterogeneity: Tau² = 3.08; Chi² = 8.69, df = 5 (P = 0.12); I² = 42%								
Test for overall effect: Z = 3.20 (P = 0.001)								
**1.7.2 2-year**								
DIBASY 2012	-19.5	23.1	38	-20.8	30.2	18	0.8%	1.30 [-14.47, 17.07]
Dixon 2008	-6	17.9	30	-1.7	14.2	30	2.8%	-4.30 [-12.48, 3.88]
Dixon 2012	-7.3	12.3	30	-5.9	9.2	30	5.9%	-1.40 [-6.90, 4.10]
O'Brien 2006	-14.2	14.2	39	-9.4	12.6	31	4.6%	-4.80 [-11.09, 1.49]
O'Brien 2010	-12.5	17.6	24	-20.3	21.7	18	1.3%	7.80 [-4.45, 20.05]
TRAMOMTANA 2015	1.9	15.9	23	1.6	12.3	47	3.4%	0.30 [-7.09, 7.69]
**Subtotal (95% CI)**			**184**			**174**	**18.8%**	**-1.65 [-4.78, 1.47]**
Heterogeneity: Tau² = 0.00; Chi² = 4.06, df = 5 (P = 0.54); I² = 0%								
Test for overall effect: Z = 1.04 (P = 0.30)								
**1.7.3 3-year or more**								
DIBASY 2015	-20.6	23.9	38	-25.2	35.9	15	0.5%	4.60 [-15.09, 24.29]
STAMPEDE 2014	-1.6	20.6	97	0.6	22.6	40	2.8%	-2.20 [-10.32, 5.92]
TRIABETES 2015	-4.7	8.8	38	-0.2	4.6	14	11.8%	-4.50 [-8.19, -0.81]
**Subtotal (95% CI)**			**173**			**69**	**15.1%**	**-3.86 [-7.17, -0.55]**
Heterogeneity: Tau² = 0.00; Chi² = 0.99, df = 2 (P = 0.61); I² = 0%								
Test for overall effect: Z = 2.28 (P = 0.02)								
**Total (95% CI)**			**650**			**502**	**100.0%**	**-3.50 [-4.90, -2.10]**
Heterogeneity: Tau² = 0.80; Chi² = 15.68, df = 14 (P = 0.33); I² = 11%								
Test for overall effect: Z = 4.90 (P < 0.00001)								
Test for subgroup differences: Chi² = 1.29, df = 2 (P = 0.53), I² = 0%								

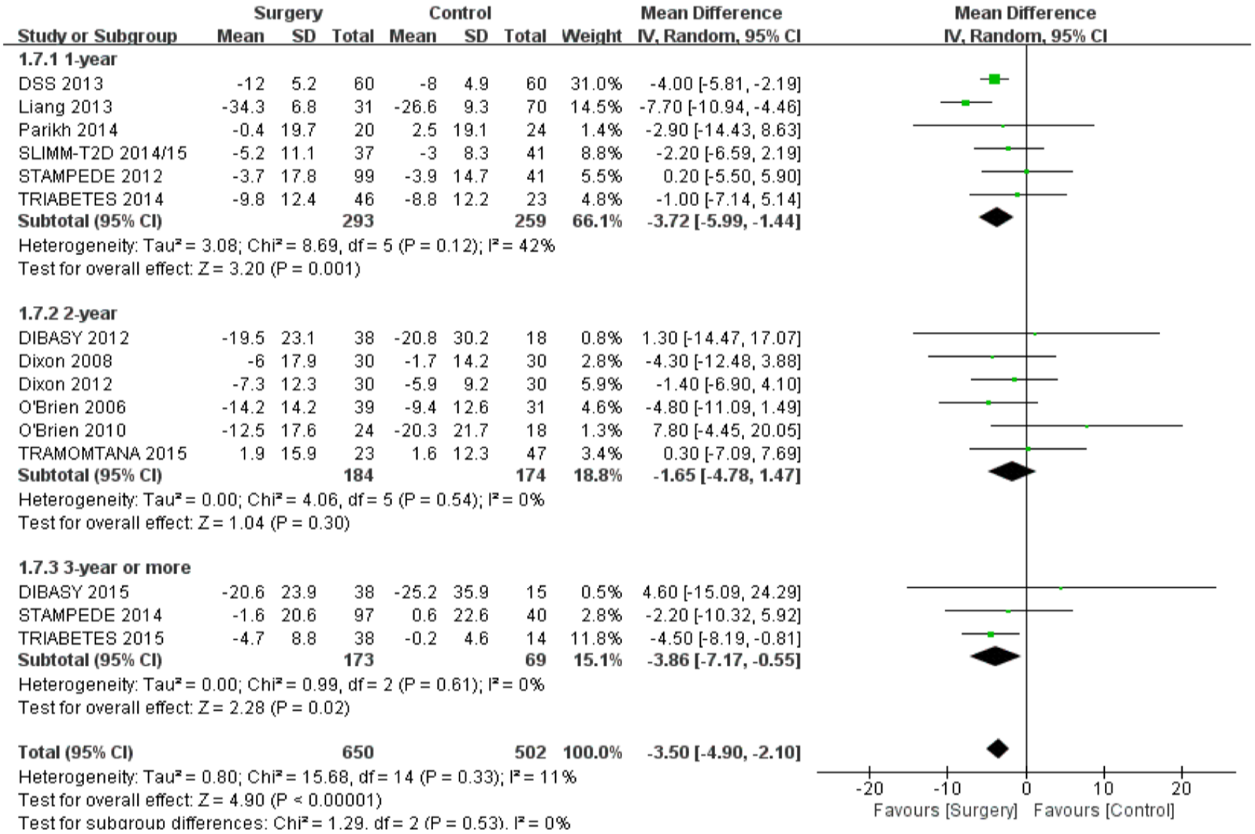

Figure S16.The forest plot of systolic pressure (mmHg) in terms of follow-up duration

Study or Subgroup	Surgery Mean	Surgery SD	Surgery Total	Control Mean	Control SD	Control Total	Weight	Mean Difference IV, Random, 95% CI
**2.7.1 Sleeve gastrectomy**								
STAMPEDE 2012	-5.1	17	49	-3.9	14.7	41	5.3%	-1.20 [-7.75, 5.35]
STAMPEDE 2014	-4.4	20.7	49	0.6	22.6	40	4.2%	-5.00 [-14.09, 4.09]
**Subtotal (95% CI)**			**98**			**81**	**9.6%**	**-2.50 [-7.81, 2.82]**
Heterogeneity: Tau² = 0.00; Chi² = 0.44, df = 1 (P = 0.51); I² = 0%								
Test for overall effect: Z = 0.92 (P = 0.36)								
**2.7.2 Roux-en-Y gastric bypass**								
DIBASY 2012	-13.6	17.8	19	-20.8	30.2	18	2.2%	7.20 [-8.88, 23.28]
DIBASY 2015	-15	18.6	19	-25.2	35.9	15	1.6%	10.20 [-9.80, 30.20]
DSS 2013	-12	5.2	60	-8	4.9	60	7.2%	-4.00 [-5.81, -2.19]
Liang 2013	-34.3	6.8	31	-26.6	9.3	70	6.8%	-7.70 [-10.94, -4.46]
SLIMM-T2D 2014	-12.3	9.1	19	-1	9.3	19	5.7%	-11.30 [-17.15, -5.45]
STAMPEDE 2012	-2.4	18.7	50	-3.9	14.7	41	5.2%	1.50 [-5.36, 8.36]
STAMPEDE 2014	1.3	20.4	48	0.6	22.6	40	4.2%	0.70 [-8.38, 9.78]
TRIABETES 2014	-14.5	11.9	24	-8.8	12.2	23	5.2%	-5.70 [-12.59, 1.19]
TRIABETES 2015	-13	4.1	18	-0.2	4.6	14	6.8%	-12.80 [-15.86, -9.74]
**Subtotal (95% CI)**			**288**			**300**	**44.9%**	**-5.19 [-8.94, -1.44]**
Heterogeneity: Tau² = 20.35; Chi² = 39.57, df = 8 (P < 0.00001); I² = 80%								
Test for overall effect: Z = 2.71 (P = 0.007)								
**2.7.3 Laparoscopic adjustable gastric banding**								
Dixon 2008	-6	17.9	30	-1.7	14.2	30	4.6%	-4.30 [-12.48, 3.88]
Dixon 2012	-7.3	12.3	30	-5.9	9.2	30	5.8%	-1.40 [-6.90, 4.10]
O'Brien 2006	-14.2	14.2	39	-9.4	12.6	31	5.5%	-4.80 [-11.09, 1.49]
O'Brien 2010	-12.5	17.6	24	-20.3	21.7	18	3.1%	7.80 [-4.45, 20.05]
SLIMM-T2D 2015	2.2	7.6	18	-4.8	7.1	22	6.2%	7.00 [2.40, 11.60]
TRIABETES 2014	-4.6	10.9	22	-8.8	12.2	23	5.2%	4.20 [-2.55, 10.95]
TRIABETES 2015	2.7	3.8	20	-0.2	4.6	14	6.9%	2.90 [-0.03, 5.83]
**Subtotal (95% CI)**			**183**			**168**	**37.4%**	**1.59 [-1.81, 4.98]**
Heterogeneity: Tau² = 11.08; Chi² = 14.38, df = 6 (P = 0.03); I² = 58%								
Test for overall effect: Z = 0.92 (P = 0.36)								
**2.7.4 Biliopancreatic diversion**								
DIBASY 2012	-25.3	26.6	19	-20.8	30.2	18	1.8%	-4.50 [-22.88, 13.88]
DIBASY 2015	-26.1	27.6	19	-25.2	35.9	15	1.4%	-0.90 [-22.90, 21.10]
TRAMOMTANA 2015	1.9	15.9	23	1.6	12.3	47	5.0%	0.30 [-7.09, 7.69]
**Subtotal (95% CI)**			**61**			**80**	**8.1%**	**-0.42 [-6.96, 6.13]**
Heterogeneity: Tau² = 0.00; Chi² = 0.23, df = 2 (P = 0.89); I² = 0%								
Test for overall effect: Z = 0.12 (P = 0.90)								
**Total (95% CI)**			**630**			**629**	**100.0%**	**-1.99 [-4.84, 0.86]**
Heterogeneity: Tau² = 28.45; Chi² = 104.78, df = 20 (P < 0.00001); I² = 81%								
Test for overall effect: Z = 1.37 (P = 0.17)								
Test for subgroup differences: Chi² = 7.14, df = 3 (P = 0.07), I² = 58.0%								

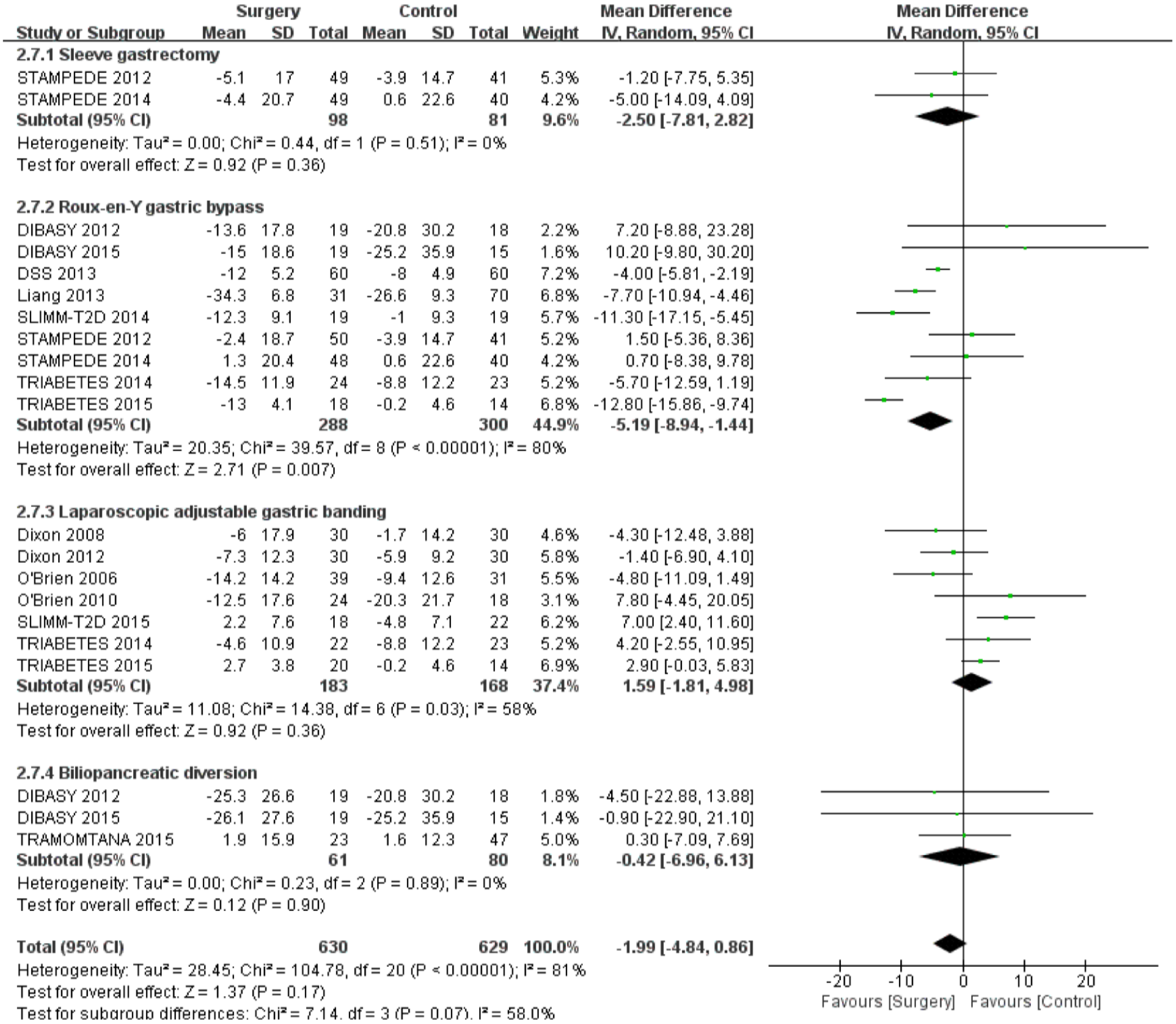

Figure S17.The forest plot of systolic pressure (mmHg) in terms of surgical techniques

Study or Subgroup	Surgery Mean	Surgery SD	Surgery Total	Control Mean	Control SD	Control Total	Weight	Mean Difference IV, Random, 95% CI
**3.7.1 Nonsevere obesity**								
DSS 2013	-12	5.2	60	-8	4.9	60	31.0%	-4.00 [-5.81, -2.19]
Liang 2013	-34.3	6.8	31	-26.6	9.3	70	14.5%	-7.70 [-10.94, -4.46]
O'Brien 2006	-14.2	14.2	39	-9.4	12.6	31	4.6%	-4.80 [-11.09, 1.49]
Parikh 2014	-0.4	19.7	20	2.5	19.1	24	1.4%	-2.90 [-14.43, 8.63]
TRIABETES 2014	-9.8	12.4	46	-8.8	12.2	23	4.8%	-1.00 [-7.14, 5.14]
TRIABETES 2015	-4.7	8.8	38	-0.2	4.6	14	11.8%	-4.50 [-8.19, -0.81]
**Subtotal (95% CI)**			**234**			**222**	**68.2%**	**-4.65 [-6.16, -3.15]**
Heterogeneity: Tau² = 0.28; Chi² = 5.36, df = 5 (P = 0.37); I² = 7%								
Test for overall effect: Z = 6.06 (P < 0.00001)								
**3.7.2 Severe obesity**								
DIBASY 2012	-19.5	23.1	38	-20.8	30.2	18	0.8%	1.30 [-14.47, 17.07]
DIBASY 2015	-20.6	23.9	38	-25.2	35.9	15	0.5%	4.60 [-15.09, 24.29]
Dixon 2008	-6	17.9	30	-1.7	14.2	30	2.8%	-4.30 [-12.48, 3.88]
Dixon 2012	-7.3	12.3	30	-5.9	9.2	30	5.9%	-1.40 [-6.90, 4.10]
O'Brien 2010	-12.5	17.6	24	-20.3	21.7	18	1.3%	7.80 [-4.45, 20.05]
SLIMM-T2D 2014/15	-5.2	11.1	37	-3	8.3	41	8.8%	-2.20 [-6.59, 2.19]
STAMPEDE 2012	-3.7	17.8	99	-3.9	14.7	41	5.5%	0.20 [-5.50, 5.90]
STAMPEDE 2014	-1.6	20.6	97	0.6	22.6	40	2.8%	-2.20 [-10.32, 5.92]
TRAMOMTANA 2015	1.9	15.9	23	1.6	12.3	47	3.4%	0.30 [-7.09, 7.69]
**Subtotal (95% CI)**			**416**			**280**	**31.8%**	**-1.00 [-3.37, 1.37]**
Heterogeneity: Tau² = 0.00; Chi² = 3.68, df = 8 (P = 0.88); I² = 0%								
Test for overall effect: Z = 0.83 (P = 0.41)								
**Total (95% CI)**			**650**			**502**	**100.0%**	**-3.50 [-4.90, -2.10]**
Heterogeneity: Tau² = 0.80; Chi² = 15.68, df = 14 (P = 0.33); I² = 11%								
Test for overall effect: Z = 4.90 (P < 0.00001)								
Test for subgroup differences: Chi² = 6.49, df = 1 (P = 0.01), I² = 84.6%								

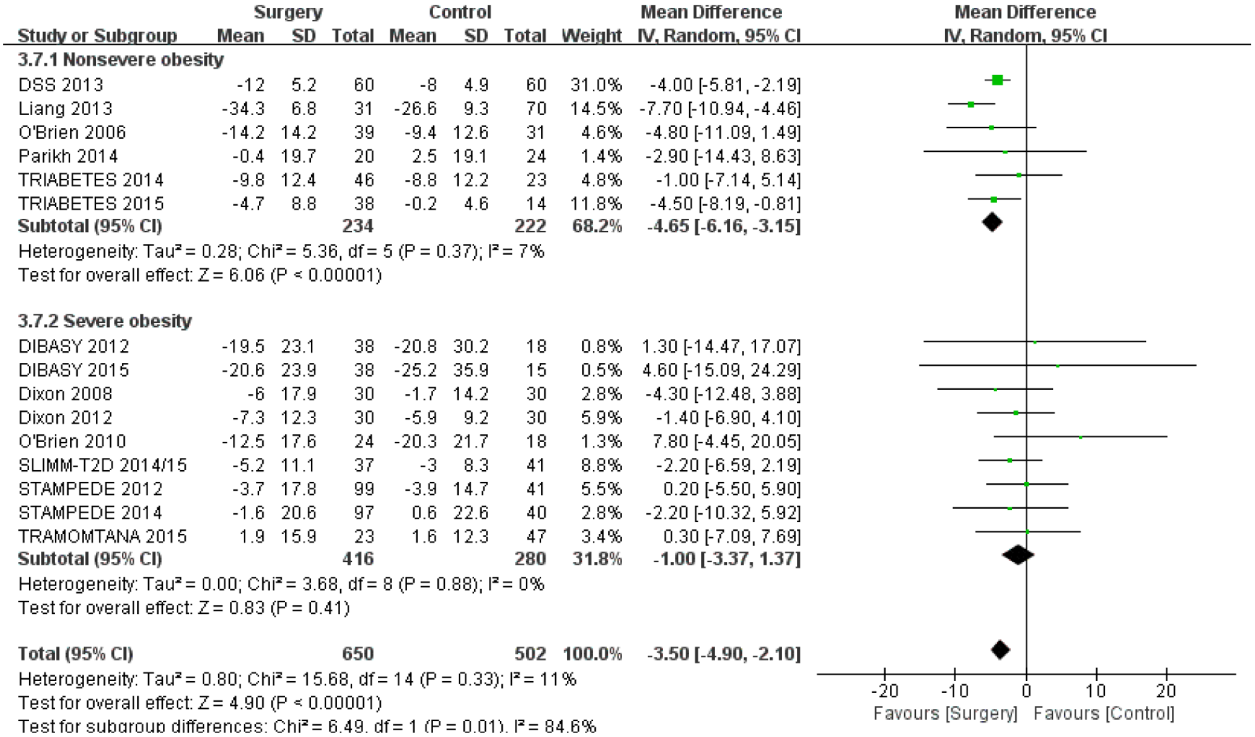

Figure S18.The forest plot of systolic pressure (mmHg) in terms of obesity levels

Study or Subgroup	Surgery Mean	Surgery SD	Surgery Total	Control Mean	Control SD	Control Total	Weight	Mean Difference IV, Random, 95% CI
**1.8.1 1-year**								
DSS 2013	-10	4.1	60	-5	3.4	60	10.9%	-5.00 [-6.35, -3.65]
Parikh 2014	0.1	10.7	20	-0.2	12.3	24	4.9%	0.30 [-6.50, 7.10]
SLIMM-T2D 2014/15	-3.2	4.9	37	-2.1	4.4	41	10.2%	-1.10 [-3.18, 0.98]
STAMPEDE 2012	-3.5	9.3	99	-4.5	8.5	41	8.9%	1.00 [-2.18, 4.18]
TRIABETES 2014	-4.6	6.5	46	-3.6	6.1	23	8.9%	-1.00 [-4.12, 2.12]
**Subtotal (95% CI)**			**262**			**189**	**43.8%**	**-1.53 [-4.21, 1.14]**
Heterogeneity: Tau² = 6.71; Chi² = 20.23, df = 4 (P = 0.0004); I² = 80%								
Test for overall effect: Z = 1.12 (P = 0.26)								
**1.8.2 2-year**								
DIBASY 2012	-10.4	12.2	38	-8.7	15.2	18	4.0%	-1.70 [-9.72, 6.32]
Dixon 2008	-0.7	11.1	30	-0.9	11.1	30	6.0%	0.20 [-5.42, 5.82]
Dixon 2012	-1.1	6	30	-3.5	7	30	8.7%	2.40 [-0.90, 5.70]
O'Brien 2006	-9.1	10.4	39	-1.3	9.2	31	7.1%	-7.80 [-12.40, -3.20]
O'Brien 2010	-6	9.4	24	-6.9	12.5	18	4.8%	0.90 [-5.99, 7.79]
TRAMOMTANA 2015	-1.2	12.5	23	-0.6	14.3	47	5.1%	-0.60 [-7.14, 5.94]
**Subtotal (95% CI)**			**184**			**174**	**35.7%**	**-1.10 [-4.70, 2.50]**
Heterogeneity: Tau² = 11.79; Chi² = 12.92, df = 5 (P = 0.02); I² = 61%								
Test for overall effect: Z = 0.60 (P = 0.55)								
**1.8.3 3-year or more**								
DIBASY 2015	-10.4	12.7	38	-13.3	19	15	2.8%	2.90 [-7.53, 13.33]
STAMPEDE 2014	-5.3	12	97	-6.5	12.3	40	7.2%	1.20 [-3.30, 5.70]
TRIABETES 2015	-1.1	4.4	38	-2.9	2	14	10.5%	1.80 [0.05, 3.55]
**Subtotal (95% CI)**			**173**			**69**	**20.5%**	**1.75 [0.14, 3.36]**
Heterogeneity: Tau² = 0.00; Chi² = 0.11, df = 2 (P = 0.95); I² = 0%								
Test for overall effect: Z = 2.13 (P = 0.03)								
**Total (95% CI)**			**619**			**432**	**100.0%**	**-0.68 [-2.66, 1.31]**
Heterogeneity: Tau² = 8.92; Chi² = 58.02, df = 13 (P < 0.00001); I² = 78%								
Test for overall effect: Z = 0.67 (P = 0.50)								
Test for subgroup differences: Chi² = 5.25, df = 2 (P = 0.07), I² = 61.9%								

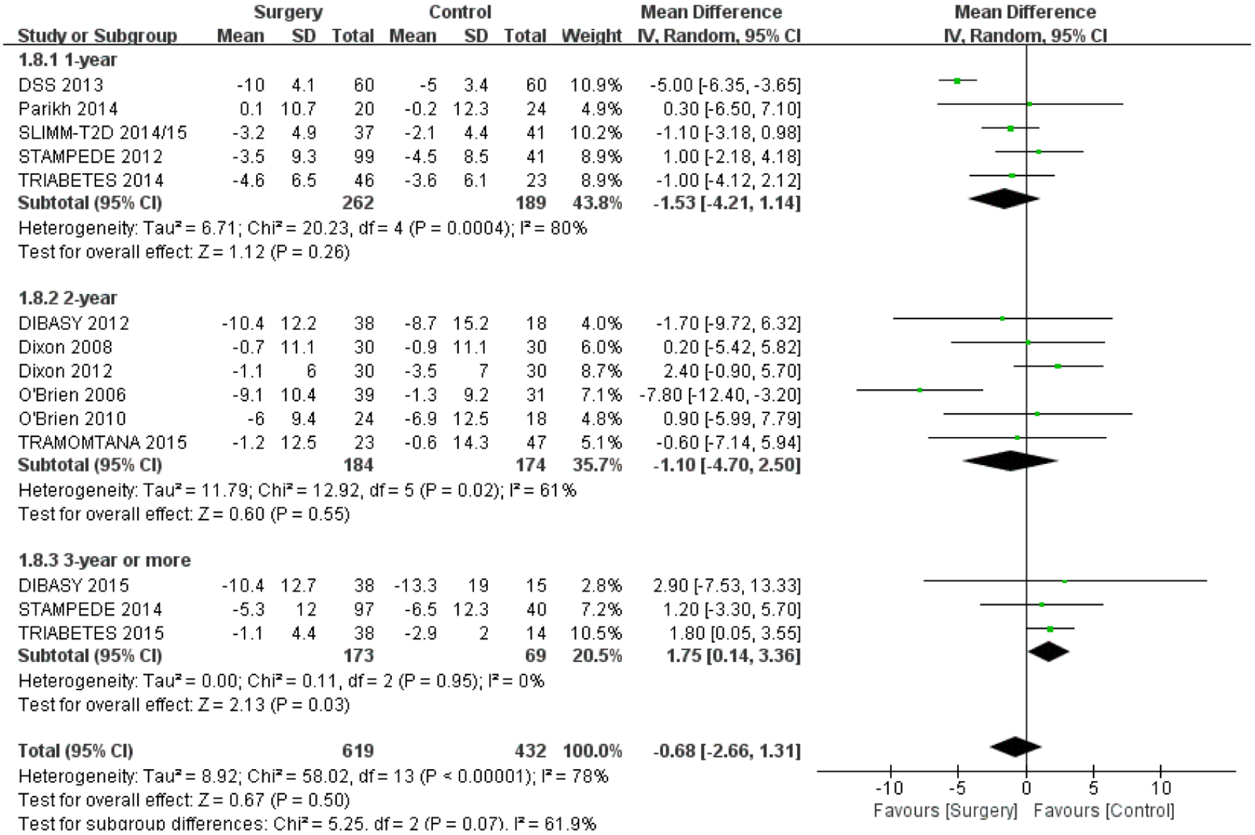

Figure S19.The forest plot of diastolic pressure (mmHg) in terms of follow-up duration

Study or Subgroup	Surgery Mean	Surgery SD	Surgery Total	Control Mean	Control SD	Control Total	Weight	Mean Difference IV, Random, 95% CI
**2.8.1 Sleeve gastrectomy**								
STAMPEDE 2012	-3.6	9.6	49	-4.5	8.5	41	5.7%	0.90 [-2.84, 4.64]
STAMPEDE 2014	-6.3	13.3	49	-6.5	12.3	40	4.8%	0.20 [-5.13, 5.53]
**Subtotal (95% CI)**			**98**			**81**	**10.5%**	**0.67 [-2.39, 3.73]**
Heterogeneity: Tau² = 0.00; Chi² = 0.04, df = 1 (P = 0.83); I² = 0%								
Test for overall effect: Z = 0.43 (P = 0.67)								
**2.8.2 Roux-en-Y gastric bypass**								
DIBASY 2012	-7.3	12.5	19	-8.7	15.2	18	3.1%	1.40 [-7.59, 10.39]
DIBASY 2015	-8.3	13.5	19	-13.3	19	15	2.3%	5.00 [-6.37, 16.37]
DSS 2013	-10	4.1	60	-5	3.4	60	6.7%	-5.00 [-6.35, -3.65]
SLIMM-T2D 2014	-5.1	4.7	19	-2.1	4.8	19	6.0%	-3.00 [-6.02, 0.02]
STAMPEDE 2012	-3.5	9	50	-4.5	8.5	41	5.7%	1.00 [-2.60, 4.60]
STAMPEDE 2014	-4.3	10.6	48	-6.5	12.3	40	5.1%	2.20 [-2.65, 7.05]
TRIABETES 2014	-7.4	6.1	24	-3.6	6.1	23	5.8%	-3.80 [-7.29, -0.31]
TRIABETES 2015	-5.4	1.8	18	-2.9	2	14	6.7%	-2.50 [-3.84, -1.16]
**Subtotal (95% CI)**			**257**			**230**	**41.4%**	**-2.09 [-3.95, -0.22]**
Heterogeneity: Tau² = 3.66; Chi² = 21.01, df = 7 (P = 0.004); I² = 67%								
Test for overall effect: Z = 2.19 (P = 0.03)								
**2.8.3 Laparoscopic adjustable gastric banding**								
Dixon 2008	-0.7	11.1	30	-0.9	11.1	30	4.6%	0.20 [-5.42, 5.82]
Dixon 2012	-1.1	6	30	-3.5	7	30	5.9%	2.40 [-0.90, 5.70]
O'Brien 2006	-9.1	10.4	39	-1.3	9.2	31	5.2%	-7.80 [-12.40, -3.20]
O'Brien 2010	-6	9.4	24	-6.9	12.5	18	4.0%	0.90 [-5.99, 7.79]
SLIMM-T2D 2015	-1.2	4.4	18	-2.1	4.2	22	6.2%	0.90 [-1.79, 3.59]
TRIABETES 2014	-1.5	5.5	22	-3.6	6.1	23	5.8%	2.10 [-1.29, 5.49]
TRIABETES 2015	2.7	1.7	20	-2.9	2	14	6.7%	5.60 [4.31, 6.89]
**Subtotal (95% CI)**			**183**			**168**	**38.4%**	**0.92 [-2.25, 4.09]**
Heterogeneity: Tau² = 14.13; Chi² = 40.01, df = 6 (P < 0.00001); I² = 85%								
Test for overall effect: Z = 0.57 (P = 0.57)								
**2.8.4 Biliopancreatic diversion**								
DIBASY 2012	-13.5	11.4	19	-8.7	15.2	18	3.2%	-4.80 [-13.49, 3.89]
DIBASY 2015	-12.4	11.9	19	-13.3	19	15	2.4%	0.90 [-10.10, 11.90]
TRAMOMTANA 2015	-1.2	12.5	23	-0.6	14.3	47	4.2%	-0.60 [-7.14, 5.94]
**Subtotal (95% CI)**			**61**			**80**	**9.8%**	**-1.56 [-6.28, 3.16]**
Heterogeneity: Tau² = 0.00; Chi² = 0.81, df = 2 (P = 0.67); I² = 0%								
Test for overall effect: Z = 0.65 (P = 0.52)								
**Total (95% CI)**			**599**			**559**	**100.0%**	**-0.34 [-2.46, 1.79]**
Heterogeneity: Tau² = 17.13; Chi² = 164.53, df = 19 (P < 0.00001); I² = 88%								
Test for overall effect: Z = 0.31 (P = 0.76)								
Test for subgroup differences: Chi² = 3.90, df = 3 (P = 0.27), I² = 23.1%								

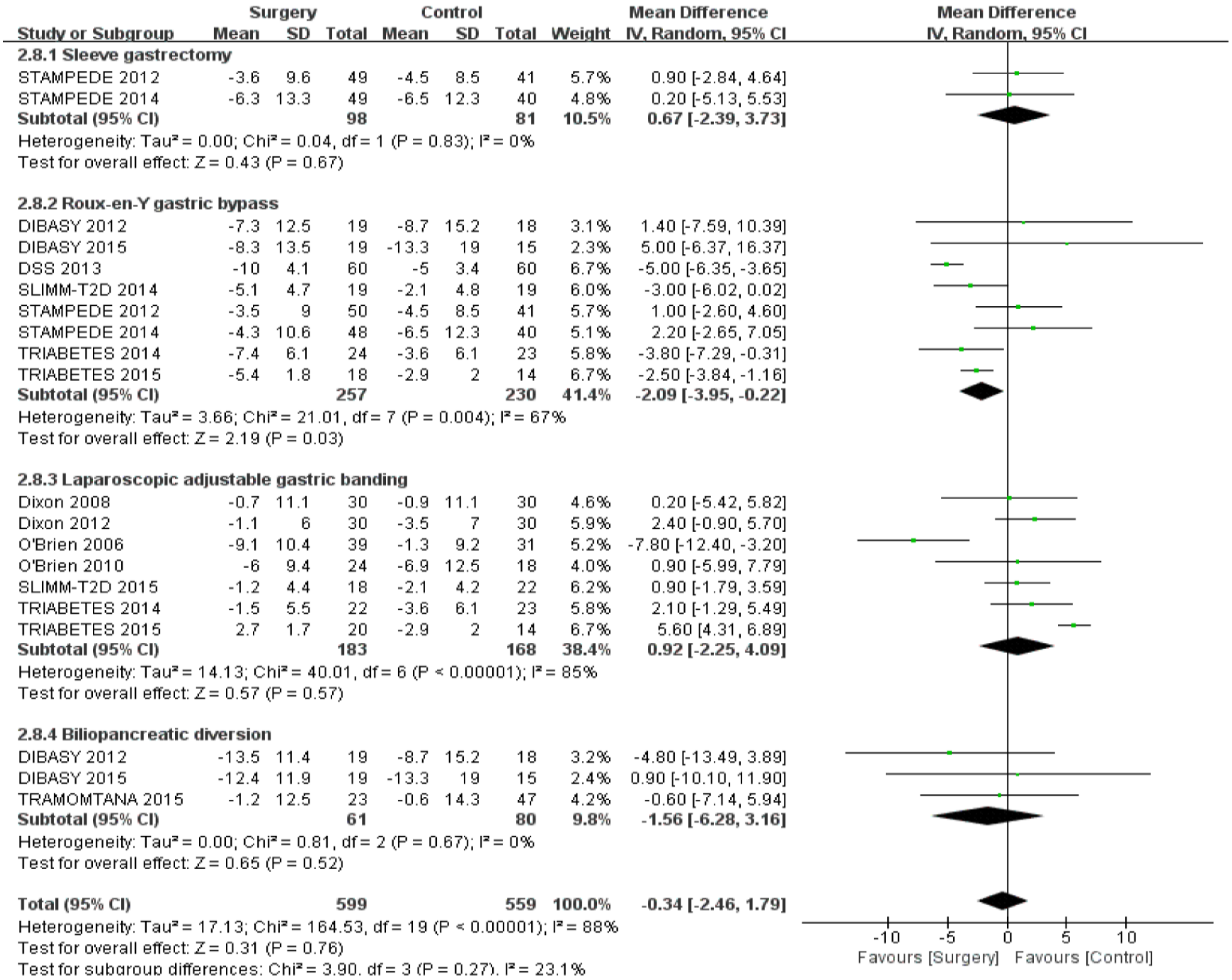

Figure S20.The forest plot of diastolic pressure (mmHg) in terms of surgical techniques

Study or Subgroup	Surgery Mean	Surgery SD	Surgery Total	Control Mean	Control SD	Control Total	Weight	Mean Difference IV, Random, 95% CI
**3.8.1 Nonsevere obesity**								
DSS 2013	-10	4.1	60	-5	3.4	60	10.9%	-5.00 [-6.35, -3.65]
O'Brien 2006	-9.1	10.4	39	-1.3	9.2	31	7.1%	-7.80 [-12.40, -3.20]
Parikh 2014	0.1	10.7	20	-0.2	12.3	24	4.9%	0.30 [-6.50, 7.10]
TRIABETES 2014	-4.6	6.5	46	-3.6	6.1	23	8.9%	-1.00 [-4.12, 2.12]
TRIABETES 2015	-1.1	4.4	38	-2.9	2	14	10.5%	1.80 [0.05, 3.55]
**Subtotal (95% CI)**			**203**			**152**	**42.4%**	**-2.35 [-6.20, 1.50]**
Heterogeneity: Tau² = 15.76; Chi² = 43.11, df = 4 (P < 0.00001); I² = 91%								
Test for overall effect: Z = 1.20 (P = 0.23)								
**3.8.2 Severe obesity**								
DIBASY 2012	-10.4	12.2	38	-8.7	15.2	18	4.0%	-1.70 [-9.72, 6.32]
DIBASY 2015	-10.4	12.7	38	-13.3	19	15	2.8%	2.90 [-7.53, 13.33]
Dixon 2008	-0.7	11.1	30	-0.9	11.1	30	6.0%	0.20 [-5.42, 5.82]
Dixon 2012	-1.1	6	30	-3.5	7	30	8.7%	2.40 [-0.90, 5.70]
O'Brien 2010	-6	9.4	24	-6.9	12.5	18	4.8%	0.90 [-5.99, 7.79]
SLIMM-T2D 2014/15	-3.2	4.9	37	-2.1	4.4	41	10.2%	-1.10 [-3.18, 0.98]
STAMPEDE 2012	-3.5	9.3	99	-4.5	8.5	41	8.9%	1.00 [-2.18, 4.18]
STAMPEDE 2014	-5.3	12	97	-6.5	12.3	40	7.2%	1.20 [-3.30, 5.70]
TRAMOMTANA 2015	-1.2	12.5	23	-0.6	14.3	47	5.1%	-0.60 [-7.14, 5.94]
**Subtotal (95% CI)**			**416**			**280**	**57.6%**	**0.24 [-1.09, 1.56]**
Heterogeneity: Tau² = 0.00; Chi² = 4.22, df = 8 (P = 0.84); I² = 0%								
Test for overall effect: Z = 0.35 (P = 0.73)								
**Total (95% CI)**			**619**			**432**	**100.0%**	**-0.68 [-2.66, 1.31]**
Heterogeneity: Tau² = 8.92; Chi² = 58.02, df = 13 (P < 0.00001); I² = 78%								
Test for overall effect: Z = 0.67 (P = 0.50)								
Test for subgroup differences: Chi² = 1.55, df = 1 (P = 0.21), I² = 35.5%								

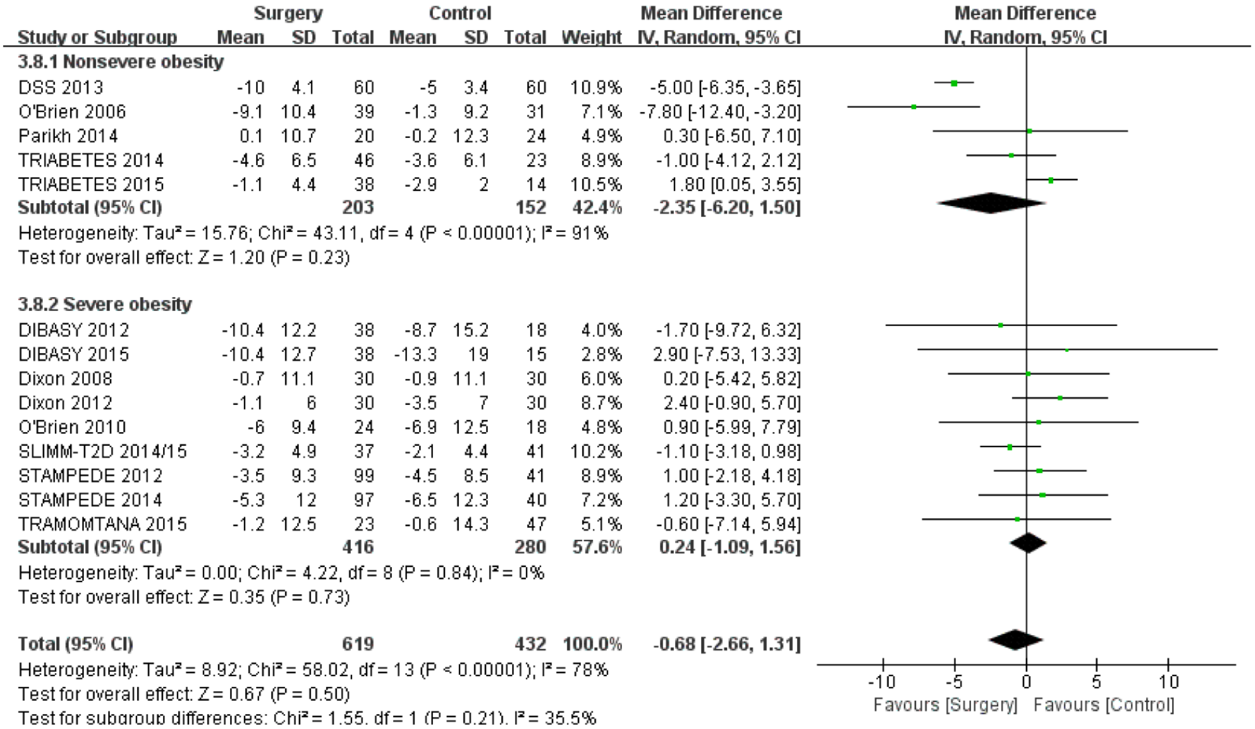

Figure S21.The forest plot of diastolic pressure (mmHg) in terms of obesity levels

Study or Subgroup	Surgery Mean	Surgery SD	Surgery Total	Control Mean	Control SD	Control Total	Weight	Mean Difference IV, Random, 95% CI
**1.9.1 1-year**								
DSS 2013	-1.7	1.5	60	-0.8	0.8	60	5.5%	-0.90 [-1.33, -0.47]
Liang 2013	-1.8	1.1	31	-0.4	1.3	70	4.8%	-1.40 [-1.89, -0.91]
Mingrone 2002	-0.9	0.7	46	0	0.7	33	6.8%	-0.90 [-1.21, -0.59]
Parikh 2014	-0.6	1.1	20	-0.2	0.9	24	3.9%	-0.40 [-1.00, 0.20]
SLIMM-T2D 2014/15	-0.4	0.4	37	-0.3	0.5	41	8.1%	-0.10 [-0.30, 0.10]
STAMPEDE 2012	-0.8	1	99	-0.3	0.6	41	7.3%	-0.50 [-0.77, -0.23]
TRIABETES 2014	-1.1	0.9	46	-0.4	0.9	23	5.3%	-0.70 [-1.15, -0.25]
**Subtotal (95% CI)**			**339**			**292**	**41.6%**	**-0.68 [-1.02, -0.34]**
Heterogeneity: Tau² = 0.17; Chi² = 38.61, df = 6 (P < 0.00001); I² = 84%								
Test for overall effect: Z = 3.96 (P < 0.0001)								
**1.9.2 2-year**								
DIBASY 2012	-1	1.1	38	-0.6	0.7	18	5.0%	-0.40 [-0.88, 0.08]
Dixon 2008	-0.8	1.1	30	0	1.4	30	3.7%	-0.80 [-1.44, -0.16]
Dixon 2012	-0.4	0.5	30	-0.1	0.4	30	7.8%	-0.30 [-0.53, -0.07]
O'Brien 2006	-0.3	0.5	39	-0.1	0.6	31	7.4%	-0.20 [-0.46, 0.06]
O'Brien 2010	-0.6	0.4	24	-0.4	0.9	18	5.3%	-0.20 [-0.65, 0.25]
TRAMOMTANA 2015	-0.2	0.4	23	0	0.3	47	8.2%	-0.20 [-0.38, -0.02]
**Subtotal (95% CI)**			**184**			**174**	**37.3%**	**-0.26 [-0.37, -0.14]**
Heterogeneity: Tau² = 0.00; Chi² = 3.88, df = 5 (P = 0.57); I² = 0%								
Test for overall effect: Z = 4.36 (P < 0.0001)								
**1.9.3 3-year or more**								
DIBASY 2015	-0.8	0.9	38	-0.7	0.5	15	6.0%	-0.10 [-0.48, 0.28]
STAMPEDE 2014	-0.7	0.8	97	-0.5	1	40	6.4%	-0.20 [-0.55, 0.15]
TRIABETES 2015	-0.8	0.3	38	-0.2	0.2	14	8.7%	-0.60 [-0.74, -0.46]
**Subtotal (95% CI)**			**173**			**69**	**21.0%**	**-0.33 [-0.68, 0.02]**
Heterogeneity: Tau² = 0.07; Chi² = 8.97, df = 2 (P = 0.01); I² = 78%								
Test for overall effect: Z = 1.87 (P = 0.06)								
**Total (95% CI)**			**696**			**535**	**100.0%**	**-0.46 [-0.62, -0.30]**
Heterogeneity: Tau² = 0.07; Chi² = 62.58, df = 15 (P < 0.00001); I² = 76%								
Test for overall effect: Z = 5.77 (P < 0.00001)								
Test for subgroup differences: Chi² = 5.49, df = 2 (P = 0.06), I² = 63.5%								

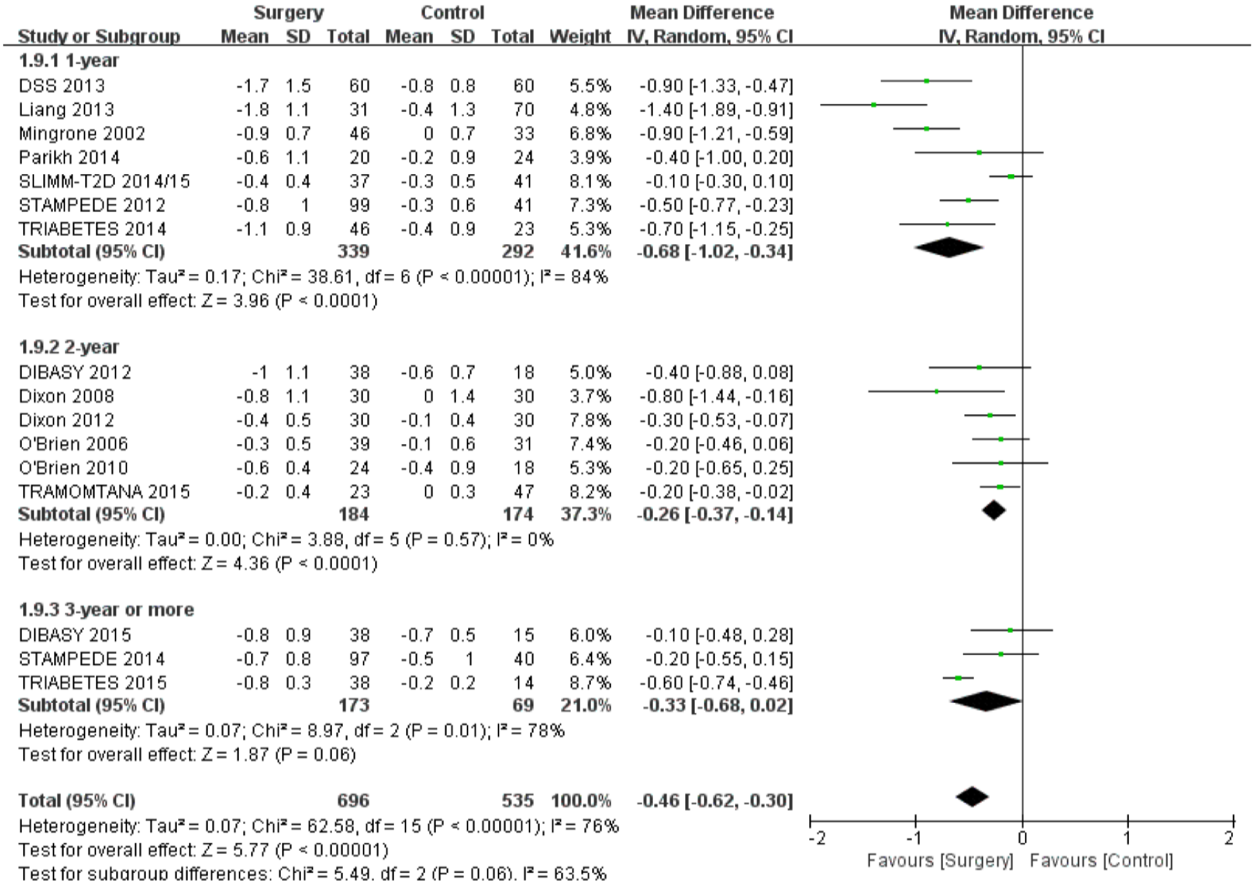

Figure S22.The forest plot of triglycerides (mmol/L) in terms of follow-up duration

Study or Subgroup	Surgery Mean	Surgery SD	Surgery Total	Control Mean	Control SD	Control Total	Weight	Mean Difference IV, Random, 95% CI
**2.9.1 Sleeve gastrectomy**								
STAMPEDE 2012	-0.7	1	49	-0.3	0.6	41	4.8%	-0.40 [-0.73, -0.07]
STAMPEDE 2014	-0.6	0.7	49	-0.5	1	40	4.6%	-0.10 [-0.47, 0.27]
**Subtotal (95% CI)**			**98**			**81**	**9.4%**	**-0.26 [-0.55, 0.03]**
Heterogeneity: Tau² = 0.01; Chi² = 1.40, df = 1 (P = 0.24); I² = 29%								
Test for overall effect: Z = 1.73 (P = 0.08)								
**2.9.2 Roux-en-Y gastric bypass**								
DIBASY 2012	-0.5	0.7	19	-0.6	0.7	18	4.2%	0.10 [-0.35, 0.55]
DIBASY 2015	-0.4	0.8	19	-0.7	0.5	15	4.3%	0.30 [-0.14, 0.74]
DSS 2013	-1.7	1.5	60	-0.8	0.8	60	4.3%	-0.90 [-1.33, -0.47]
Liang 2013	-1.8	1.1	31	-0.4	1.3	70	4.0%	-1.40 [-1.89, -0.91]
SLIMM-T2D 2014	-0.5	0.3	19	-0.1	0.3	19	5.4%	-0.40 [-0.59, -0.21]
STAMPEDE 2012	-0.8	1	50	-0.3	0.6	41	4.8%	-0.50 [-0.83, -0.17]
STAMPEDE 2014	-0.9	1	48	-0.5	1	40	4.4%	-0.40 [-0.82, 0.02]
TRIABETES 2014	-0.9	0.9	24	-0.4	0.9	23	3.9%	-0.50 [-1.01, 0.01]
TRIABETES 2015	-1.1	0.2	18	-0.2	0.2	14	5.5%	-0.90 [-1.04, -0.76]
**Subtotal (95% CI)**			**288**			**300**	**40.6%**	**-0.52 [-0.80, -0.23]**
Heterogeneity: Tau² = 0.16; Chi² = 60.03, df = 8 (P < 0.00001); I² = 87%								
Test for overall effect: Z = 3.49 (P = 0.0005)								
**2.9.3 Laparoscopic adjustable gastric banding**								
Dixon 2008	-0.8	1.1	30	0	1.4	30	3.3%	-0.80 [-1.44, -0.16]
Dixon 2012	-0.4	0.5	30	-0.1	0.4	30	5.2%	-0.30 [-0.53, -0.07]
O'Brien 2006	-0.3	0.5	39	-0.1	0.6	31	5.1%	-0.20 [-0.46, 0.06]
O'Brien 2010	-0.6	0.4	24	-0.4	0.9	18	4.2%	-0.20 [-0.65, 0.25]
SLIMM-T2D 2015	-0.3	0.5	18	-0.5	0.5	22	4.9%	0.20 [-0.11, 0.51]
TRIABETES 2014	-1.2	0.9	22	-0.4	0.9	23	3.8%	-0.80 [-1.33, -0.27]
TRIABETES 2015	-0.6	0.2	20	-0.2	0.2	14	5.5%	-0.40 [-0.54, -0.26]
**Subtotal (95% CI)**			**183**			**168**	**32.1%**	**-0.30 [-0.50, -0.10]**
Heterogeneity: Tau² = 0.04; Chi² = 18.48, df = 6 (P = 0.005); I² = 68%								
Test for overall effect: Z = 2.91 (P = 0.004)								
**2.9.4 Biliopancreatic diversion**								
DIBASY 2012	-1.5	1.1	19	-0.6	0.7	18	3.5%	-0.90 [-1.49, -0.31]
DIBASY 2015	-1.2	0.9	19	-0.7	0.5	15	4.1%	-0.50 [-0.98, -0.02]
Mingrone 2002	-0.9	0.7	46	0	0.7	33	4.9%	-0.90 [-1.21, -0.59]
TRAMOMTANA 2015	0	0.3	23	-0.2	0.4	47	5.4%	0.20 [0.03, 0.37]
**Subtotal (95% CI)**			**107**			**113**	**17.9%**	**-0.51 [-1.18, 0.17]**
Heterogeneity: Tau² = 0.43; Chi² = 46.96, df = 3 (P < 0.00001); I² = 94%								
Test for overall effect: Z = 1.47 (P = 0.14)								
**Total (95% CI)**			**676**			**662**	**100.0%**	**-0.42 [-0.60, -0.24]**
Heterogeneity: Tau² = 0.15; Chi² = 171.36, df = 21 (P < 0.00001); I² = 88%								
Test for overall effect: Z = 4.60 (P < 0.00001)								
Test for subgroup differences: Chi² = 2.08, df = 3 (P = 0.56), I² = 0%								

Mean Difference
IV, Random, 95% CI

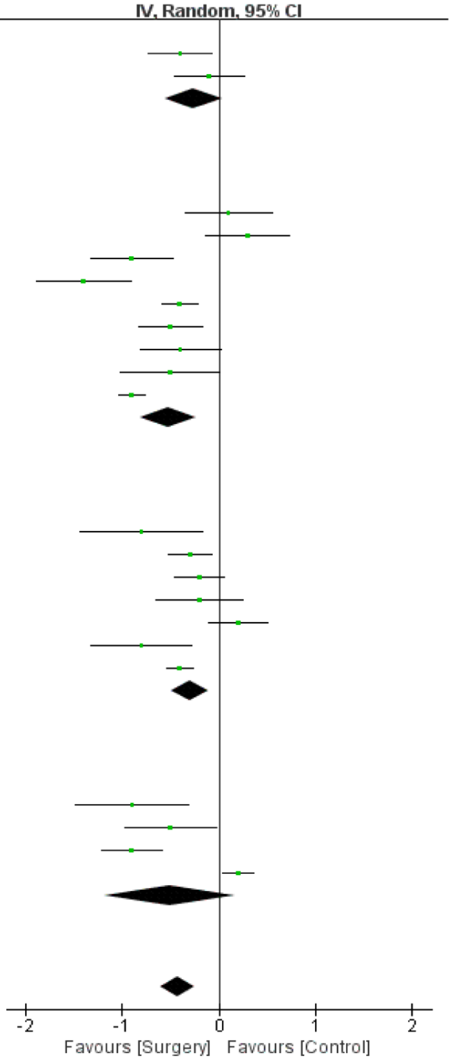

Figure S23.The forest plot of triglycerides (mmol/L) in terms of surgical techniques

Study or Subgroup	Surgery Mean	Surgery SD	Surgery Total	Control Mean	Control SD	Control Total	Weight	Mean Difference IV, Random, 95% CI
**3.9.1 Nonsevere obesity**								
DSS 2013	-1.7	1.5	60	-0.8	0.8	60	5.5%	-0.90 [-1.33, -0.47]
Liang 2013	-1.8	1.1	31	-0.4	1.3	70	4.8%	-1.40 [-1.89, -0.91]
O'Brien 2006	-0.3	0.5	39	-0.1	0.6	31	7.4%	-0.20 [-0.46, 0.06]
Parikh 2014	-0.6	1.1	20	-0.2	0.9	24	3.9%	-0.40 [-1.00, 0.20]
TRIABETES 2014	-1.1	0.9	46	-0.4	0.9	23	5.3%	-0.70 [-1.15, -0.25]
TRIABETES 2015	-0.8	0.3	38	-0.2	0.2	14	8.7%	-0.60 [-0.74, -0.46]
**Subtotal (95% CI)**			**234**			**222**	**35.5%**	**-0.67 [-0.96, -0.38]**
Heterogeneity: Tau² = 0.09; Chi² = 21.45, df = 5 (P = 0.0007); I² = 77%								
Test for overall effect: Z = 4.55 (P < 0.00001)								
**3.9.2 Severe obesity**								
DIBASY 2012	-1	1.1	38	-0.6	0.7	18	5.0%	-0.40 [-0.88, 0.08]
DIBASY 2015	-0.8	0.9	38	-0.7	0.5	15	6.0%	-0.10 [-0.48, 0.28]
Dixon 2008	-0.8	1.1	30	0	1.4	30	3.7%	-0.80 [-1.44, -0.16]
Dixon 2012	-0.4	0.5	30	-0.1	0.4	30	7.8%	-0.30 [-0.53, -0.07]
Mingrone 2002	-0.9	0.7	46	0	0.7	33	6.8%	-0.90 [-1.21, -0.59]
O'Brien 2010	-0.6	0.4	24	-0.4	0.9	18	5.3%	-0.20 [-0.65, 0.25]
SLIMM-T2D 2014/15	-0.4	0.4	37	-0.3	0.5	41	8.1%	-0.10 [-0.30, 0.10]
STAMPEDE 2012	-0.8	1	99	-0.3	0.6	41	7.3%	-0.50 [-0.77, -0.23]
STAMPEDE 2014	-0.7	0.8	97	-0.5	1	40	6.4%	-0.20 [-0.55, 0.15]
TRAMOMTANA 2015	-0.2	0.4	23	0	0.3	47	8.2%	-0.20 [-0.38, -0.02]
**Subtotal (95% CI)**			**462**			**313**	**64.5%**	**-0.34 [-0.50, -0.18]**
Heterogeneity: Tau² = 0.04; Chi² = 25.30, df = 9 (P = 0.003); I² = 64%								
Test for overall effect: Z = 4.11 (P < 0.0001)								
**Total (95% CI)**			**696**			**535**	**100.0%**	**-0.46 [-0.62, -0.30]**
Heterogeneity: Tau² = 0.07; Chi² = 62.58, df = 15 (P < 0.00001); I² = 76%								
Test for overall effect: Z = 5.77 (P < 0.00001)								
Test for subgroup differences: Chi² = 3.83, df = 1 (P = 0.05), I² = 73.9%								

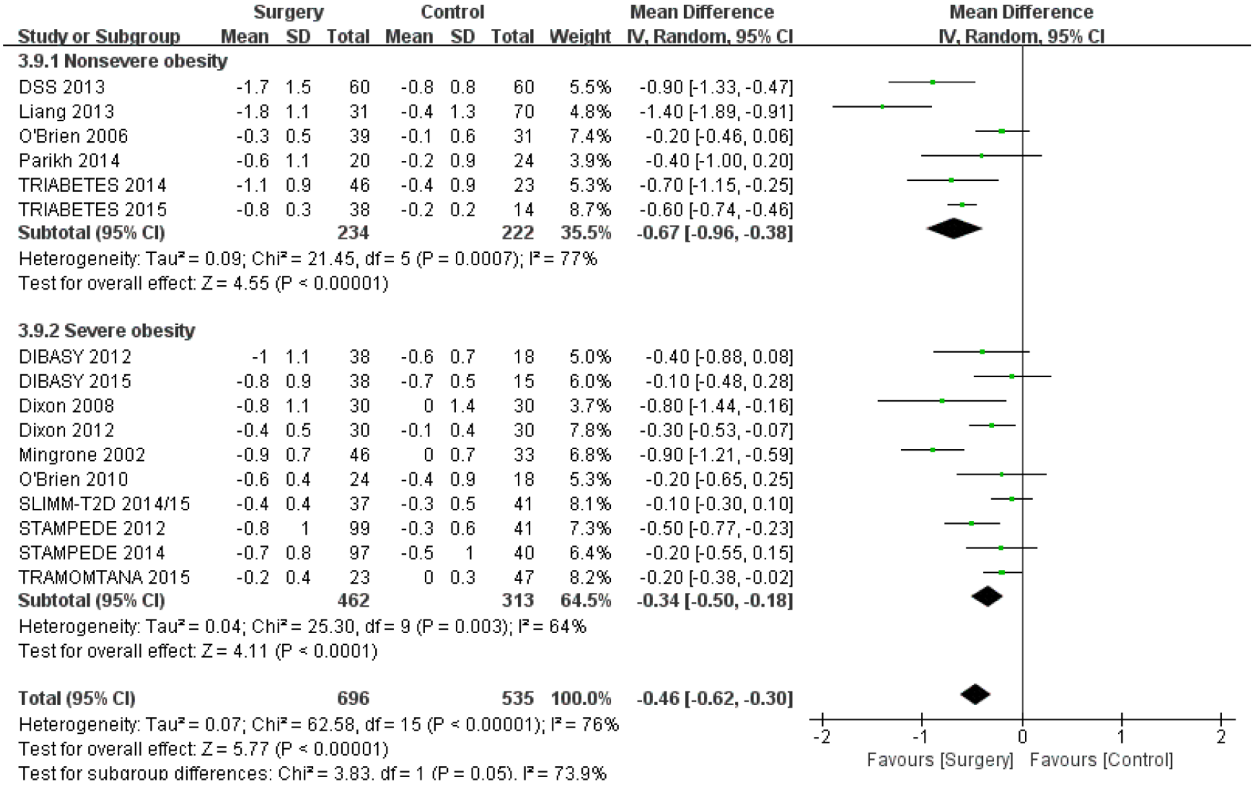

Figure S24.The forest plot of triglycerides (mmol/L) in terms of obesity levels

Study or Subgroup	Surgery Mean	Surgery SD	Surgery Total	Control Mean	Control SD	Control Total	Weight	Mean Difference IV, Random, 95% CI
**1.10.1 1-year**								
DSS 2013	-0.7	0.4	60	-0.7	0.4	60	8.7%	0.00 [-0.14, 0.14]
Liang 2013	-2.1	0.5	31	-0.7	0.9	70	8.1%	-1.40 [-1.67, -1.13]
Mingrone 2002	-1.2	1	46	0	1.1	33	6.9%	-1.20 [-1.67, -0.73]
Parikh 2014	0.3	1.4	20	-0.5	1.5	24	4.6%	0.80 [-0.06, 1.66]
SLIMM-T2D 2014/15	-0.1	0.5	37	0.1	0.5	41	8.4%	-0.20 [-0.42, 0.02]
STAMPEDE 2012	-0.1	1.2	99	-0.1	1.1	41	7.3%	0.00 [-0.41, 0.41]
TRIABETES 2014	-0.6	0.8	46	-0.6	0.8	23	7.4%	0.00 [-0.40, 0.40]
**Subtotal (95% CI)**			**339**			**292**	**51.4%**	**-0.33 [-0.80, 0.15]**
Heterogeneity: Tau² = 0.36; Chi² = 103.26, df = 6 (P < 0.00001); I² = 94%								
Test for overall effect: Z = 1.35 (P = 0.18)								
**1.10.2 2-year**								
DIBASY 2012	-1.6	1.6	38	-1.2	1.3	18	5.0%	-0.40 [-1.19, 0.39]
Dixon 2008	0.1	1.3	30	0	0.8	30	6.4%	0.10 [-0.45, 0.65]
Dixon 2012	0.1	0.4	30	0.4	0.6	30	8.2%	-0.30 [-0.56, -0.04]
O'Brien 2006	0	1	39	-0.2	0.9	31	7.1%	0.20 [-0.25, 0.65]
TRAMOMTANA 2015	-0.3	0.5	23	0	0.3	47	8.4%	-0.30 [-0.52, -0.08]
**Subtotal (95% CI)**			**160**			**156**	**35.1%**	**-0.19 [-0.38, 0.01]**
Heterogeneity: Tau² = 0.02; Chi² = 5.80, df = 4 (P = 0.21); I² = 31%								
Test for overall effect: Z = 1.83 (P = 0.07)								
**1.10.3 3-year or more**								
DIBASY 2015	-1.5	1.8	38	-1.6	1.1	15	4.9%	0.10 [-0.70, 0.90]
TRIABETES 2015	-0.1	0.3	38	-0.3	0.3	14	8.6%	0.20 [0.02, 0.38]
**Subtotal (95% CI)**			**76**			**29**	**13.4%**	**0.19 [0.02, 0.37]**
Heterogeneity: Tau² = 0.00; Chi² = 0.06, df = 1 (P = 0.81); I² = 0%								
Test for overall effect: Z = 2.13 (P = 0.03)								
**Total (95% CI)**			**575**			**477**	**100.0%**	**-0.20 [-0.47, 0.06]**
Heterogeneity: Tau² = 0.20; Chi² = 129.16, df = 13 (P < 0.00001); I² = 90%								
Test for overall effect: Z = 1.53 (P = 0.13)								
Test for subgroup differences: Chi² = 9.72, df = 2 (P = 0.008), I² = 79.4%								

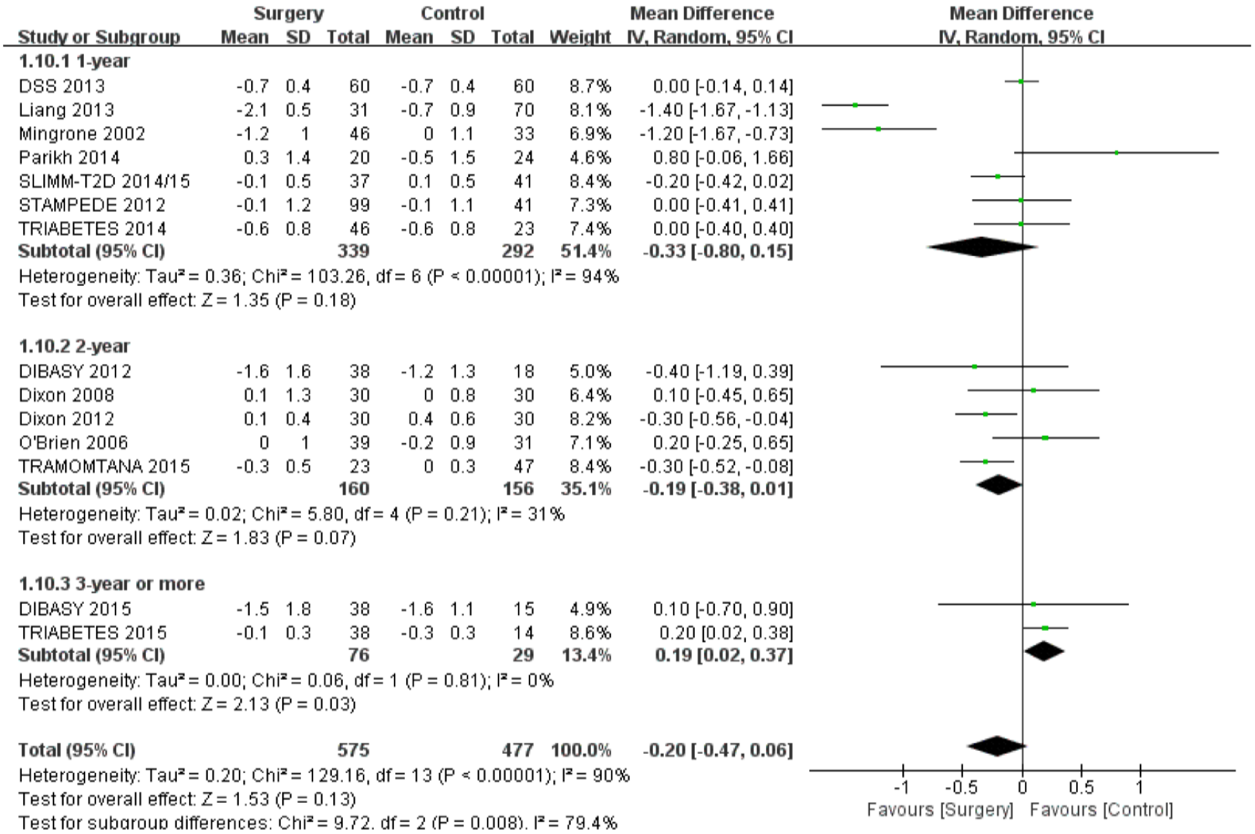

Figure S25.The forest plot of total cholesterol (mmol/L) in terms of follow-up duration

Study or Subgroup	Surgery Mean	Surgery SD	Surgery Total	Control Mean	Control SD	Control Total	Weight	Mean Difference IV, Random, 95% CI
**2.10.1 Sleeve gastrectomy**								
STAMPEDE 2012	0	1.1	49	-0.1	1.1	41	5.3%	0.10 [-0.36, 0.56]
**Subtotal (95% CI)**			**49**			**41**	**5.3%**	**0.10 [-0.36, 0.56]**
Heterogeneity: Not applicable								
Test for overall effect: Z = 0.43 (P = 0.67)								
**2.10.2 Roux-en-Y gastric bypass**								
DIBASY 2012	-0.4	0.8	19	-1.2	1.3	18	4.2%	0.80 [0.10, 1.50]
DIBASY 2015	-0.3	1.3	19	-1.6	1.1	15	3.8%	1.30 [0.49, 2.11]
DSS 2013	-0.7	0.4	60	-0.7	0.4	60	6.3%	0.00 [-0.14, 0.14]
Liang 2013	-2.1	0.5	31	-0.7	0.9	70	6.0%	-1.40 [-1.67, -1.13]
SLIMM-T2D 2014	-0.1	0.6	19	0.2	0.6	19	5.6%	-0.30 [-0.68, 0.08]
STAMPEDE 2012	-0.2	1.2	50	-0.1	1.1	41	5.2%	-0.10 [-0.57, 0.37]
TRIABETES 2014	-0.8	0.8	24	-0.6	0.8	23	5.3%	-0.20 [-0.66, 0.26]
TRIABETES 2015	-0.3	0.2	18	-0.3	0.3	14	6.3%	0.00 [-0.18, 0.18]
**Subtotal (95% CI)**			**240**			**260**	**42.6%**	**-0.06 [-0.49, 0.36]**
Heterogeneity: Tau² = 0.32; Chi² = 106.46, df = 7 (P < 0.00001); I² = 93%								
Test for overall effect: Z = 0.28 (P = 0.78)								
**2.10.3 Laparoscopic adjustable gastric banding**								
Dixon 2008	0.1	1.3	30	0	0.8	30	4.9%	0.10 [-0.45, 0.65]
Dixon 2012	0.1	0.4	30	0.4	0.6	30	6.0%	-0.30 [-0.56, -0.04]
O'Brien 2006	0	1	39	-0.2	0.9	31	5.3%	0.20 [-0.25, 0.65]
SLIMM-T2D 2015	-0.1	0.5	18	0.1	0.5	22	5.9%	-0.20 [-0.51, 0.11]
TRIABETES 2014	-0.3	0.8	22	-0.6	0.8	23	5.2%	0.30 [-0.17, 0.77]
TRIABETES 2015	0.1	0.2	20	-0.3	0.3	14	6.3%	0.40 [0.22, 0.58]
**Subtotal (95% CI)**			**159**			**150**	**33.6%**	**0.08 [-0.22, 0.37]**
Heterogeneity: Tau² = 0.10; Chi² = 24.23, df = 5 (P = 0.0002); I² = 79%								
Test for overall effect: Z = 0.50 (P = 0.62)								
**2.10.4 Biliopancreatic diversion**								
DIBASY 2012	-2.8	1.3	19	-1.2	1.3	18	3.6%	-1.60 [-2.44, -0.76]
DIBASY 2015	-2.6	1.4	19	-1.6	1.1	15	3.6%	-1.00 [-1.84, -0.16]
Mingrone 2002	-1.2	1	46	0	1.1	33	5.2%	-1.20 [-1.67, -0.73]
TRAMOMTANA 2015	-0.3	0.5	23	0	0.3	47	6.2%	-0.30 [-0.52, -0.08]
**Subtotal (95% CI)**			**107**			**113**	**18.6%**	**-0.97 [-1.63, -0.30]**
Heterogeneity: Tau² = 0.36; Chi² = 19.25, df = 3 (P = 0.0002); I² = 84%								
Test for overall effect: Z = 2.86 (P = 0.004)								
**Total (95% CI)**			**555**			**564**	**100.0%**	**-0.18 [-0.42, 0.06]**
Heterogeneity: Tau² = 0.23; Chi² = 186.78, df = 18 (P < 0.00001); I² = 90%								
Test for overall effect: Z = 1.44 (P = 0.15)								
Test for subgroup differences: Chi² = 8.48, df = 3 (P = 0.04), I² = 64.6%								

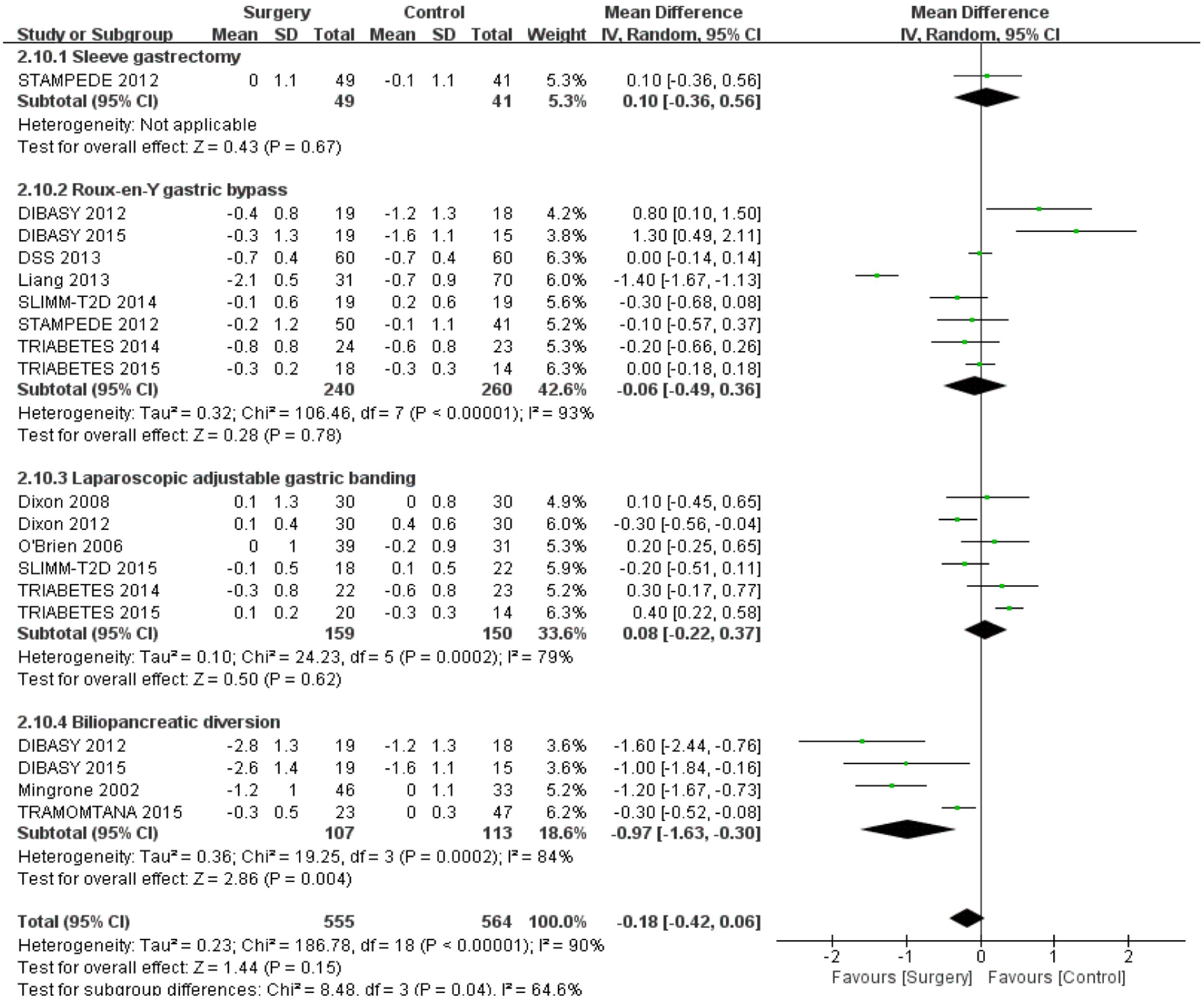

Figure S26.The forest plot of total cholesterol (mmol/L) in terms of surgical techniques

Study or Subgroup	Surgery Mean	Surgery SD	Surgery Total	Control Mean	Control SD	Control Total	Weight	Mean Difference IV, Random, 95% CI
**3.10.1 Nonsevere obesity**								
DSS 2013	-0.7	0.4	60	-0.7	0.4	60	8.7%	0.00 [-0.14, 0.14]
Liang 2013	-2.1	0.5	31	-0.7	0.9	70	8.1%	-1.40 [-1.67, -1.13]
O'Brien 2006	0	1	39	-0.2	0.9	31	7.1%	0.20 [-0.25, 0.65]
Parikh 2014	0.3	1.4	20	-0.5	1.5	24	4.6%	0.80 [-0.06, 1.66]
TRIABETES 2014	-0.6	0.8	46	-0.6	0.8	23	7.4%	0.00 [-0.40, 0.40]
TRIABETES 2015	-0.1	0.3	38	-0.3	0.3	14	8.6%	0.20 [0.02, 0.38]
**Subtotal (95% CI)**			**234**			**222**	**44.5%**	**-0.08 [-0.59, 0.43]**
Heterogeneity: Tau² = 0.37; Chi² = 104.37, df = 5 (P < 0.00001); I² = 95%								
Test for overall effect: Z = 0.31 (P = 0.76)								
**3.10.2 Severe obesity**								
DIBASY 2012	-1.6	1.6	38	-1.2	1.3	18	5.0%	-0.40 [-1.19, 0.39]
DIBASY 2015	-1.5	1.8	38	-1.6	1.1	15	4.9%	0.10 [-0.70, 0.90]
Dixon 2008	0.1	1.3	30	0	0.8	30	6.4%	0.10 [-0.45, 0.65]
Dixon 2012	0.1	0.4	30	0.4	0.6	30	8.2%	-0.30 [-0.56, -0.04]
Mingrone 2002	-1.2	1	46	0	1.1	33	6.9%	-1.20 [-1.67, -0.73]
SLIMM-T2D 2014/15	-0.1	0.5	37	0.1	0.5	41	8.4%	-0.20 [-0.42, 0.02]
STAMPEDE 2012	-0.1	1.2	99	-0.1	1.1	41	7.3%	0.00 [-0.41, 0.41]
TRAMOMTANA 2015	-0.3	0.5	23	0	0.3	47	8.4%	-0.30 [-0.52, -0.08]
**Subtotal (95% CI)**			**341**			**255**	**55.5%**	**-0.29 [-0.51, -0.06]**
Heterogeneity: Tau² = 0.06; Chi² = 19.64, df = 7 (P = 0.006); I² = 64%								
Test for overall effect: Z = 2.52 (P = 0.01)								
**Total (95% CI)**			**575**			**477**	**100.0%**	**-0.20 [-0.47, 0.06]**
Heterogeneity: Tau² = 0.20; Chi² = 129.16, df = 13 (P < 0.00001); I² = 90%								
Test for overall effect: Z = 1.53 (P = 0.13)								
Test for subgroup differences: Chi² = 0.53, df = 1 (P = 0.47), I² = 0%								

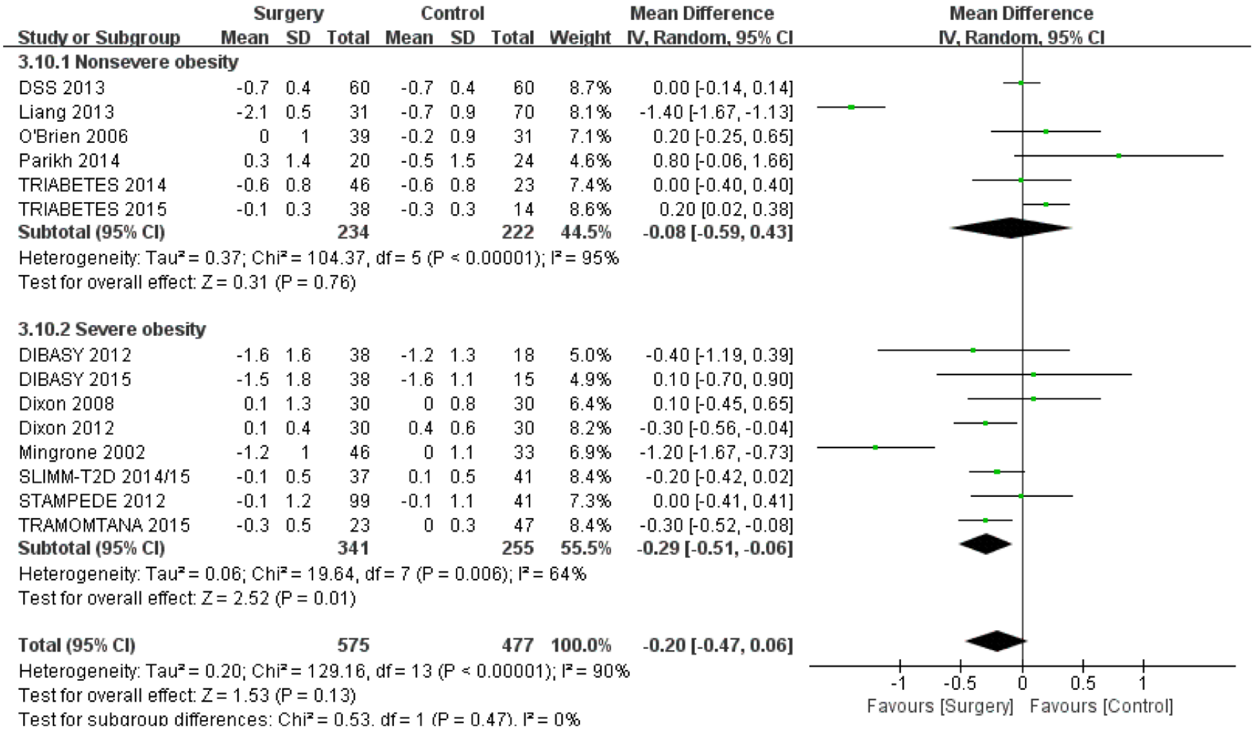

Figure S27.The forest plot of total cholesterol (mmol/L) in terms of obesity levels

Study or Subgroup	Surgery Mean	Surgery SD	Surgery Total	Control Mean	Control SD	Control Total	Weight	Mean Difference IV, Random, 95% CI
**1.11.1 1-year**								
DSS 2013	0.2	0.1	60	0	0.1	60	8.8%	0.20 [0.16, 0.24]
Liang 2013	0.3	0.1	31	0	0.2	70	8.3%	0.30 [0.24, 0.36]
Mingrone 2002	0.4	0.3	46	0	0.2	33	6.8%	0.40 [0.29, 0.51]
Parikh 2014	0.1	0.3	20	0.2	0.7	24	2.5%	-0.10 [-0.41, 0.21]
SLIMM-T2D 2014/15	1.1	0.9	37	0.1	0.1	41	2.7%	1.00 [0.71, 1.29]
STAMPEDE 2012	0.3	0.3	99	0.1	0.2	41	7.6%	0.20 [0.11, 0.29]
TRIABETES 2014	0.3	0.2	46	0.1	0.2	23	7.1%	0.20 [0.10, 0.30]
**Subtotal (95% CI)**			**339**			**292**	**43.7%**	**0.29 [0.19, 0.38]**
Heterogeneity: Tau² = 0.01; Chi² = 49.04, df = 6 (P < 0.00001); I² = 88%								
Test for overall effect: Z = 5.79 (P < 0.00001)								
**1.11.2 2-year**								
DIBASY 2012	0.2	0.3	38	0.1	0.2	18	6.1%	0.10 [-0.03, 0.23]
Dixon 2008	0.3	0.2	30	0	0.1	30	7.7%	0.30 [0.22, 0.38]
Dixon 2012	0.2	0.2	30	0.1	0.1	30	7.7%	0.10 [0.02, 0.18]
O'Brien 2006	0.4	0.4	39	0.1	0.3	31	5.2%	0.30 [0.14, 0.46]
O'Brien 2010	0.2	0.4	24	0.1	0.2	18	4.7%	0.10 [-0.08, 0.28]
TRAMOMTANA 2015	0.2	0.7	23	0.1	0.4	47	2.5%	0.10 [-0.21, 0.41]
**Subtotal (95% CI)**			**184**			**174**	**33.9%**	**0.18 [0.08, 0.27]**
Heterogeneity: Tau² = 0.01; Chi² = 16.83, df = 5 (P = 0.005); I² = 70%								
Test for overall effect: Z = 3.50 (P = 0.0005)								
**1.11.3 3-year or more**								
DIBASY 2015	0.2	0.2	38	0.1	0.1	15	7.7%	0.10 [0.02, 0.18]
STAMPEDE 2014	0.4	0.4	97	0	0.3	40	6.4%	0.40 [0.28, 0.52]
TRIABETES 2015	0.3	0.1	38	0.1	0.1	14	8.2%	0.20 [0.14, 0.26]
**Subtotal (95% CI)**			**173**			**69**	**22.3%**	**0.23 [0.09, 0.36]**
Heterogeneity: Tau² = 0.01; Chi² = 16.05, df = 2 (P = 0.0003); I² = 88%								
Test for overall effect: Z = 3.18 (P = 0.001)								
**Total (95% CI)**			**696**			**535**	**100.0%**	**0.23 [0.18, 0.29]**
Heterogeneity: Tau² = 0.01; Chi² = 86.36, df = 15 (P < 0.00001); I² = 83%								
Test for overall effect: Z = 7.97 (P < 0.00001)								
Test for subgroup differences: Chi² = 2.45, df = 2 (P = 0.29), I² = 18.5%								

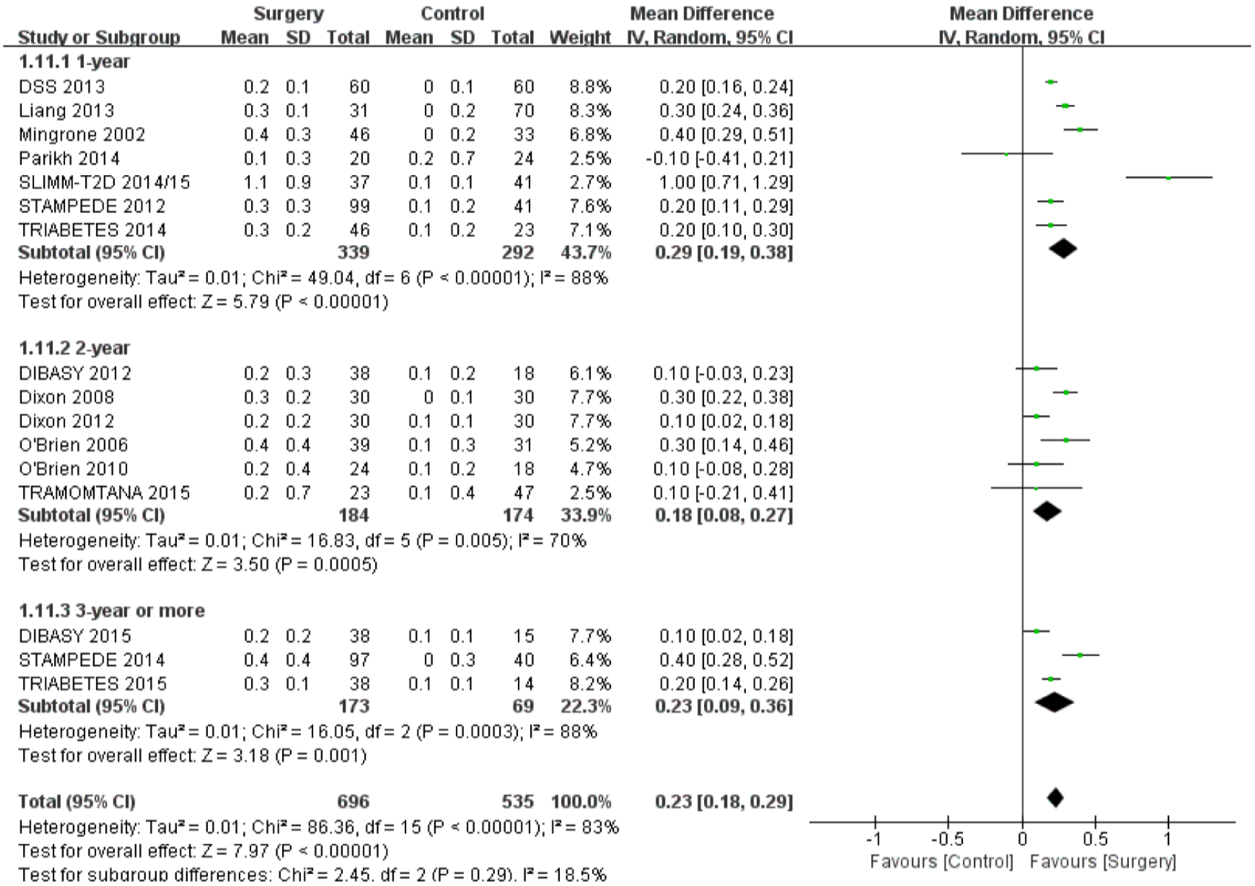

Figure S28.The forest plot of high density lipoprotein (mmol/L) in terms of follow-up duration

Study or Subgroup	Surgery Mean	Surgery SD	Surgery Total	Control Mean	Control SD	Control Total	Weight	Mean Difference IV, Random, 95% CI
**2.11.1 Sleeve gastrectomy**								
STAMPEDE 2012	0.3	0.3	49	0.1	0.2	41	4.6%	0.20 [0.10, 0.30]
STAMPEDE 2014	0.4	0.4	49	0	0.3	40	4.5%	0.40 [0.25, 0.55]
**Subtotal (95% CI)**			**98**			**81**	**9.1%**	**0.29 [0.10, 0.49]**
Heterogeneity: Tau² = 0.02; Chi² = 4.80, df = 1 (P = 0.03); I² = 79%								
Test for overall effect: Z = 2.94 (P = 0.003)								
**2.11.2 Roux-en-Y gastric bypass**								
DIBASY 2012	0.3	0.3	19	0.1	0.2	18	4.5%	0.20 [0.04, 0.36]
DIBASY 2015	0.3	0.2	19	0.1	0.1	15	4.6%	0.20 [0.10, 0.30]
DSS 2013	0.2	0.1	60	0	0.1	60	4.6%	0.20 [0.16, 0.24]
Liang 2013	0.3	0.1	31	0	0.2	70	4.6%	0.30 [0.24, 0.36]
SLIMM-T2D 2014	0.3	0.1	19	0	0.1	19	4.6%	0.30 [0.24, 0.36]
STAMPEDE 2012	0.3	0.3	50	0.1	0.2	41	4.6%	0.20 [0.10, 0.30]
STAMPEDE 2014	0.4	0.4	48	0	0.3	40	4.5%	0.40 [0.25, 0.55]
TRIABETES 2014	0.3	0.2	24	0.1	0.2	23	4.6%	0.20 [0.09, 0.31]
TRIABETES 2015	0.4	0.1	18	0.1	0.1	14	4.6%	0.30 [0.23, 0.37]
**Subtotal (95% CI)**			**288**			**300**	**41.1%**	**0.26 [0.21, 0.30]**
Heterogeneity: Tau² = 0.00; Chi² = 21.36, df = 8 (P = 0.006); I² = 63%								
Test for overall effect: Z = 11.43 (P < 0.00001)								
**2.11.3 Laparoscopic adjustable gastric banding**								
Dixon 2008	0.3	0.2	30	0	0.1	30	4.6%	0.30 [0.22, 0.38]
Dixon 2012	0.2	0.2	30	0.1	0.1	30	4.6%	0.10 [0.02, 0.18]
O'Brien 2006	0.4	0.4	39	0.1	0.3	31	4.5%	0.30 [0.14, 0.46]
O'Brien 2010	0.2	0.4	24	0.1	0.2	18	4.5%	0.10 [-0.08, 0.28]
SLIMM-T2D 2015	2	0.1	18	0.1	0.1	22	4.6%	1.90 [1.84, 1.96]
TRIABETES 2014	0.2	0.2	22	0.1	0.2	23	4.6%	0.10 [-0.02, 0.22]
TRIABETES 2015	0.3	0.1	20	0.1	0.1	14	4.6%	0.20 [0.13, 0.27]
**Subtotal (95% CI)**			**183**			**168**	**31.9%**	**0.43 [-0.21, 1.07]**
Heterogeneity: Tau² = 0.74; Chi² = 2104.16, df = 6 (P < 0.00001); I² = 100%								
Test for overall effect: Z = 1.32 (P = 0.19)								
**2.11.4 Biliopancreatic diversion**								
DIBASY 2012	0.1	0.2	19	0.1	0.2	18	4.5%	0.00 [-0.13, 0.13]
DIBASY 2015	0.1	0.2	19	0.1	0.2	15	4.6%	0.00 [-0.10, 0.10]
Mingrone 2002	0.4	0.3	46	0	0.2	33	4.6%	0.40 [0.29, 0.51]
TRAMOMTANA 2015	0.2	0.7	23	0.1	0.4	47	4.2%	0.10 [-0.21, 0.41]
**Subtotal (95% CI)**			**107**			**113**	**17.9%**	**0.13 [-0.10, 0.35]**
Heterogeneity: Tau² = 0.05; Chi² = 32.99, df = 3 (P < 0.00001); I² = 91%								
Test for overall effect: Z = 1.10 (P = 0.27)								
**Total (95% CI)**			**676**			**662**	**100.0%**	**0.29 [0.09, 0.50]**
Heterogeneity: Tau² = 0.24; Chi² = 2645.17, df = 21 (P < 0.00001); I² = 99%								
Test for overall effect: Z = 2.78 (P = 0.005)								
Test for subgroup differences: Chi² = 1.64, df = 3 (P = 0.65), I² = 0%								

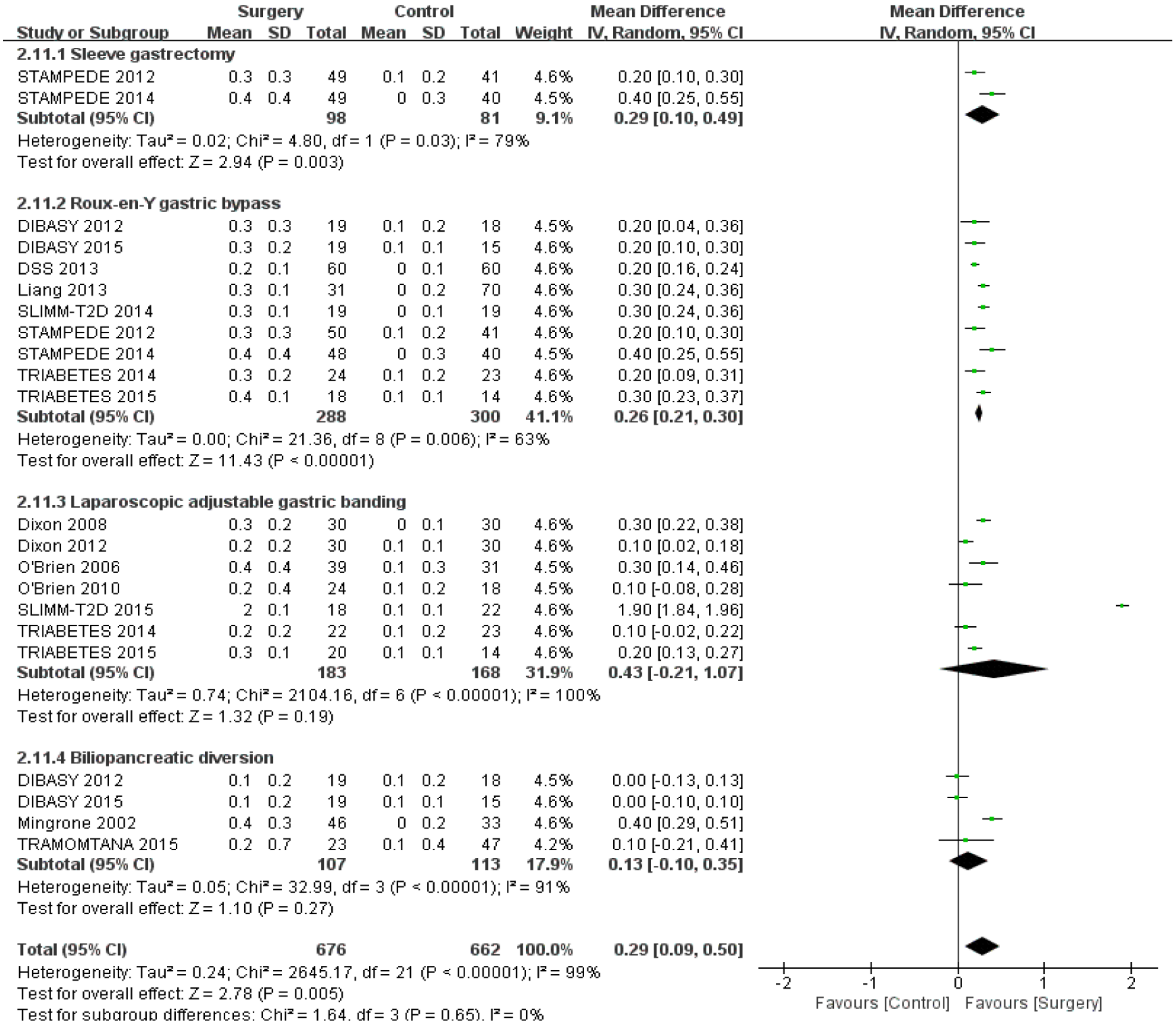

Figure S29.The forest plot of high density lipoprotein (mmol/L) in terms of surgical techniques

Study or Subgroup	Surgery Mean	Surgery SD	Surgery Total	Control Mean	Control SD	Control Total	Weight	Mean Difference IV, Random, 95% CI
**3.11.1 Nonsevere obesity**								
DSS 2013	0.2	0.1	60	0	0.1	60	8.8%	0.20 [0.16, 0.24]
Liang 2013	0.3	0.1	31	0	0.2	70	8.3%	0.30 [0.24, 0.36]
O'Brien 2006	0.4	0.4	39	0.1	0.3	31	5.2%	0.30 [0.14, 0.46]
Parikh 2014	0.1	0.3	20	0.2	0.7	24	2.5%	-0.10 [-0.41, 0.21]
TRIABETES 2014	0.3	0.2	46	0.1	0.2	23	7.1%	0.20 [0.10, 0.30]
TRIABETES 2015	0.3	0.1	38	0.1	0.1	14	8.2%	0.20 [0.14, 0.26]
**Subtotal (95% CI)**			**234**			**222**	**40.1%**	**0.22 [0.17, 0.28]**
Heterogeneity: Tau² = 0.00; Chi² = 13.94, df = 5 (P = 0.02); I² = 64%								
Test for overall effect: Z = 8.04 (P < 0.00001)								
**3.11.2 Severe obesity**								
DIBASY 2012	0.2	0.3	38	0.1	0.2	18	6.1%	0.10 [-0.03, 0.23]
DIBASY 2015	0.2	0.2	38	0.1	0.1	15	7.7%	0.10 [0.02, 0.18]
Dixon 2008	0.3	0.2	30	0	0.1	30	7.7%	0.30 [0.22, 0.38]
Dixon 2012	0.2	0.2	30	0.1	0.1	30	7.7%	0.10 [0.02, 0.18]
Mingrone 2002	0.4	0.3	46	0	0.2	33	6.8%	0.40 [0.29, 0.51]
O'Brien 2010	0.2	0.4	24	0.1	0.2	18	4.7%	0.10 [-0.08, 0.28]
SLIMM-T2D 2014/15	1.1	0.9	37	0.1	0.1	41	2.7%	1.00 [0.71, 1.29]
STAMPEDE 2012	0.3	0.3	99	0.1	0.2	41	7.6%	0.20 [0.11, 0.29]
STAMPEDE 2014	0.4	0.4	97	0	0.3	40	6.4%	0.40 [0.28, 0.52]
TRAMOMTANA 2015	0.2	0.7	23	0.1	0.4	47	2.5%	0.10 [-0.21, 0.41]
**Subtotal (95% CI)**			**462**			**313**	**59.9%**	**0.26 [0.15, 0.36]**
Heterogeneity: Tau² = 0.02; Chi² = 72.39, df = 9 (P < 0.00001); I² = 88%								
Test for overall effect: Z = 4.84 (P < 0.00001)								
**Total (95% CI)**			**696**			**535**	**100.0%**	**0.23 [0.18, 0.29]**
Heterogeneity: Tau² = 0.01; Chi² = 86.36, df = 15 (P < 0.00001); I² = 83%								
Test for overall effect: Z = 7.97 (P < 0.00001)								
Test for subgroup differences: Chi² = 0.32, df = 1 (P = 0.57), I² = 0%								

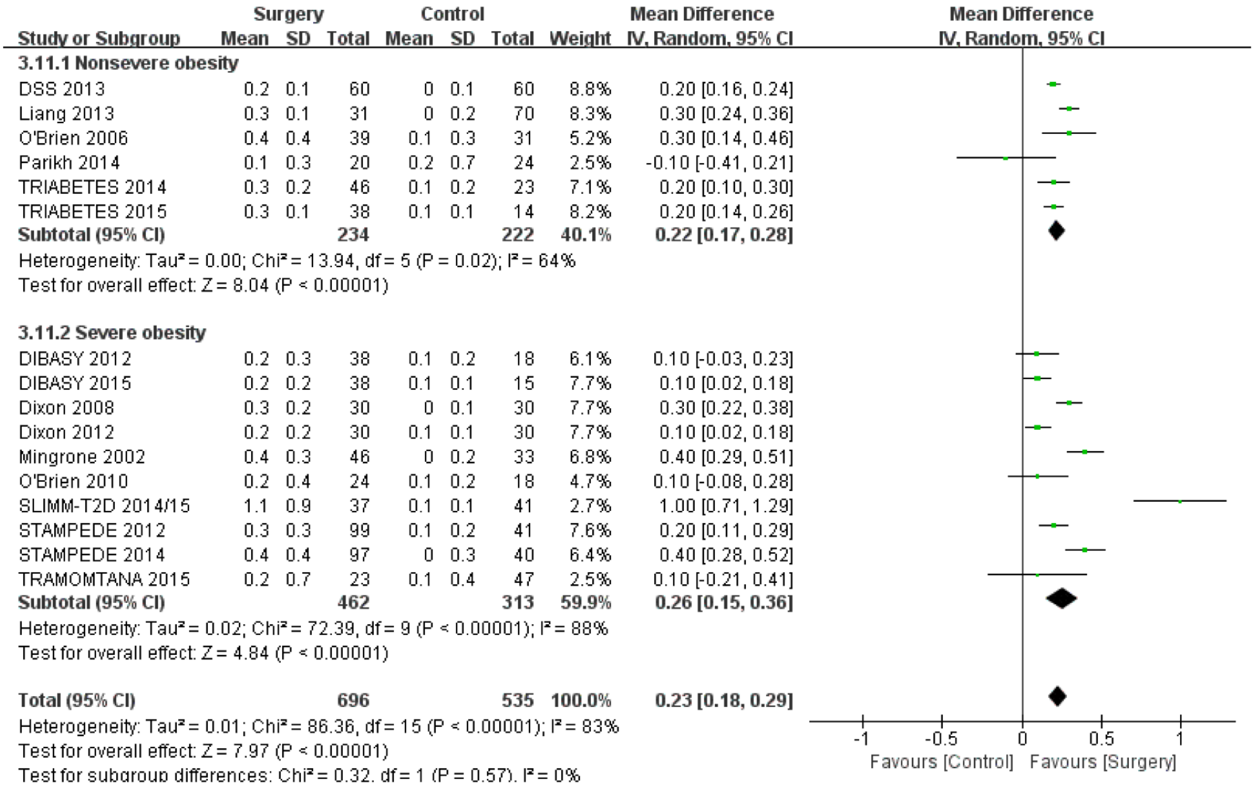

Figure S30.The forest plot of high density lipoprotein (mmol/L) in terms of obesity levels

	Surgery			Control				Mean Difference
Study or Subgroup	Mean	SD	Total	Mean	SD	Total	Weight	IV, Random, 95% CI
**1.12.1 1-year**								
DSS 2013	-1.1	0.7	60	-0.9	0.8	60	8.3%	-0.20 [-0.47, 0.07]
Liang 2013	-1.9	0.6	31	-0.5	0.7	70	8.3%	-1.40 [-1.67, -1.13]
Mingrone 2002	-1.2	1.2	46	0	1	33	7.4%	-1.20 [-1.69, -0.71]
Parikh 2014	0.5	1.2	20	-0.3	1.4	24	6.0%	0.80 [0.03, 1.57]
SLIMM-T2D 2014/15	-0.1	0.5	37	0.1	0.4	41	8.5%	-0.20 [-0.40, 0.00]
STAMPEDE 2012	0.1	1	99	-0.1	1	41	8.0%	0.20 [-0.16, 0.56]
TRIABETES 2014	-0.1	1	46	-0.2	0.9	23	7.5%	0.10 [-0.37, 0.57]
**Subtotal (95% CI)**			**339**			**292**	**54.1%**	**-0.30 [-0.81, 0.20]**
Heterogeneity: Tau² = 0.42; Chi² = 94.15, df = 6 (P < 0.00001); I² = 94%								
Test for overall effect: Z = 1.18 (P = 0.24)								
**1.12.2 2-year**								
DIBASY 2012	-1.4	1.2	38	-1	1.2	18	6.5%	-0.40 [-1.07, 0.27]
O'Brien 2006	-0.2	0.7	39	-0.2	0.8	31	8.0%	0.00 [-0.36, 0.36]
TRAMOMTANA 2015	-0.7	0.6	23	0	0.5	47	8.3%	-0.70 [-0.98, -0.42]
**Subtotal (95% CI)**			**100**			**96**	**22.8%**	**-0.37 [-0.87, 0.13]**
Heterogeneity: Tau² = 0.15; Chi² = 9.05, df = 2 (P = 0.01); I² = 78%								
Test for overall effect: Z = 1.46 (P = 0.14)								
**1.12.3 3-year or more**								
DIBASY 2015	-1.3	1.5	38	-1.4	1	15	6.4%	0.10 [-0.60, 0.80]
STAMPEDE 2014	0.1	0.9	97	0	1	40	8.0%	0.10 [-0.26, 0.46]
TRIABETES 2015	0.1	0.2	38	-0.2	0.2	14	8.7%	0.30 [0.18, 0.42]
**Subtotal (95% CI)**			**173**			**69**	**23.1%**	**0.27 [0.16, 0.39]**
Heterogeneity: Tau² = 0.00; Chi² = 1.32, df = 2 (P = 0.52); I² = 0%								
Test for overall effect: Z = 4.70 (P < 0.00001)								
**Total (95% CI)**			**612**			**457**	**100.0%**	**-0.21 [-0.54, 0.12]**
Heterogeneity: Tau² = 0.33; Chi² = 181.15, df = 12 (P < 0.00001); I² = 93%								
Test for overall effect: Z = 1.25 (P = 0.21)								
Test for subgroup differences: Chi² = 10.40, df = 2 (P = 0.006), I² = 80.8%								

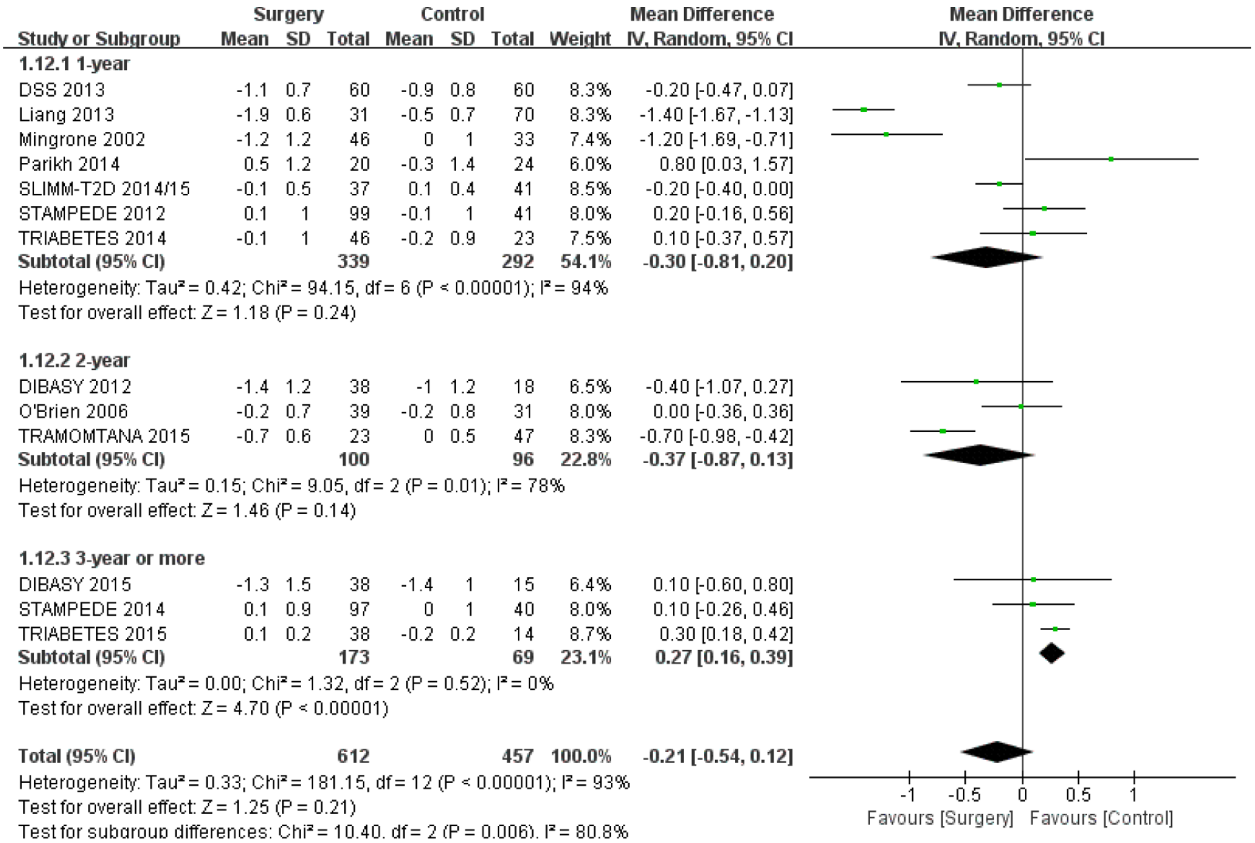

Figure S31.The forest plot of low density lipoprotein (mmol/L) in terms of follow-up duration

Study or Subgroup	Surgery Mean	Surgery SD	Surgery Total	Control Mean	Control SD	Control Total	Weight	Mean Difference IV, Random, 95% CI
**2.12.1 Sleeve gastrectomy**								
STAMPEDE 2012	0.1	1.1	49	-0.1	1	41	5.3%	0.20 [-0.23, 0.63]
STAMPEDE 2014	0.1	1	49	0	1	40	5.3%	0.10 [-0.32, 0.52]
**Subtotal (95% CI)**			**98**			**81**	**10.6%**	**0.15 [-0.15, 0.45]**
Heterogeneity: Tau² = 0.00; Chi² = 0.11, df = 1 (P = 0.74); I² = 0%								
Test for overall effect: Z = 0.96 (P = 0.34)								
**2.12.2 Roux-en-Y gastric bypass**								
DIBASY 2012	-0.6	0.8	19	-1	1.2	18	4.4%	0.40 [-0.26, 1.06]
DIBASY 2015	-0.4	1.2	19	-1.4	1	15	4.1%	1.00 [0.26, 1.74]
DSS 2013	-1.1	0.7	60	-0.9	0.8	60	5.8%	-0.20 [-0.47, 0.07]
Liang 2013	-1.9	0.6	31	-0.5	0.7	70	5.8%	-1.40 [-1.67, -1.13]
SLIMM-T2D 2014	-0.1	0.5	19	0.2	0.5	19	5.6%	-0.30 [-0.62, 0.02]
STAMPEDE 2012	0.1	0.9	50	-0.1	1	41	5.4%	0.20 [-0.19, 0.59]
STAMPEDE 2014	0.1	0.7	48	0	1	40	5.5%	0.10 [-0.27, 0.47]
TRIABETES 2014	-0.6	0.8	24	-0.2	0.9	23	5.1%	-0.40 [-0.89, 0.09]
TRIABETES 2015	0	0.2	18	-0.2	0.2	14	6.1%	0.20 [0.06, 0.34]
**Subtotal (95% CI)**			**288**			**300**	**47.8%**	**-0.08 [-0.51, 0.34]**
Heterogeneity: Tau² = 0.37; Chi² = 126.04, df = 8 (P < 0.00001); I² = 94%								
Test for overall effect: Z = 0.37 (P = 0.71)								
**2.12.3 Laparoscopic adjustable gastric banding**								
O'Brien 2006	-0.2	0.7	39	-0.2	0.8	31	5.5%	0.00 [-0.36, 0.36]
SLIMM-T2D 2015	-0.2	0.4	18	0.1	0.4	22	5.8%	-0.30 [-0.55, -0.05]
TRIABETES 2014	0.4	0.9	22	-0.2	0.9	23	4.9%	0.60 [0.07, 1.13]
TRIABETES 2015	0.2	0.2	20	-0.2	0.2	14	6.1%	0.40 [0.26, 0.54]
**Subtotal (95% CI)**			**99**			**90**	**22.4%**	**0.15 [-0.26, 0.57]**
Heterogeneity: Tau² = 0.15; Chi² = 26.82, df = 3 (P < 0.00001); I² = 89%								
Test for overall effect: Z = 0.74 (P = 0.46)								
**2.12.4 Biliopancreatic diversion**								
DIBASY 2012	-2.2	1.1	19	-1	1.2	18	4.1%	-1.20 [-1.94, -0.46]
DIBASY 2015	-2.2	1.1	19	-1.4	1	15	4.3%	-0.80 [-1.51, -0.09]
Mingrone 2002	-1.2	1.2	46	0	1	33	5.1%	-1.20 [-1.69, -0.71]
TRAMOMTANA 2015	-0.7	0.6	23	0	0.5	47	5.7%	-0.70 [-0.98, -0.42]
**Subtotal (95% CI)**			**107**			**113**	**19.2%**	**-0.90 [-1.18, -0.62]**
Heterogeneity: Tau² = 0.02; Chi² = 3.93, df = 3 (P = 0.27); I² = 24%								
Test for overall effect: Z = 6.29 (P < 0.00001)								
**Total (95% CI)**			**592**			**584**	**100.0%**	**-0.17 [-0.44, 0.09]**
Heterogeneity: Tau² = 0.29; Chi² = 235.79, df = 18 (P < 0.00001); I² = 92%								
Test for overall effect: Z = 1.30 (P = 0.19)								
Test for subgroup differences: Chi² = 31.65, df = 3 (P < 0.00001), I² = 90.5%								

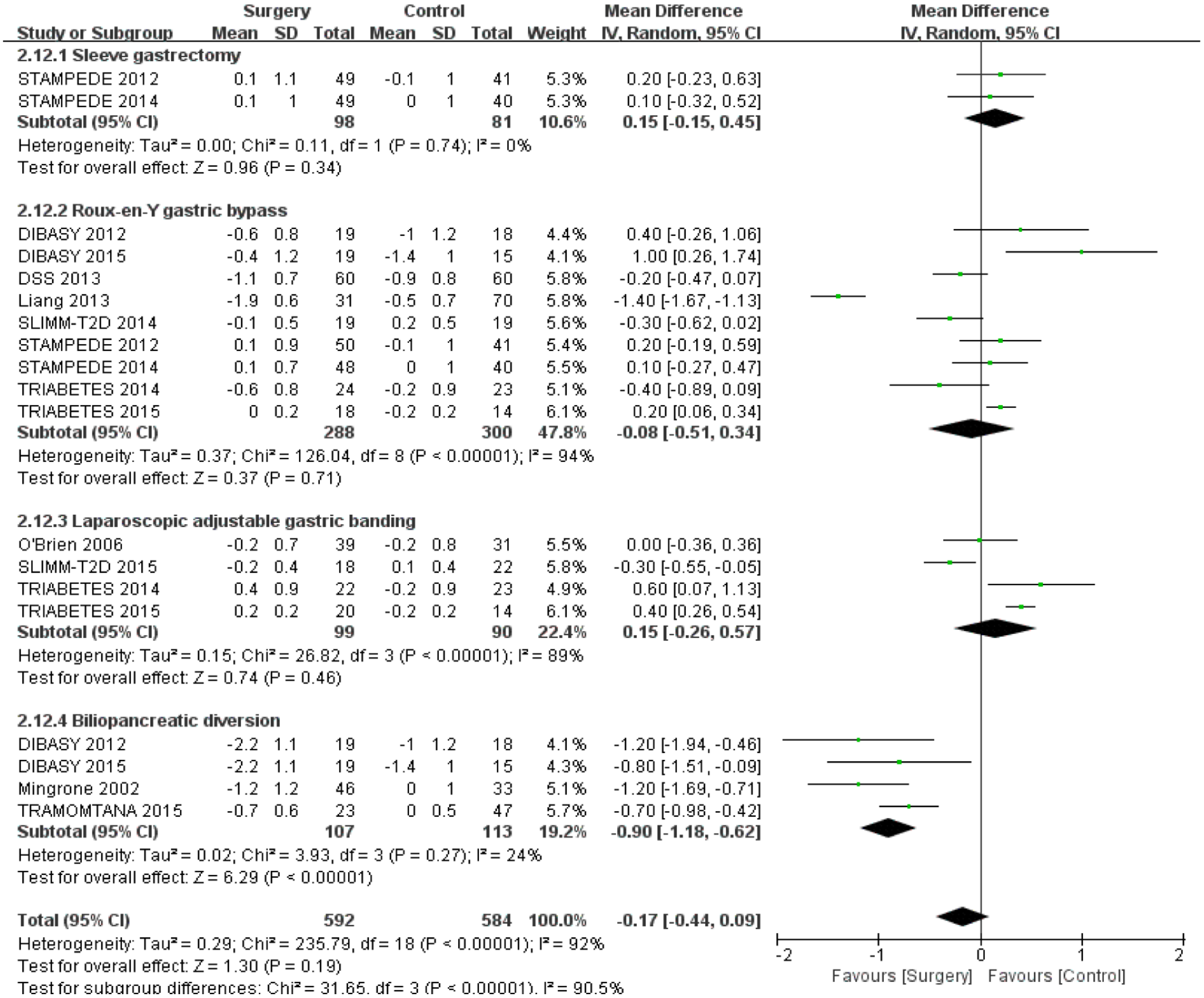

Figure S32.The forest plot of low density lipoprotein (mmol/L) in terms of surgical techniques

Study or Subgroup	Surgery Mean	Surgery SD	Surgery Total	Control Mean	Control SD	Control Total	Weight	Mean Difference IV, Random, 95% CI
**3.12.1 Nonsevere obesity**								
DSS 2013	-1.1	0.7	60	-0.9	0.8	60	8.3%	-0.20 [-0.47, 0.07]
Liang 2013	-1.9	0.6	31	-0.5	0.7	70	8.3%	-1.40 [-1.67, -1.13]
O'Brien 2006	-0.2	0.7	39	-0.2	0.8	31	8.0%	0.00 [-0.36, 0.36]
Parikh 2014	0.5	1.2	20	-0.3	1.4	24	6.0%	0.80 [0.03, 1.57]
TRIABETES 2014	-0.1	1	46	-0.2	0.9	23	7.5%	0.10 [-0.37, 0.57]
TRIABETES 2015	0.1	0.2	38	-0.2	0.2	14	8.7%	0.30 [0.18, 0.42]
**Subtotal (95% CI)**			**234**			**222**	**46.9%**	**-0.10 [-0.70, 0.51]**
Heterogeneity: Tau² = 0.53; Chi² = 134.78, df = 5 (P < 0.00001); I² = 96%								
Test for overall effect: Z = 0.31 (P = 0.76)								
**3.12.2 Severe obesity**								
DIBASY 2012	-1.4	1.2	38	-1	1.2	18	6.5%	-0.40 [-1.07, 0.27]
DIBASY 2015	-1.3	1.5	38	-1.4	1	15	6.4%	0.10 [-0.60, 0.80]
Mingrone 2002	-1.2	1.2	46	0	1	33	7.4%	-1.20 [-1.69, -0.71]
SLIMM-T2D 2014/15	-0.1	0.5	37	0.1	0.4	41	8.5%	-0.20 [-0.40, 0.00]
STAMPEDE 2012	0.1	1	99	-0.1	1	41	8.0%	0.20 [-0.16, 0.56]
STAMPEDE 2014	0.1	0.9	97	0	1	40	8.0%	0.10 [-0.26, 0.46]
TRAMOMTANA 2015	-0.7	0.6	23	0	0.5	47	8.3%	-0.70 [-0.98, -0.42]
**Subtotal (95% CI)**			**378**			**235**	**53.1%**	**-0.30 [-0.64, 0.04]**
Heterogeneity: Tau² = 0.16; Chi² = 35.03, df = 6 (P < 0.00001); I² = 83%								
Test for overall effect: Z = 1.71 (P = 0.09)								
**Total (95% CI)**			**612**			**457**	**100.0%**	**-0.21 [-0.54, 0.12]**
Heterogeneity: Tau² = 0.33; Chi² = 181.15, df = 12 (P < 0.00001); I² = 93%								
Test for overall effect: Z = 1.25 (P = 0.21)								
Test for subgroup differences: Chi² = 0.33, df = 1 (P = 0.57), I² = 0%								

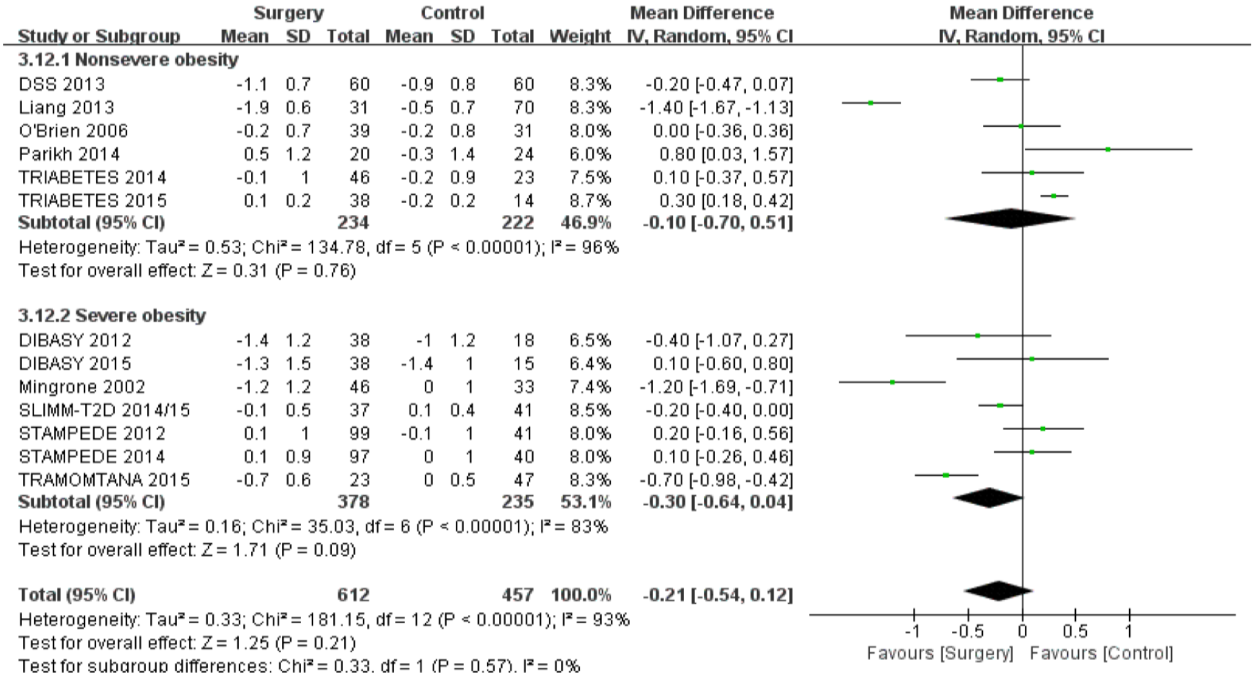

Figure S33.The forest plot of low density lipoprotein (mmol/L) in terms of obesity levels

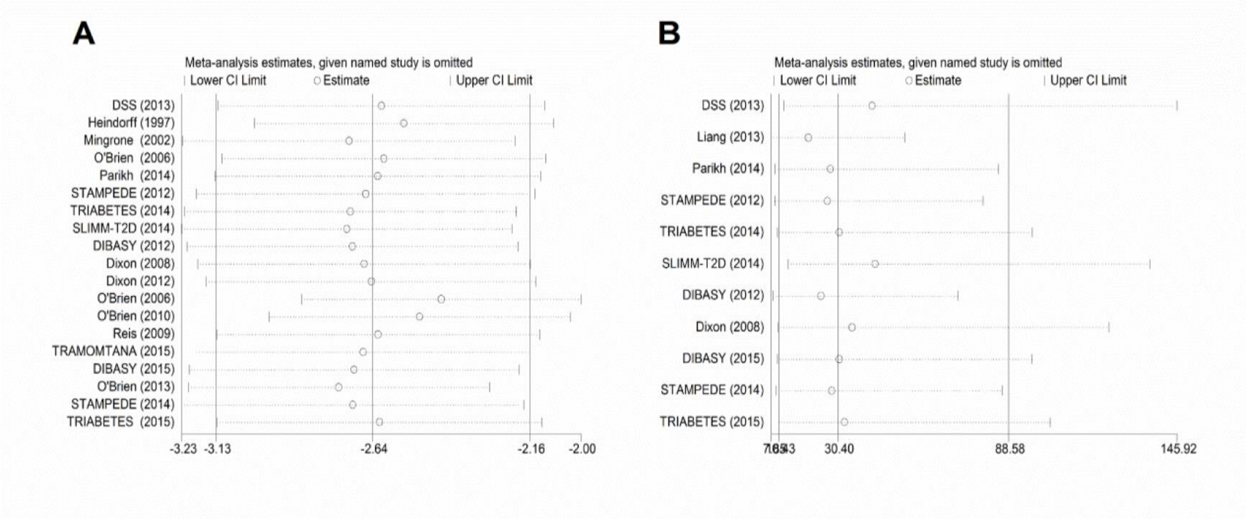

Figure S34.Sensitivity analysis

A. Weight loss; B. Diabetic remission

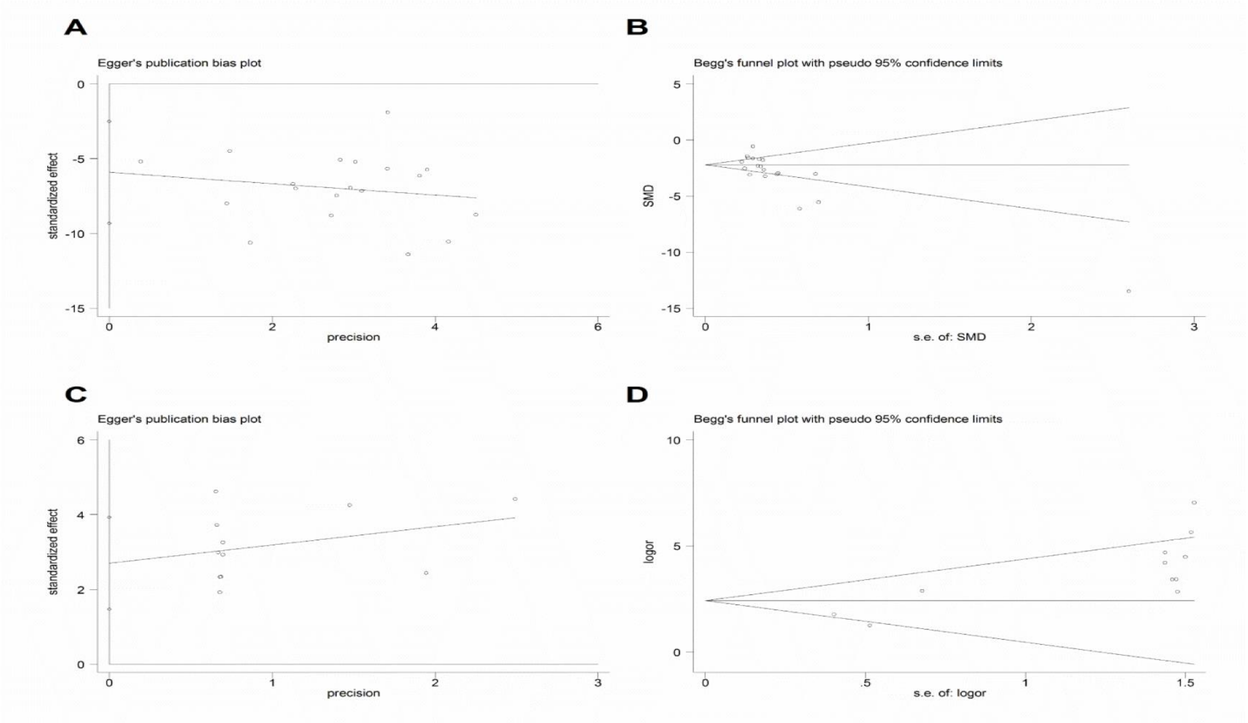

Figure S35.Analysis of publication bias

A. Egger's test of weight loss; B. Begg's test of weight loss; C. Egger's test of diabetic remission; D. Begg's test of diabetic remission;

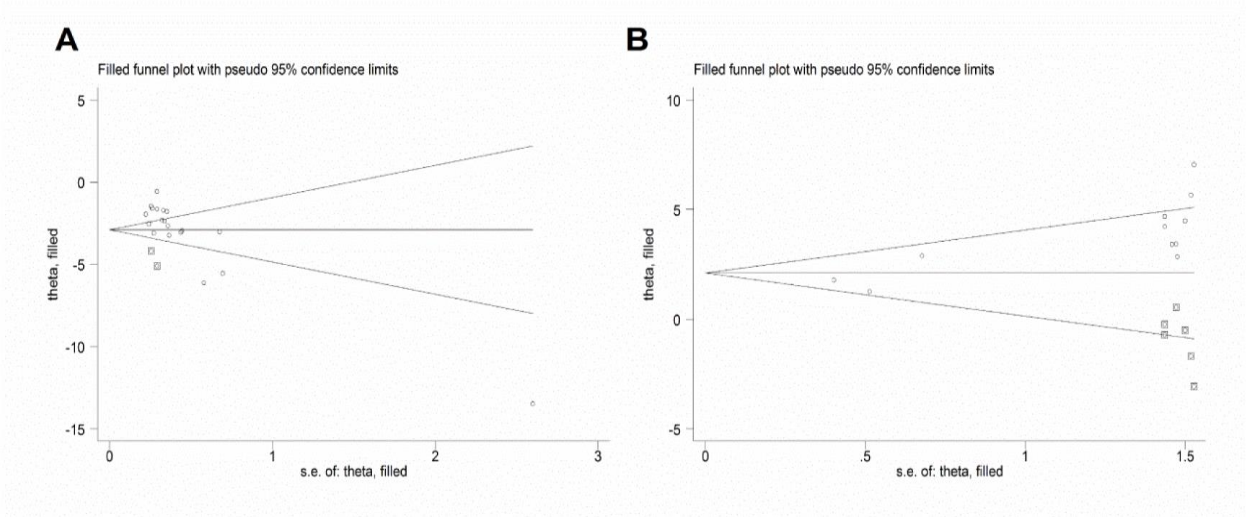

Figure S36.Trim-and-Fill method
A. Weight loss; B. Diabetic remission;
